# Identification of genes required for eye development by high-throughput screening of mouse knockouts

**DOI:** 10.1038/s42003-018-0226-0

**Published:** 2018-12-21

**Authors:** Bret A. Moore, Brian C. Leonard, Lionel Sebbag, Sydney G. Edwards, Ann Cooper, Denise M. Imai, Ewan Straiton, Luis Santos, Christopher Reilly, Stephen M. Griffey, Lynette Bower, David Clary, Jeremy Mason, Michel J. Roux, Hamid Meziane, Yann Herault, International Mouse Phenotyping Consortium, Anna Swan, Ruairidh King, Piia Keskivali-Bond, Lois Kelsey, Igor Vukobradovic, Dawei Qu, Ruolin Guo, Elisa Tran, Lily Morikawa, Milan Ganguly, Napoleon Law, Xueyuan Shang, Patricia Feugas, Yanchun Wang, Yingchun Zhu, Kyle Duffin, Ayexa Ramirez, Patricia Penton, Valerie Laurin, Shannon Clarke, Qing Lan, Gillian Sleep, Amie Creighton, Elsa Jacob, Ozge Danisment, Joanna Joeng, Marina Gertsenstein, Monica Pereira, Sue MacMaster, Sandra Tondat, Tracy Carroll, Jorge Cabezas, Amit Patel, Jane Hunter, Gregory Clark, Mohammed Bubshait, David Miller, Khondoker Sohel, Alexandr Bezginov, Matthew McKay, Kevin Peterson, Leslie Goodwin, Rachel Urban, Susan Kales, Rob Hallett, Dong Nguyen-Bresinsky, Timothy Leach, Audrie Seluke, Sara Perkins, Amanda Slater, Rick Bedigian, Leah Rae Donahue, Robert Taft, James Denegre, Zachery Seavey, Amelia Willett, Lindsay Bates, Leslie Haynes, Julie Creed, Catherine Witmeyer, Willson Roper, James Clark, Pamela Stanley, Samantha Burrill, Jennifer Ryan, Yuichi Obata, Masaru Tamura, Hideki Kaneda, Tamio Furuse, Kimio Kobayashi, Ikuo Miura, Ikuko Yamada, Hiroshi Masuya, Nobuhiko Tanaka, Shinya Ayabe, Atsushi Yoshiki, Valerie Vancollie, Francesco Chiani, Chiara Di Pietro, Gianfranco Di Segni, Olga Ermakova, Filomena Ferrara, Paolo Fruscoloni, Alessia Gambadoro, Serena Gastaldi, Elisabetta Golini, Gina La Sala, Silvia Mandillo, Daniela Marazziti, Marzia Massimi, Rafaele Matteoni, Tiziana Orsini, Miriam Pasquini, Marcello Raspa, Aline Rauch, Gianfranco Rossi, Nicoletta Rossi, Sabrina Putti, Ferdinando Scavizzi, Giuseppe D. Tocchini-Valentini, Colin McKerlie, Ann M. Flenniken, Lauryl M. J. Nutter, Zorana Berberovic, Celeste Owen, Susan Newbigging, Hibret Adissu, Mohammed Eskandarian, Chih-Wei Hsu, Sowmya Kalaga, Uchechukwu Udensi, Chinwe Asomugha, Ritu Bohat, Juan J. Gallegos, John R. Seavitt, Jason D. Heaney, Arthur L. Beaudet, Mary E. Dickinson, Monica J. Justice, Vivek Philip, Vivek Kumar, Karen L. Svenson, Robert E. Braun, Sara Wells, Heather Cater, Michelle Stewart, Sharon Clementson-Mobbs, Russell Joynson, Xiang Gao, Tomohiro Suzuki, Shigeharu Wakana, Damian Smedley, J. K Seong, Glauco Tocchini-Valentini, Mark Moore, Colin Fletcher, Natasha Karp, Ramiro Ramirez-Solis, Jacqueline K. White, Martin Hrabe de Angelis, Wolfgang Wurst, Sara M. Thomasy, Paul Flicek, Helen Parkinson, Steve D. M. Brown, Terrence F. Meehan, Patsy M. Nishina, Stephen A. Murray, Mark P. Krebs, Ann-Marie Mallon, K. C. Kent Lloyd, Christopher J. Murphy, Ala Moshiri

**Affiliations:** 1 William R. Pritchard Veterinary Medical Teaching Hospital, School of Veterinary Medicine, University of California-Davis, Davis, 95616 CA USA; 2 Department of Surgical and Radiological Sciences, School of Veterinary Medicine, University of California-Davis, Davis, CA 95616 USA; 3 Comparative Pathology Laboratory, School of Veterinary Medicine, University of California-Davis, Davis, CA 95616 USA; 4 Medical Research Council Harwell Institute (Mammalian Genetis Unit and Mary Lyon Center, Harwell, Oxfordshire OX11 0RD UK; 5 Mouse Biology Program, and Department of Surgery, School of Medicine, University of California-Davis, Davis, CA 95618 USA; 6 European Molecular Biology Laboratory, European Bioinformatics Institute, Wellcome Genome Campus, Hinxton, Cambridge, CB10 1 SD UK; 7 Institut de Génétique et de Biologie Moléculaire et Cellulaire, Université de Strasbourg, 1 rue Laurent Fries, 67404 Illkirch, France; 8 Centre National de la Recherche Scientifique, UMR7104 Illkirch, France; 9 Institut National de la Santé et de la Recherche Médicale, U1258 Illkirch, France; 10 Université de Strasbourg, 1 rue Laurent Fries, 67404 Illkirch, France; 11 CELPHEDIA, PHENOMIN, Institut Clinique de la Souris (ICS), CNRS, INSERM, University of Strasbourg, 1 rue Laurent Fries, 67404 Illkirch-Graffenstaden, France; 12 The Centre for Phenogenomics, Toronto, ON M5T 3H7 Canada; 13 The Hospital for Sick Children, Toronto, ON M5G 1X8 Canada; 14 Lunenfeld-Tanenbaum Research Institute, Mount Sinai Hospital, Toronto, ON M5G 1X5 Canada; 15 Department of Molecular Physiology and Biophysics, Baylor College of Medicine, Houston, TX 77030 USA; 16 Department of Molecular and Human Genetics, Baylor College of Medicine, Houston, TX 77030 USA; 17 The Jackson Laboratory, Bar Harbor, ME 04609 USA; 18 SKL of Pharmaceutical Biotechnology and Model Animal Research Center, Collaborative Innovation Center for Genetics and Development, Nanjing Biomedical Research Institute, Nanjing University, Nanjing, 210061 China; 19 RIKEN BioResource Center, Tsukuba, Ibaraki 305-0074 Japan; 20 Clinical Pharmacology, Charterhouse Square, Barts and the London School of Medicine and Dentistry, Queen Mary University of London, London, EC1M 6BQ UK; 21 Korea Mouse Phenotyping Consortium (KMPC) and BK21 Program for Veterinary Science, Research Institute for Veterinary Science, College of Veterinary Medicine, Seoul National University, 599 Gwanangno, Gwanak-gu, Seoul, 08826 South Korea; 22 Monterotondo Mouse Clinic, Italian National Research Council (CNR), Institute of Cell Biology and Neurobiology, Adriano Buzzati-Traverso Campus, Via Ramarini, I-00015 Monterotondo Scalo, Italy; 23 International Mouse Phenotyping Consortium, San Anselmo, CA 94960 USA; 24 National Institutes of Health, Bethesda, MD 20205 USA; 25 The Wellcome Trust Sanger Institute, Wellcome Genome Campus, Hinxton, Cambridge, CB10 1SA UK; 26 German Mouse Clinic, Institute of Experimental Genetics, Helmholtz Zentrum München, German Research Center for Environmental Health, Ingolstädter Landstraße 1, 85764 Neuherberg, Germany; 27 Department of Ophthalmology & Vision Science, School of Medicine, U.C. Davis, Sacramento, CA 95817 USA; 28 Monterotondo Mouse Clinic, Italian National Research Council (CNR), Institute of Cell Biology and Neurobiology, Adriano Buzzati-Traverso Campus, Via Ramarini, I-00015 Monterotondo Scalo, Italy

## Abstract

Despite advances in next generation sequencing technologies, determining the genetic basis of ocular disease remains a major challenge due to the limited access and prohibitive cost of human forward genetics. Thus, less than 4,000 genes currently have available phenotype information for any organ system. Here we report the ophthalmic findings from the International Mouse Phenotyping Consortium, a large-scale functional genetic screen with the goal of generating and phenotyping a null mutant for every mouse gene. Of 4364 genes evaluated, 347 were identified to influence ocular phenotypes, 75% of which are entirely novel in ocular pathology. This discovery greatly increases the current number of genes known to contribute to ophthalmic disease, and it is likely that many of the genes will subsequently prove to be important in human ocular development and disease.

## Abstract (web summary)

Bret Moore et al. from the International Mouse Phenotyping Consortium report the identification of 347 mouse genes that influence ocular phenotypes when knocked out. 75% of the identified genes have not previously been associated with any ocular pathology.

## Introduction

The prevalence and burden of ophthalmic disease within the human population, some with the potential for causing complete blindness, highlights the need to identify factors that cause such conditions^1–3^. A wide variety of ocular diseases are known to have an underlying genetic component. These include single-gene disorders^4^ and multi-factorial ocular disorders including age-related diseases with hereditary predispositions embedded in several risk alleles across the genome^5^. However, the genetic contribution(s) for many ocular diseases remains largely unknown or poorly understood^4^. Phenotype information of any organ system is available for approximately 4000 genes at Online Mendelian Inheritance in Man (https://www.omim.org/), illustrating the limited access and the prohibitive cost of forward genetics in humans, despite advances in next generation sequencing technologies. Altogether, the limitations on genetic research in humans, the genetic variability between individuals and among populations, the rarity of many diseases, and the size of the mammalian genome together make identification of disease-causing alleles challenging.

Classical genetic techniques studying pedigrees of human families affected by ocular disorders have identified numerous genes associated with a wide array of eye diseases (e.g., see Retinal Information Network - https://sph.uth.edu/retnet/). However, gene discovery by pedigree analysis is limited. Studies exploring genetic mechanisms in cellular biology have traditionally relied upon single-gene deletions in animal models (largely mice) targeted by individual laboratories, and by identification of gene mutations in mutagenesis screens^6,7^. Mice engineered to test specific hypotheses may be made on variable or undefined genetic backgrounds, often without systematic or standardized multi-system phenotyping that would reveal effects not anticipated in the study design. Additionally, only ~50% of the estimated ~24,000 total protein-coding genes in the mouse currently have experimentally derived functional information available, as assessed by Gene Ontology annotation^8^. The current understanding of gene functions would be greatly enhanced by gene/phenotype data from genetically invariant mouse strains (i.e., same background strain with manipulation of only the gene(s) in question).

**Fig. 1:**
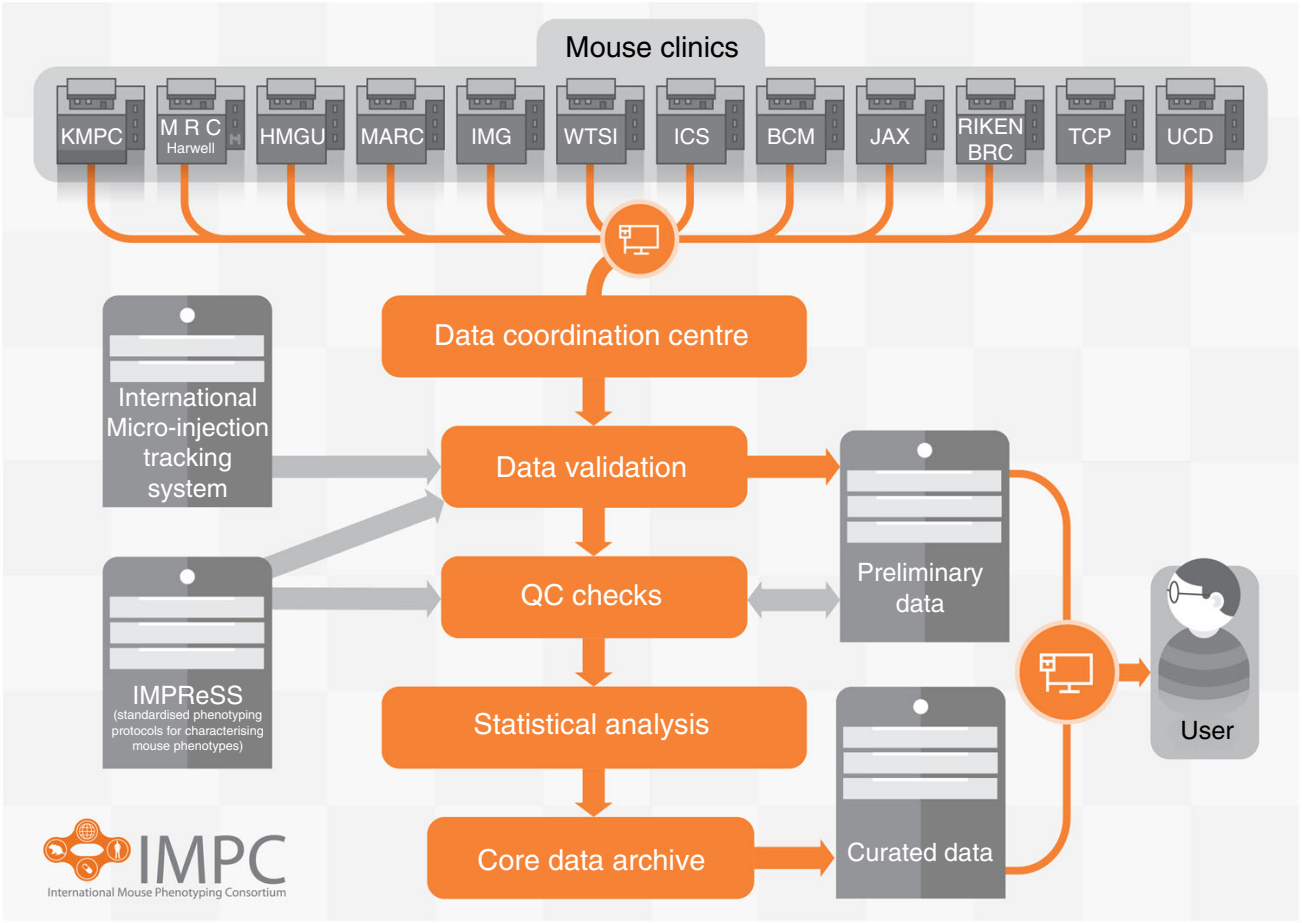
Schematic overview of IMPC data flow from acquisition to web portal availability for public users. Data are collected from 12 phenotyping centers, validated, and processed to produce curated data accessible on the project portal. Legacy data from EuroPhenome and Sanger MGP were directly transferred to the Central Data Archive at EMBL-EBI for direct integration on the portal. https://academic.oup.com/nar/article-lookup/doi/10.1093/nar/gkt977. KMPC (Korea Mouse Phenotyping Center), MRC (Medical Research Council) Harwell Institute, HMGU (Helmholtz Zentrum Muenchen), MARC (Model Animal Research Center), IMG (Institute of Molecular Genetics), WTSI (Wellcome Trust Sanger Institute), ICS (Institut Clinique de la Souris— PHENOMIN-ICS), BCM (Baylor College of Medicine), JAX (The Jackson Laboratory), RBRC (RIKEN Bio-Resource Center), TCP (The Center for Phenogenomics), UCD (University of California Davis), IMPReSS (International Mouse Phenotyping Resource of Standardized Screens https://www.mousephenotype.org/impress)

To address the fundamental problems in traditional methods of studying genetic mechanisms in cellular biology and genetic contributions to disease, the International Mouse Phenotyping Consortium (IMPC) was established in 2011 as a network of highly specialized academic centers with expertise in high-throughput mouse mutagenesis and comprehensive phenotyping^9,10^. The IMPC consists of 18 laboratories in 12 countries globally, and is supported by 5 national funding agencies including the National Institute of Health (NIH). Figure 1 and Table 1 highlight all relevant consortium partners who contribute to data production. The goal of the IMPC is to create the first functional catalog of the mammalian genome by using the proven methodology of phenotype screening of targeted gene mutagenesis in mice, which has been successful in identifying novel pathologic loci across a wide range of organ systems^11–15^. The large-scale production and characterization of the mouse genome through single-gene deletion of all protein-coding genes using multiple gene targeting strategies on a uniform C57BL/6N genetic background is currently underway^9,11,14,15^.

**Table 1:** Ocular phenotyping protocols across all IMPC phenotyping mouse clinics

Primary screen						Secondary screen
Center	Mice	Controls	Slit lamp	Indirect	Other	Additional modality
*KMPC*	7M, 7F	B6N	Yes	Yes	-	-
*MRC*	7M, 7F	B6N	Yes	Yes	-	Fundus Imaging, OCT
*HMGU*	7M, 7F	B6N	Yes	Yes	-	OCT, LIB, VDT, Scheimpflug
*MARC*	7M, 7F	B6N	Yes	Yes	-	-
*IMG*	7M, 7F	B6N	Yes	Yes	-	-
*WTSI*	7M, 7F	B6N	Yes	Yes	-	-
*ICS*	7M, 7F	B6N	Yes	No	OCT	-
*BCM*	7M, 7F	B6N	No	No	OCT	-
*JAX*	8M, 8F	B6NJ	Yes	Yes	-	Fundus Imaging, ERG
*RIKEN BRC*	7M, 7F	B6N	Yes	Yes	-	-
*TCP*	7M, 7F	B6N	Yes	Yes	Histology	-
*UCD*	7M, 7F	B6N	Yes	Yes	Histology, tonometry	Fundus Imaging, TEM, OCT, ERG

Slit-lamp examination was performed at all but one mouse clinic, and indirect fundus examination was performed at all but two mouse clinics, where routine OCT cross-sectional and funduscopic imaging was performed. Secondary screening varied across structures and was based on desired testing and available equipment for further evaluation of a suspected or confirmed phenotype See Fig. 1 legend for definitions of Center abbreviations

*ERG* (electroretinography), *TEM* (transmission electron microscopy), *OCT* (optical coherence tomography), *LIB* (laser interference biometry), *VDT* (virtual drum test)

To date, the IMPC has generated over 7000 genotype confirmed mutant strains, and has completed standardized phenotyping across 11 organ systems for 4364 of these genes. Access to mouse resources and all phenotype data are publically available at http://www.mousephenotype.org. Homozygous mice from all viable strains and heterozygous mice from all subviable or lethal strains have been subjected to ophthalmic examinations to identify ocular phenotypes, which together with their associated genotypes are presented here. Many novel genes previously unknown to be involved with mammalian ophthalmic diseases are presented, demonstrating successful identification of mouse mutants with early and delayed onset ocular phenotypes. This report sheds new light on gene function, generates new models for inherited eye diseases, and provides a roadmap for discovery of relevant monogenic and complex human disorders. The numerous novel pathologic loci revealed here serve as a powerful resource for human ocular geneticists to scan whole genome sequencing data in patients with presumed hereditary ocular disease who do not have common mutations of known disease genes. Finally, the murine loci identified in this manuscript, if validated in human patient populations, would greatly increase the number of known ocular disease genes found over the past three decades since the discovery of rhodopsin gene mutations in families with retinitis pigmentosa^16,17^.

## Results

### Primary phenotyping

The initial IMPC dataset search returned 4364 genes with completed phenotyping, each of which attained a *p*-value threshold of 10^−4^ used to assign mutant phenotypes. From the 4364 genes, a list of 347 genes with ocular phenotypes was curated. Literature searches identified 42 genetic phenotypes known to exist in vertebrates (Known Phenotype), 44 genes described to cause an ocular phenotype differing from the phenotype described in the present study (Novel Phenotype), and most interestingly, 261 genes (75% of genes with ocular phenotypes) with no prior ocular implication (Novel Gene). The number of genes within each of the three categories is shown in Table 2. Anatomical abnormalities affecting several structures or affecting the entire eye were categorized into groups: anterior segment (any combination of adnexa, cornea, iris, or lens), posterior segment (any combination of vitreous, retina, choroid, posterior sclera, or optic nerve), and whole eye (any combination across different ocular segments, e.g., retina and cornea, or eye size and vitreous, etc.). Complete phenotype results, and complete information on gene information and analysis are provided in Supplementary Table 1. Selected ocular phenotypes are presented below as exemplars.

**Table 2:** Tabular depiction of genes found to have ocular phenotypes arranged by the ocular tissue involved and the novelty of the gene or phenotype

Ocular tissue	Categorical genes (total)	Known gene (total)	Novel phenotype (total)	Novel gene (total)
Adnexa	8 (20)	0 (3)	0 (1)	8 (16)
Cornea	25 (46)	1 (6)	4 (4)	20 (36)
Iris	5 (19)	0 (6)	0 (1)	5 (12)
Lens	79 (113)	6 (13)	10 (13)	63 (87)
Vitreous	22 (40)	2 (4)	3 (4)	17 (32)
Retina	102 (139)	17 (24)	16 (20)	69 (95)
Optic nerve	4 (8)	0 (2)	0 (0)	4 (6)
Eye size	11 (18)	1 (1)	3 (4)	7 (13)
Neuro	13 (20)	0 (2)	1 (3)	12 (15)
AS Combo	24	5	3	16
PS Combo	6	1	1	4
Whole Eye	48	9	3	36
Total	347	42	44	261

A total of 347 different genes were identified. Of these, 42 genes had phenotypes that have been previously described, 44 genes had phenotypes that differed from previously described ocular phenotypes, and 261 genes previously not known to cause ocular disease were found to have ocular phenotypes. AS Combo represents combination of multiple anterior segment structures (i.e., adnexa, cornea, iris, or lens), PS Combo represents combination of multiple posterior segment structures (i.e., vitreous, retina, or optic nerve), and Whole Eye represents phenotype spanning multiple ocular segments (e.g., anterior and posterior). Neuro represents an abnormality of the pupillary light reflex. In parentheses, the total number of genes affecting a given ocular tissue is shown including genes affecting multiple ocular tissues

### Corneal phenotypes

**Fig. 2:**
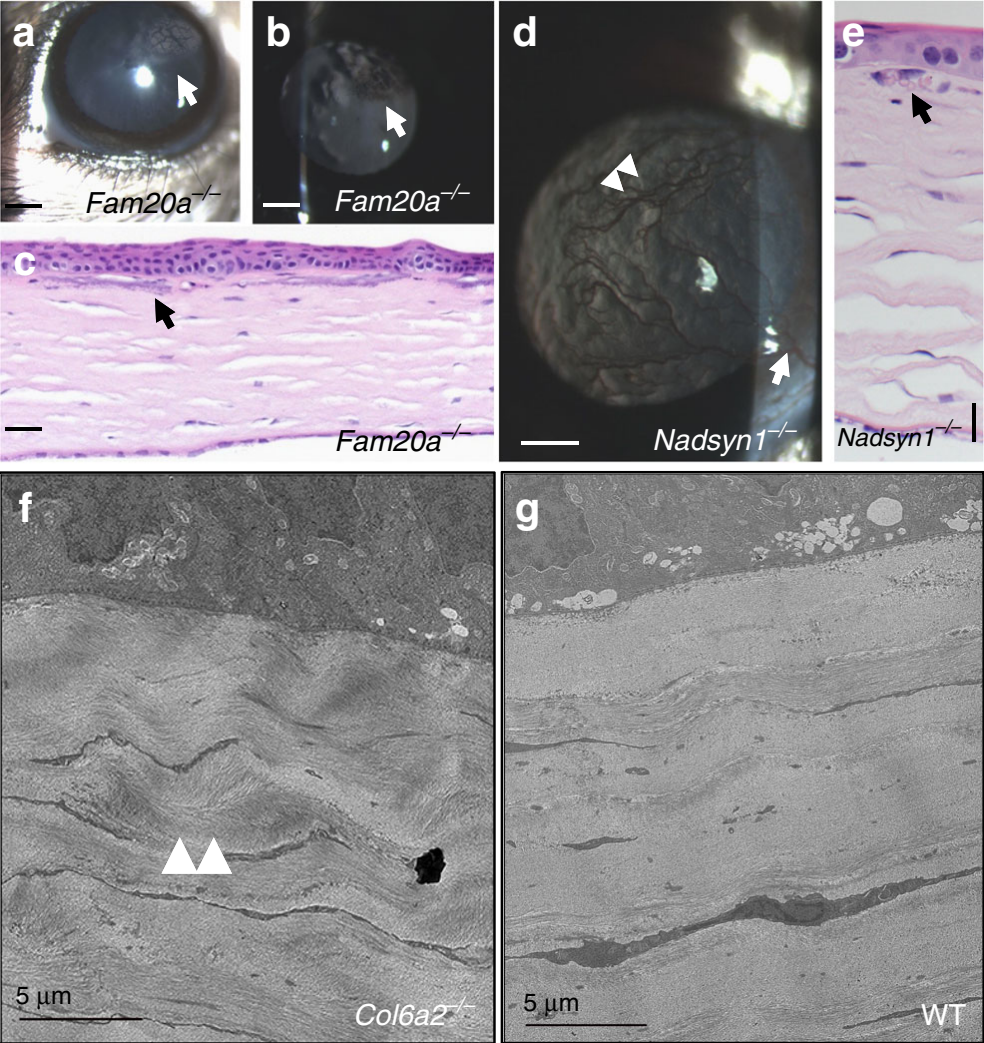
Corneal abnormalities in *Fam20a*, *Col6a2*, and *Nadsyn1* knockout mice. Biomicroscopy (**a**) of *Fam20a* knockout mice revealed polygonal opacities with indistinct edges and interweaving clear spaces in the corneal stroma (arrow), which were also apparent (arrow) on retro-illumination (**b**), scale bars = 500 µm. Histology (**c**) shows superficial stromal mineralization (arrow, scale bar = 50 µm). Corneal vascularization (arrow) and chronic superficial keratitis (arrowheads) were observed in *Nadsyn1* knockout mice (**d**, scale bar = 500 µm), and red blood cells (arrow) are shown in the lumen of neovascular vessels on histology (**e**, scale bar = 20 µm). Mice lacking *Col6a2* had subtle corneal stromal opacities seen on slit-lamp examination, which electron microscopy revealed to be a basket weaving appearance (arrowheads) of the corneal stroma (**f**) that was not seen in wild type (WT) controls (**g**), scale bars = 5 µm

A total of 25 knockout strains were found to have only corneal opacities, 20 of which were completely novel and unknown to be involved in the development of ocular phenotypes (Table 2). An additional 21 genes were identified with multifocal ocular abnormalities that included corneal phenotypes, and may represent candidates for anterior segment dysgenesis syndromes, Peters’ anomaly, and other conditions of dysgenesis. For example, *Fam20a* (Family with sequence similarity 20, member A) knockout mice exhibited large paraxial corneal opacities in a polygonal pattern, clearly visible on biomicroscopy (Fig. 2a) and retro-illumination (Fig. 2b). The corneal opacities corresponded histologically to superficial stromal mineralization (Fig. 2c). *FAM20A* deficiency has been linked to abnormal biomineralization and is associated with enamel-renal syndrome in humans^18^. Affected individuals suffer from amelogenesis imperfecta and nephrocalcinosis and/or nephrolithiasis^18,19^. *Fam20a* knockout mice have been previously described, but no corneal phenotype was reported^20^. *NADSYN1* (NAD synthetase 1) is implicated in vitamin D metabolism in humans. *Nadsyn1*-deficient mice develop chronic keratitis with neovascularization (Fig. 2d), observable by histology (Fig. 2e). Mice deficient in *Col6a2* (collagen type VI alpha 2) had very subtle corneal opacities visible to the examiner, which was determined to be due to a disorganized basket-weave appearance of the corneal stroma (Fig. 2f) by electron microscopy, rather than the typical lamellar structure of this tissue (Fig. 2g) evident in wild type control animals. The *Col6a2* gene is known to be expressed in the central corneal stroma of humans, but the phenotype of the cornea deficient in this protein has not been reported^21^.

### Lens phenotypes

**Fig. 3:**
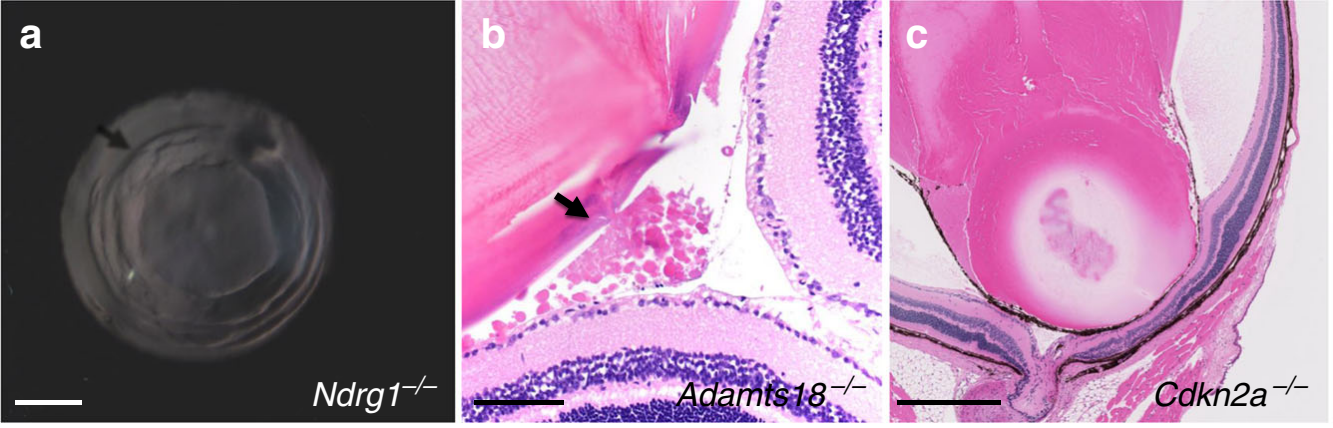
Lenticular abnormalities in *Ndrg1*, *Adamts18*, and *Cdkn2a* knockout mice. **a** Retro-illumination highlights the well-defined concentric annular anterior and posterior cortical optical discontinuities in *Ndgr1* knockout mice (arrow, scale bar = 500 µm). **b** Mice deficient in *Adamts18* knockout mice had clinically evident vitreous crystalline deposits, which represented extruded lens material (arrow, scale bar = 100 µm). **c** *Cdkn2a* knockout mice had ocular lesions consistent with persistent tunica vasculosa lentis, with most severe cases having posterior lenticonus where the lens was adhered to the retina at the optic disc. The posterior lens capsule was segmentally disrupted and there was posterior subcapsular cataract. Additionally, the retinal segment was focally dysplastic. Scale bar = 500 µm

Cataracts are another important public health concern^1^, and 79 strains bearing different single-gene deletions were identified to only have lenticular abnormalities (113 genes when considering multifocal ocular phenotypes) (Table 2). In total, 63 of these genes with lens-only phenotypes were totally novel, including *Ndrg1* and *Adamts18*. *Ndrg1* (N-myc downstream regulated gene 1) is implicated in pronephros development and in cancer biology^22,23^. The *Ndrg1* gene is a member of the N-myc downregulated gene family and encodes a protein involved in stress hormone responses as well as cell growth and differentiation. In humans, it has described roles in Schwann cell trafficking, microtubule dynamics and p53-mediated caspase activation and apoptosis^24–26^. It is known that *Ndrg1* is expressed in the eye, but an ocular phenotype has not been identified^27^. *Ndrg1* knockout mice had bilateral defects in optical fidelity characterized by distinct concentric rings involving the anterior and posterior lens cortex (Fig. 3a). The optical changes observed clinically were not detectable by routine histopathologic evaluation using light microscopy and were likely related to alterations in refractive index. By contrast, *Adamts18* (a disintegrin-like and metallopeptidase with thrombospondin type 1 motif 18, reprolysin type) knockout mice had exceptionally large and refractile crystalline opacities in the anterior vitreous, which were distinct in appearance from C57BL/6N background lesions (Fig. 3b). Upon histological examination, these opacities represented small extrusions of lens material following posterior lens capsular rupture (Fig. 3b). *ADAMTS18* in human disease has been associated with microcornea, ectopia lentis, and cone rod dystrophy^28,29^. *Adamts18*^−/−^ mice represent only a partially overlapping animal model for this human disorder given the differences in phenotype expression^30^. *Cdkn2a* null mice presented histopathological manifestations of persistent hyperplastic tunica vasculosa lentis/persistent hyperplastic primary vitreous (PHTVL/PHPV), which was characterized by presence of a pigmented posterior retrolental fibrovascular connective tissue adherent to the posterior lens capsule often resulting in cataracts. In the most severely affected case there was a posterior protrusion (posterior lenticonus) where the lens was adhered to the retina at the optic disc (Fig. 3c). The posterior lens capsule was segmentally disrupted and there was a globular degeneration (Morgagnian globules) of the subcapsular lens protein (posterior subcapsular cataract). The retinal segment was focally jumbled (dysplastic) in this case. CDKN2A in human disease has been linked to suppression of neoplastic growth, and knockout mice have shown to develop early spontaneous tumors but no ocular phenotypes^31^. However, another report showed that suppression of *Cdkn2a* resulted in cataract development with failed regression of the hyaloid vascular system similar to human PHPV, as found in *Cdkn2a* knockout mice in the present study^32^.

### Retinal phenotypes

**Fig. 4:**
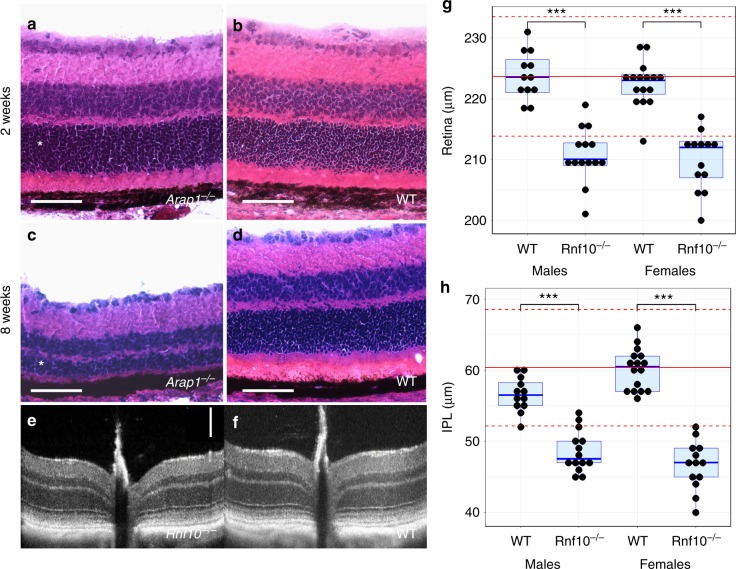
Retinal thinning in *Arap1* and *Rnf10* knockout mice. *Arap1*^−/−^ mice had normal appearing retinal tissue at 2 weeks postnatal age (**a**) in comparison to wild type (WT) littermate control animals (**b**). The outer nuclear layer (asterisks in **a**, **c**) progressively degenerated (**c**) by 8 weeks postnatal in *Arap1*^−/−^ mice, when compared with littermate control eyes (**d**). Scale bars = 50 µm. Optical coherence tomography of *Rnf10*^−/−^ mice (**e**) shows statistically significant retinal thinning by 16 weeks postnatal age when compared to control retinal images (**f**). Scale bar = 100 µm. Box plots of male and female *Rnf10*^−/−^ mice document retinal thinning (**g**) in comparison to normal age-matched controls, particularly affecting the inner plexiform layer (IPL) (**h**), and the inner nuclear layer. Error bars represent the standard deviation (SD) for the knockout measurements. Data points outside of the mean + /- SD range are represented by black circles. The two dotted red lines delimit the reference range, defined as two SD away from the mean for controls, represented by the solid red line

Retinal diseases are the foremost cause of blindness in the developed world, and studies to protect retinal neurons from degeneration are a major area of active research^33^. Ocular phenotyping in the IMPC pipeline identified 102 retina-only phenotypes (139 phenotypes when considering genes leading to multifocal ocular phenotypes), 17 of which are previously well studied and published (Table 2). A total of 69 retinal phenotypes involve genes that have no previously reported ocular phenotype, and potentially represent novel human disease alleles. *Arap1* (Arf GAP with Rho GAP domain, ankyrin repeat, and PH domain 1) is a large and complex soluble GTPase, which regulates membrane biomechanics and lysosome maturation, but the extent of its functions is not well understood^34^. This gene plays a role in the recycling of the EGF receptor to the plasma membrane, potentiating this signaling pathway^35,36^. Subsequent studies following primary phenotyping document progressive photoreceptor degeneration in *Arap1*^−/−^ mice, similar to retinitis pigmentosa in humans^37^. Furthermore, *Arap1* appears to be expressed in Müller glial and RPE cells, not in photoreceptors themselves, implicating a specific and novel dependence of photoreceptors on *Arap1* expression in neighboring cells^38^. *Arap1*^−/−^ animals developed a histologically normal retina by 2 weeks postnatal age (Fig. 4a), similar to that of control mice (Fig. 4b). By 8 weeks postnatal, however, the *Arap1*^−/−^ outer nuclear layer degenerated substantially (Fig. 4c), unlike the age-matched control retinas (Fig. 4d). Our phenotyping efforts have also yielded more subtle examples of retinal thinning, such as the *Rnf10*^−/−^ mouse (Fig. 4e), in which retinal thickness was lower compared to control mice (Fig. 4f). Quantitation of retinal histology demonstrates the global retinal thinning in these mutants, particularly in the inner nuclear layer and inner plexiform layer (Fig. 4g): when comparing to the control group examined in parallel, WT and *Rnf10*^−/−^ retinal thicknesses were respectively of 223.8 ± 3.9 µm (*n* = 12 eyes) vs. 210.6 ± 4.5 µm (*n* = 14 eyes) for males (*p* = 4.10^−8^), of 222.4 ± 3.8 µm (*n* = 16 eyes) vs. 209.8 ± 4.8 µm (*n* = 13 eyes) for females (*p* = 2.10^−8^). The inner nuclear layer thickness was 27.8 ± 2.1 µm vs. 24.7 ± 2.1 µm for WT and *Rnf10*^−/−^ males (*p* = 0.0013), 26.6 ± 2.3 µm vs. 25.0 ± 2.2 µm for WT and *Rnf10*^−/−^ females (*p* = 0.063). The inner plexiform thickness was 56.6 ± 2.4 µm vs. 48.6 ± 2.9 µm for WT and *Rnf10*^−/−^ males (*p* = 8.10^−8^), 60.1 ± 2.9 µm vs. 46.6 ± 3.4 µm for WT and *Rnf10*^−/−^ females (*p* = 6.10^−12^). In a more global comparison, 78.6% of the male (*n* = 14) and 84.6% of the female (*n* = 13) (81.5% overall) *Rnf10*^−/−^ retinas had a thickness below the reference range. While only of 21.4% and 23.1% of the mutant male and female eyes (22.2% overall) had an inner nuclear layer thickness below the reference range, these percentages were 85.7% and 100% (92.6% overall) when considering the inner plexiform values. *Rnf10*, ring finger protein 10, may have a role in transmitting NMDA receptor activity to the nucleus of hippocampal neurons, and may have transcriptional regulation capability^38,39^. Its role in the retina was previously unknown.

**Fig. 5:**
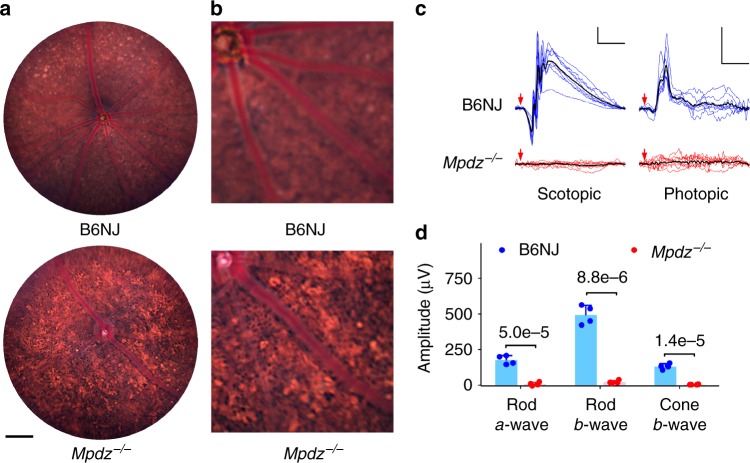
Ocular phenotypes of homozygous C57BL/6NJ-*Mpdz*^*em1J*^/J (*Mpdz*^−/−^) and control C57BL/6NJ (B6NJ) mice. Bright-field fundus images at 14 weeks of age (**a**). Detail of **a** showing enhanced detection of RPE cells (**b**) in mutant mice. Scale bar in **a** = 250 µm. ERG traces (**c**) from the left and right eyes of B6NJ (blue) and *Mpdz*^−/−^ (red) mice (*n* = 4 both strains) at 16 weeks of age. The mean of all traces is shown in *black. Scale bars*, 100µV vertical, 50 ms horizontal. Summary of ERG response amplitudes (**d**) for data in **b**. Bars show mean ± SD; *p-*values from *t*-tests are indicated

*Mpdz* (multiple PDZ domain protein) knockout mice exhibited a mottled fundus (Fig. 5a, b) characterized by irregular bright patches. The enhanced detection of retinal pigmented epithelium (RPE) cells by fundus imaging (Fig. 5b) is typical of mouse models of retinitis pigmentosa and Leber congenital amaurosis, in which the outer retina has thinned or failed to develop^40,41^. Scotopic and photopic electroretinography responses were absent at 16 weeks of age (Fig. 5c), indicating a profound defect in rod and cone photoreceptor function (Fig. 5d). MPDZ is a tight junction protein and its silencing disrupts epithelial cell barrier function *in vitro*^42,43^. The protein has been localized to the mouse retinal external limiting membrane (ELM) and RPE, thus potentially compromising ELM or RPE barrier function in the knockout strain^44,45^. *Mpdz* variants are associated with hydrocephalus, foveal dysplasia, and inner retinal thinning in humans. Detailed analysis of mouse ocular phenotypes are consistent with human ocular phenotpyes, and in an independent mouse knockout model, but ocular phenotypes have not been reported thus far^43,46–48^.

### Gene enrichment analysis

Using a gene ontology enrichment analysis, Wnt signaling pathways were shown to be considered important signaling pathways for all 347 genes including known phenotypes (Supplementary Figs. 1–4 and Tables 2–4). In a subset analysis of the 261 novel genes, oxidation-reduction processes (28 genes) and histone H3-K36 dimethylation pathways (3 genes) are the top-ranked functional annotations (*p*-value, 5.16E-0.5 and 4.76E-04, respectively). Cataract and lens morphology phenotypes were observed in mice with the majority of genes associated with oxidation-reduction process (*n* = 15/28, 54%). Oxidative stress is an important pathogenic mechanism in numerous eye diseases including age-related macular degeneration, glaucoma, or cataract^49,50^.

## Discussion

Large-scale ocular screens as part of the IMPC phenotyping pipeline of mutant mice have revealed a large number of mutants with ocular phenotypes. With deletion of 4364 genes being analyzed at the time of this writing we report 347 ocular phenotypes, 261 of which are novel and may serve as a powerful tool in future genetic research of human ocular disease. If validated in humans, the genes described here would greatly increase the number of known ocular disease genes. Genes not described here that would be suspected to have ocular phenotypes based on known human counterparts may have been lethal knockouts or may have not yet been phenotyped. Information on all tested genes can be found at www.mousephenotype.org^51^. A total of 86 knockout strains had genes deleted that are previously known to cause ocular phenotypes (44 novel phenotypes, and 42 known phenotypes), 25 of which knockout strains have not yet been created. In many cases, knockout mice for these genes have been previously reported, and in other cases the deleted gene has a known disease-causing mutation in human pedigrees, or in other vertebrate genetic models such as zebrafish. However, for these 86 known disease-causing genes, the mice phenotyped in this study may represent novel mouse models of human disease states. A total of 205 of these novel models exhibit isolated ocular findings, while 56 others recapitulate syndromic patterns affecting ocular tissues as well as other organ systems, as previously reported in mouse mutational studies^52^. These models can be used for various forms of translational research, including therapeutic testing of potential medications, gene therapy, or stem cell-based studies.

All knockout mice in this study were made on the C57BL/6N strain, which is homozygous for the *Crb1*^rd8^ mutation that is associated with retinal dysplasia^53,54^, and depending on the strain background, slow retinal degeneration^55,56^. These mice also exhibit other ocular abnormalities associated with the strain genetic background, occurring at different frequencies, including corneal deposits, altered anterior lens capsular translucency, punctate nuclear cataracts, and vitreous crystalline and/or pigmentary opacities^57^. It should be acknowledged that a very small portion of the genes presented here may be disease-modifying genes, and may cause only a mild or no detectable ocular phenotype, and/or a delay in phenotype appearance, when knocked out in the context of other strains not carrying the *Crb1*^*rd8*^ alleles. Additionally, because mice were only examined up to 16 weeks of age, late-onset phenotypes may have been overlooked. However, it is important to note that the intent of the IMPC project is to do a first pass screen to generate hypotheses and assign a functional annotation to the vast majority of genes for which there is little to no knowledge of biological function or pathologic role. Specific genes can then be explored further, including through elaboration of functions not assessed in the IMPC pipeline, where additional important pathophysiologic information about the disease mechanism underlying the observed pathology may be obtained. Select knockout mouse strains have been chosen for advanced analysis in separate studies by some of the authors of this manuscript, and in some instances, the knockouts have been bred away from the *Crb1*^rd8^ mutation^40^.

Ophthalmic diseases with a genetic basis can be part of a multi-system syndrome, or they can be caused by either single- or multi-gene disorders limited to the eye. In general, multi-system hereditary conditions that affect the eye are relatively uncommon and make up only a small segment of ocular disease. The IMPC project has the potential to identify genetic disorders that affect more than just the eye. Through comprehensive phenotyping, associations between ocular and systemic abnormalities that might not have been detected thus far in the human population can be discovered. For example, observing that a given knockout mouse with kidney abnormalities has a concomitant retinal degeneration may motivate genetic researchers to evaluate this gene as a potential oculo-renal syndrome or syndromic cilliopathies, of which causation by several single-gene mutations are known^58–60^. Knockouts of several genes described in this study with previously unreported ocular phenotypes were found to have coexisting kidney disease: *Aqp6*, *Dnase1l2*, *Efna5*, *Fgf7*, and *Galk2*. Comprehensive phenotyping of all organ systems as performed by the IMPC may provide many other multi-systemic disease associations and useful insights for understanding and treating human disease, including but not limited to identifying pathways involed in disease progression, identification of biomarker candidates for disease analysis, and surrogate measures for therapeutic testing.

The majority of ocular diseases show strong Mendelian inheritance patterns suggestive of single-gene causation. However, multi-gene disorders, resulting from several single-nucleotide polymorphisms in various genes that ultimately contribute to the observed phenotype, exist and are often associated with aging as commonly seen in age-related macular degeneration, diabetic ocular disease, and some forms of cataract, glaucoma, and corneal disease^1,61,62^. In recent years, genome-wide association studies have revealed surprising and important genetic risk alleles for ocular diseases, and the discovery of single-gene mutations/deletions causing ocular disease, as provided in this study, may be relevant in a host of multi-factorial diseases afflicting the aging population^61–64^. More immediately impactful in clinical ophthalmology are the single-gene disorders with Mendelian patterns of inheritance as reported in the present study. Not only can the novel genes reported here be used to develop allele-specific models for translational research of human diseases, which can form the foundation for future human trials, they may rapidly impact diagnostics in clinical ophthamology. For example, when a patient presents to an ophthalmologist with ocular disease that is suspected to be influenced by heritability, blood can be tested relatively inexpensively and efficiently screened using genetic arrays to test for common mutations of known genes^65^. If no commonly known genetic mutations are identified on the initial genetic array, the decision can be made to invest in whole exome or whole genome sequencing. Once the expanded genomic data are available for the patient, the coding sequence for known disease genes can be analyzed for uncommon mutations not on the array, or for novel unpublished mutations in known genes.

This strategy has been successful in identifying the disease-causing mutation in 15–35% of patients with hereditary eye disease^66^. The clinician-geneticist is then faced with the bioinformatics problem of isolating the single mutated gene within the patient’s entire genome sequencing data. Interrogation of the genome using next generation sequencing can detect 40–55% of mutations^67,68^. Finding the remaining half of the mutations is an incredibly challenging and time consuming problem when the geneticist has run out of candidate genes to examine, and is forced to examine the remainder of coding and non-coding sequences in an unbiased fashion. This is where identifying novel disease-causing mutations through animal screens represents a powerful tool. The 256 completely novel genes and 48 genes reported here with novel phenotypes (302 total genes) approximate the known number of eye disease-causing loci in mammals, and may help direct human geneticists to candidate genes that will likely be relevant in human ocular disease and blindness^69^.

The identification of candidate genes tremendously increases the efficiency of such a bioinformatics challenge by guiding the queries to examine an expanded pool of potential candidate genes, and by decreasing the number of cases that must be arduously performed in an unbiased methodology. We anticipate that the list of genes identified here will guide human geneticists to the identification of relevant disease genes in humans^70^. Furthermore, the mice produced in the pipeline are available from public resources, such as the NIH-supported Mutant Mouse Resource and Research Centers (MMRRC) or the European Mouse Mutant Archive (EMMA). These animals are immediately available and hold the potential for being validated as relevant models of human ocular diseases. Once validated, they can be used to accelerate identification and development of lead therapeutic compounds employing small molecules, biologics, gene therapies, and cell-based treatments. Finally, many of the genes reported here have not previously been recognized to play a role in the visual system, establishing an expanded menu of potential therapeutic targets once signaling pathways are established.

## Methods

### Animals

All IMPC centers maintain strict ethical review licensing and accrediting bodies that are reflective of their national legislation (Institutional Animal Care and Usage Committees, Regierung von Oberbayern, Com’Eth, Animal Welfare and Ethical Review Bodies, RIKEN Tsukuba Animal Experiments Committee, and Animal Care Committee). Phenotyping procedures consortium-wide were assessed routinely for animal welfare and refined where applicable to minimize suffering. Electroretinograms were performed in complicance with the Association for Research in Vision and Ophthalmology statement for the Use of Animals in Ophthalmic and Vision Research.

All mice are IMPC generated knockout lines using principally the International Knockout Mouse Consortium t*m1.1* and *tm1b* null alleles, but also including a small number of CRISPR/Cas9-induced mutations that employ an exon deletion stragegy^9^. Homozygous viable mutants enter an extensive adult phenotyping pipeline that includes a range of phenotyping tests that evaluate a diverse range of physiological systems, including the visual sensory system.

Cohorts of a minimum of seven male and seven female adult mice, homozygous for a disrupted allele on a C57BL/6N genetic background, along with two male and two female wild-type littermates per genetic knockout, were produced. Approximately 24% of the strains were homozygous embryonic lethal or subviable, necessitating adult phenotyping of adult heterozygous mice. Morphological imaging and other analyses of embryos harvested at various stages of development was carried out in order to determine the time and likely cause of death^12^. Phenotyping began at 4 weeks of age, followed by weekly body weights, and more in-depth phenotyping from 9 through 16 weeks of age. Mice were euthanized at 16 weeks and tissues and blood were collected for anatomic and clinical pathology. Testing and data collection were performed using standardized operating protocols shared among IMPC centers, with sufficient flexibility for each phenotyping center to explore additional secondary phenotype tests while maintaining rigorous principles of scientific consistency and reproducibility^71^. At all sites, phenotype assessment and interpretation were made by specialized personnel with expertise in a variety of fields, including pathologists, veterinarians, radiologists, ophthalmologists, cardiologists, and scientific experts in neurobehavior, metabolism, cardiovascular physiology, musculoskeletal anatomy, and sensory nervous systems.

Available data and annotated images are viewable and accessible for download online at www.mousephenotype.org^51^. The IMPC uses a rigorous and structured pipeline for collecting data (Fig. 1). Local phenotyping centers collect data in their Laboratory Information Management Systems based on the IMPReSS protocols (International Mouse Phenotyping Resource of Standardized Screens https://www.mousephenotype.org/impress) where the parameters and metadata types are strictly defined. The centers then collate their experimental data and publish XML (Extensible Markup Language) documents as defined by the IMPReSS protocols and the IMPC designed Schema Definition Language. The documents are stored on a local Secure File Transport Protocol server until the Data Coordination Center at MRC Harwell can download them for processing, validation, and quality control (QC). The Data Coordination Center then checks the data for completeness and QC, and also compares the data against the production information in the International Micro-Injection Tracking System. Some preliminary data analyses are employed to detect subtle QC issues that may indicate process or equipment problems. Data that passes the final QC are sent to the Core Data Archive where statistical analysis is performed by the PhenStat program to identify statistically validated phenotypes^72^. At that point, the curated data are made available to the public at the IMPC (http://www.mousephenotype.org/).

### Primary phenotyping

Complete ophthalmic examinations were performed on both eyes of each mouse at 15–16 weeks of age. A standardized operating protocol for evaluation of ocular and adnexal structures was followed by all study sites (https://www.mousephenotype.org/impress/protocol/267/7). Biannual meetings of consortium participants were held to troubleshoot the ocular phenotyping protocol in order to maintain high-quality data management. Examinations were carried out by highly trained and experienced technical support staff trained to identify and differentiate background lesions common in the C57BL/6N strain^57^. Examiners were overseen by lead site scientists who subsequently reviewed all phenotypes. Specific phenotyping modalities utilized at each site are summarized in Table 1.

Examiners were unaware of knockout and wild-type status and of the specific genetic deletion during examinations. Each cohort of knockout mice was examined with genetic wild-type controls of both sexes mixed into the cohort. If ocular phenotypes were discovered in a cohort of mice, they were considered to be due to the knockout genotype only if they were identified in the knockouts and not in the controls. This process reduced the chance for false positives being identified as examiners were masked to a mouse’s genotype. Pupillary light reflexes were evaluated, the eyelids, third eyelid, conjunctiva, sclera, cornea, iris, and anterior chamber were examined using broad beam illumination at the highest intensity setting (Kowa SL-15, Kowa, Tokyo, Japan, or equivalent) with magnification set at 16 × . The irides of all mice were then pharmacologically dilated with a solution of 1:7 10% phenylephrine HCl (Akorn Inc., Lake Forest, IL, USA, or equivalent): 1% tropicamide (Bausch & Lomb Inc., Tampa, FL, USA, or equivalent). A 0.1 mm slit beam at the highest intensity setting to evaluate the anterior segment (cornea, anterior chamber, and lens), followed by posterior segment evaluation including the vitreous chamber. Fundus examinations were performed via indirect ophthalmoscopy using a 60 Diopter double aspheric handheld lens (Volk Optical Inc, Mentor, OH, USA or equivalent) and a portable indirect headset (Keeler AllPupil II LED Vantage Plus Wireless Headset, Keeler Instruments Inc., Broomall, PA, USA, or equivalent).

Acquired incidental (i.e., trauma) findings were identified based on lesion characteristics and lack of repeatability in a given cohort. Background lesions were identified based on expected changes associated with the strain-specific C57BL/6N and retinal degeneration 8 (*rd8*) mutations in *Crb1*^53^. Findings in both categories were excluded from reported results. Background findings known for the C57BL/6N line include corneal stromal opacities, altered anterior lens capsule translucency, pulverulent nuclear cataracts, vitreous pigment and crystalline opacities, retinal dysplasia, and microphthalmia^57^. Except in knockout strains that greatly increased the frequency of these specific lesions, background lesions occurred at approximately equal frequencies among knockout and wild-type lines as previously described^57^.

### Histology

Physiologic data were supported by histopathology, which continues to be the definitive assay in medicine for making most diagnoses^73,74^. For pipeline histopathologic evaluation, eyes were either enucleated or fixed in situ. Tissues were immersion fixed in 10% neutral-buffered formalin. If fixed in situ, formalin-fixed heads were decalcified in 15% picric acid for at least 24 h or until sufficient decalcification was achieved. Parasagittal sections of eyes or coronal sections through the head including eyes were processed routinely for histopathology, embedded in paraffin, sectioned at 4–5 µm, and stained with hematoxylin and eosin.

For targeted anterior segment histology, eyes were enucleated, fixed in 10% neutral-buffered formalin, washed in three changes of 100% ethanol for 15 min each, two 15 min changes of 100% xylene, and three 45 min changes of 60C paraffin. Sections 4 µm thick were cut using a Leica microtome and de-paraffinized prior to staining. For targeted evaluation of the retina, eyes were enucleated and frozen in Optical Cutting Temperature Compound (Fisher HealthCare, Houston, TX, USA) using liquid nitrogen. Cryosections were prepared at 10 µm thickness on Superfrost Plus slides (Thermo Fisher Scientific Inc., Waltham, MA, USA). Sections were treated with cold acetone, hematoxylin, 0.5% HCl in 70% ethanol, eosin, 95% and 100% ethanol, and Histo-Clear (National Diagnostics, Atlanta, GA). Slides were coverslipped with Vectamount and imaged. The histologic findings were evaluated by an anatomic pathologist (in all specific examples presented in the results, by a board-certified veterinary anatomic pathologist: D.M.I., C.R.).

### Electron microscopy

Selected cases were examined by transmission electron microscopy (TEM). Eyes were immediately placed in 4% glutaraldehyde and 2% PFA in PBS for 2 + h at 4 °C, thoroughly washed with PBS, and cut into squares. Small pieces of tissue were fixed in 1% osmium tetroxide in 0.1 M phosphate buffer (pH 7.3) for 1 h. After buffer rinses, the pieces were dehydrated in a graded series of ethanol, incubated in propylene oxide, then infiltrated and embedded with epoxy resin (Poly/Bed 812; Polysciences Inc.). Initial sections of tissue were obtained by bright-field microscopy, and thin sections were cut from selected areas, stained with uranyl acetate and lead citrate, and examined with a Philips CM120 transmission electron microscope.

### Ocular imaging

All mice diagnosed with ocular lesions suspected to be a genetic phenotype were flagged for further examination via advanced imaging techniques. Mice were anesthetized with an intraperitoneal injection of a ketamine/midazolam (50–75/1–2 mg/kg) cocktail. Eyes were dilated with tropicamide 1% and phenylephrine 2.5% drops, and lubricated with methylcellulose containing artificial tears. Slit-lamp anterior segment photographs were taken with a BQ900 slit lamp (Haag-Streit, Switzerland). Fundus images were obtained with the Micron III or IV (Phoenix Research Laboratories, Pleasanton, CA, USA). Video acquisitions of 100 frames were registered, averaged, and sharpened digitally as described^75^. Spectral domain OCT images were captured with the Bioptigen Envisu R2200 (Leica Microsystems, Wetzlar, Germany). Retinal and layer thickness are indicated as mean ± standard deviation (SD) and were comparing using a Student t-test to the control group examined in parallel. For a given type of measurement, the reference range is defined as the mean ± 2 SD of all control measurements.

### Electroretinography

Electroretinography (ERG) studies were conducted at institutions where available (see Table 1), on mice at 16 weeks of age for each knockout line (two males, two females) and, on a weekly basis, C57BL6/NJ (JAX stock #005304) control mice (two males, two females). An Espion^2^ Electroretinography System (Diagnosys, LLC, Lowell, MA) was used to assess flash-induced voltage changes in both eyes of animals. Mice were either dark-adapted for 2 h prior to exam and stimulated by five flashes at an illuminance of 0.25 cd·s/m^2^ and a frequency of 0.1 Hz (scotopic condition), or light-adapted for 8 min with a 110 cd/m^2^ background illumination and additionally stimulated on this background with 20 flashes at an illuminance of 10 cd·s/m^2^ and a frequency of 1 Hz (photopic condition). The color temperature of all stimuli and background illumination was 6500 K. Flashes under each condition were averaged and amplitudes determined using the Espion^2^ software. ERG response amplitudes from the left and right eye of each mouse were averaged, and the mean values from the mutant and control cohorts were analyzed statistically in Prism (GraphPad Software, Inc., La Jolla, CA) using Student’s *t-*test. ERG trace data from each eye was exported from the Espion^2^ software and graphed in Prism.

### Gene analysis

The initial list of eye phenotypes was developed by searching the IMPC web portal for non-specific eye phenotypes in an effort to obtain all relevant knockouts with ocular phenotypes (http://www.mousephenotype.org/data/search/mp?kw=eye). Phenotypes only recognized at gross pathology or histopathology stages were excluded, as were embryologic eye phenotypes, since IMPC statistical thresholds were not applied to phenotypes discovered by these methods. The initial list was analyzed to identify unique genes with ocular phenotypes. The list of knockouts was then categorized based on the region of the eye in which the phenotype was detected. Genes associated with an ocular phenotype were then reviewed by searching www.pubmed.gov and www.google.com/scholar for each unique gene and the search term eye, the affected anatomical structure (e.g., cornea), and the term knockout to determine if a genetic knockout has been made. If the search yielded an example of at least one vertebrate species with a published ocular phenotype that was similar to the phenotype found in the present study, the genetic phenotype was categorized as a Known Phenotype. If the search yielded evidence of an ocular phenotype for a given genetic knockout, but the phenotype was of a different ocular tissue (e.g., cornea vs. retina), then the genetic phenotype was called Novel Phenotype. Knockouts in the Novel Phenotype category are still considered novel mouse models of ocular disease. If the search yielded no publications on ocular phenotypes of a given gene, the genetic phenotype was categorized as Novel Gene.

### Gene enrichment analysis

We used the DAVID functional annotation tool (version 6.8) to examine whether particular gene ontology (GO) terms of biological processes are enriched in a list of genes associated with the eye phenotype (combined, known, or novel)^76^. All GO terms of GOTERM_BP_FAT with modified Fisher exact *p*-values <0.05 were plotted with ReviGO with default options^77^. We manually classified GO terms into representative GO groups in the ReviGo interactive graph. To reconstruct a network model of novel genes associated with the eye phenotype, we collected their experimentally validated protein–protein interactions from five protein–protein interactome databases: BioGRID (version 3.4.147)^78^, HitPredict (version 4)^79^, IntAct (version 2017-03-02)^80^, DIP (version 2017-02-05), and MINT (version)^81^. We also collected predicted protein–protein interactions for the novel genes from STRING (version 10.5)^82^. The network model was built with the novel genes and their combined protein–protein interactions using Cytoscape (version 3.5.1)^83^. We then overlapped the eye location of the novel genes and their membership of the representative GO groups in the constructed network model.

### Disclaimer

The content is solely the responsibility of the authors and does not necessarily represent the official views of the National Institutes of Health

## Electronic supplementary material

Supplementary Information

Description of Additional Supplementary Files

Supplementary Data 1

## Data Availability

Data are available from the IMPC at www.mousephenotype.org. Post-QC data from the Core Data Archive (http://www.mousephenotype.org/ data/documentation/data-access) is available in the form of RESTful interfaces. Pre-QC data are available upon request (http://www.mousephenotype.org/contact-us) or by manual download (Phenoview).
