# Author Correction: Identification of genes required for eye development by high-throughput screening of mouse knockouts

**DOI:** 10.1038/s42003-019-0349-y

**Published:** 2019-03-07

**Authors:** Bret A. Moore, Brian C. Leonard, Lionel Sebbag, Sydney G. Edwards, Ann Cooper, Denise M. Imai, Ewan Straiton, Luis Santos, Christopher Reilly, Stephen M. Griffey, Lynette Bower, David Clary, Jeremy Mason, Michel J. Roux, Hamid Meziane, Yann Herault, International Mouse Phenotyping Consortium, Anna Swan, Ruairidh King, Piia Keskivali-Bond, Lois Kelsey, Igor Vukobradovic, Dawei Qu, Ruolin Guo, Elisa Tran, Lily Morikawa, Milan Ganguly, Napoleon Law, Xueyuan Shang, Patricia Feugas, Yanchun Wang, Yingchun Zhu, Kyle Duffin, Ayexa Ramirez, Patricia Penton, Valerie Laurin, Shannon Clarke, Qing Lan, Gillian Sleep, Amie Creighton, Elsa Jacob, Ozge Danisment, Joanna Joeng, Marina Gertsenstein, Monica Pereira, Sue MacMaster, Sandra Tondat, Tracy Carroll, Jorge Cabezas, Amit Patel, Jane Hunter, Gregory Clark, Mohammed Bubshait, David Miller, Khondoker Sohel, Alexandr Bezginov, Matthew McKay, Kevin Peterson, Leslie Goodwin, Rachel Urban, Susan Kales, Rob Hallett, Dong Nguyen-Bresinsky, Timothy Leach, Audrie Seluke, Sara Perkins, Amanda Slater, Rick Bedigian, Leah Rae Donahue, Robert Taft, James Denegre, Zachery Seavey, Amelia Willett, Lindsay Bates, Leslie Haynes, Julie Creed, Catherine Witmeyer, Willson Roper, James Clark, Pamela Stanley, Samantha Burrill, Jennifer Ryan, Yuichi Obata, Masaru Tamura, Hideki Kaneda, Tamio Furuse, Kimio Kobayashi, Ikuo Miura, Ikuko Yamada, Hiroshi Masuya, Nobuhiko Tanaka, Shinya Ayabe, Atsushi Yoshiki, Valerie Vancollie, Francesco Chiani, Chiara Di Pietro, Gianfranco Di Segni, Olga Ermakova, Filomena Ferrara, Paolo Fruscoloni, Alessia Gambadoro, Serena Gastaldi, Elisabetta Golini, Gina La Sala, Silvia Mandillo, Daniela Marazziti, Marzia Massimi, Rafaele Matteoni, Tiziana Orsini, Miriam Pasquini, Marcello Raspa, Aline Rauch, Gianfranco Rossi, Nicoletta Rossi, Sabrina Putti, Ferdinando Scavizzi, Giuseppe D. Tocchini-Valentini, Colin McKerlie, Ann M. Flenniken, Lauryl M. J. Nutter, Zorana Berberovic, Celeste Owen, Susan Newbigging, Hibret Adissu, Mohammed Eskandarian, Chih-Wei Hsu, Sowmya Kalaga, Uchechukwu Udensi, Chinwe Asomugha, Ritu Bohat, Juan J. Gallegos, John R. Seavitt, Jason D. Heaney, Arthur L. Beaudet, Mary E. Dickinson, Monica J. Justice, Vivek Philip, Vivek Kumar, Karen L. Svenson, Robert E. Braun, Sara Wells, Heather Cater, Michelle Stewart, Sharon Clementson-Mobbs, Russell Joynson, Xiang Gao, Tomohiro Suzuki, Shigeharu Wakana, Damian Smedley, J. K. Seong, Glauco Tocchini-Valentini, Mark Moore, Colin Fletcher, Natasha Karp, Ramiro Ramirez-Solis, Jacqueline K. White, Martin Hrabe de Angelis, Wolfgang Wurst, Sara M. Thomasy, Paul Flicek, Helen Parkinson, Steve D. M. Brown, Terrence F. Meehan, Patsy M. Nishina, Stephen A. Murray, Mark P. Krebs, Ann-Marie Mallon, K. C. Kent Lloyd, Christopher J. Murphy, Ala Moshiri

**Affiliations:** 1 William R. Pritchard Veterinary Medical Teaching Hospital, School of Veterinary Medicine, University of California-Davis, Davis, 95616 CA USA; 2 Department of Surgical and Radiological Sciences, School of Veterinary Medicine, University of California-Davis, Davis, CA 95616 USA; 3 Comparative Pathology Laboratory, School of Veterinary Medicine, University of California-Davis, Davis, CA 95616 USA; 4 Medical Research Council Harwell Institute (Mammalian Genetis Unit and Mary Lyon Center), Harwell, Oxfordshire, OX11 0RD UK; 5 Mouse Biology Program, and Department of Surgery, School of Medicine, University of California-Davis, Davis, CA 95618 USA; 6 European Molecular Biology Laboratory, European Bioinformatics Institute, Wellcome Genome Campus, Hinxton, Cambridge, CB10 1 SD UK; 7 Institut de Génétique et de Biologie Moléculaire et Cellulaire, Université de Strasbourg, 1 rue Laurent Fries, 67404 Illkirch, France; 8 Centre National de la Recherche Scientifique, UMR7104 Illkirch, France; 9 Institut National de la Santé et de la Recherche Médicale, U1258 Illkirch, France; 10 Université de Strasbourg, 1 rue Laurent Fries, 67404 Illkirch, France; 11 CELPHEDIA, PHENOMIN, Institut Clinique de la Souris (ICS), CNRS, INSERM, University of Strasbourg, 1 rue Laurent Fries, 67404 Illkirch-Graffenstaden, France; 12 The Centre for Phenogenomics, Toronto, ON M5T 3H7 Canada; 13 The Hospital for Sick Children, Toronto, ON M5G 1X8 Canada; 14 Lunenfeld-Tanenbaum Research Institute, Mount Sinai Hospital, Toronto, ON M5G 1X5 Canada; 15 Department of Molecular Physiology and Biophysics, Baylor College of Medicine, Houston, TX 77030 USA; 16 Department of Molecular and Human Genetics, Baylor College of Medicine, Houston, TX 77030 USA; 17 The Jackson Laboratory, Bar Harbor, ME 04609 USA; 18 SKL of Pharmaceutical Biotechnology and Model Animal Research Center, Collaborative Innovation Center for Genetics and Development, Nanjing Biomedical Research Institute, Nanjing University, Nanjing, 210061 China; 19 RIKEN BioResource Center, Tsukuba, Ibaraki 305-0074 Japan; 20 Clinical Pharmacology, Charterhouse Square, Barts and the London School of Medicine and Dentistry, Queen Mary University of London, London, EC1M 6BQ UK; 21 Korea Mouse Phenotyping Consortium (KMPC) and BK21 Program for Veterinary Science, Research Institute for Veterinary Science, College of Veterinary Medicine, Seoul National University, 599 Gwanangno, Gwanak-gu, Seoul, 08826 South Korea; 22 Monterotondo Mouse Clinic, Italian National Research Council (CNR), Institute of Cell Biology and Neurobiology, Adriano Buzzati-Traverso Campus, Via Ramarini, I-00015 Monterotondo Scalo, Italy; 23 International Mouse Phenotyping Consortium, San Anselmo, CA 94960 USA; 24 National Institutes of Health, Bethesda, MD 20205 USA; 25 The Wellcome Trust Sanger Institute, Wellcome Genome Campus, Hinxton, Cambridge, CB10 1SA UK; 26 German Mouse Clinic, Institute of Experimental Genetics, Helmholtz Zentrum München, German Research Center for Environmental Health, Ingolstädter Landstraße 1, 85764 Neuherberg, Germany; 27 Department of Ophthalmology & Vision Science, School of Medicine, U.C. Davis, Sacramento, CA 95817 USA; 28 Monterotondo Mouse Clinic, Italian National Research Council (CNR), Institute of Cell Biology and Neurobiology, Adriano Buzzati-Traverso Campus, Via Ramarini, I-00015 Monterotondo Scalo, Italy

Correction to: *Communications Biology* 10.1038/s42003-018-0226-0; published online 21 December 2018

In the original published version of the article, Valerie Vancollie was mistakenly omitted from the list of members of the International Mouse Phenotyping Consortium. In addition, recognition of funding from Wellcome Trust grant WT098051 was mistakenly omitted from the Acknowledgements. The errors have been corrected in both the PDF and HTML versions of the paper.

