# Supplementary material for: Identification of genes required for eye development by high-throughput screening of mouse knockouts
Source: Commun Biol. 2018 Dec 21;1:236. doi: 10.1038/s42003-018-0226-0 (PMC6303268; doi:10.1038/s42003-018-0226-0)
Supplement: Supplementary file 1 — Supplementary Information [file 42003_2018_226_MOESM1_ESM.docx]

**Supplementary Figure 1:** ReviGO interactive graph of GO terms enriched in a list of genes associated with the eye phenotype (“All”). Similar GO terms are linked by edges and node colour and size are correlated with modified Fisher exact P-values of GO terms.


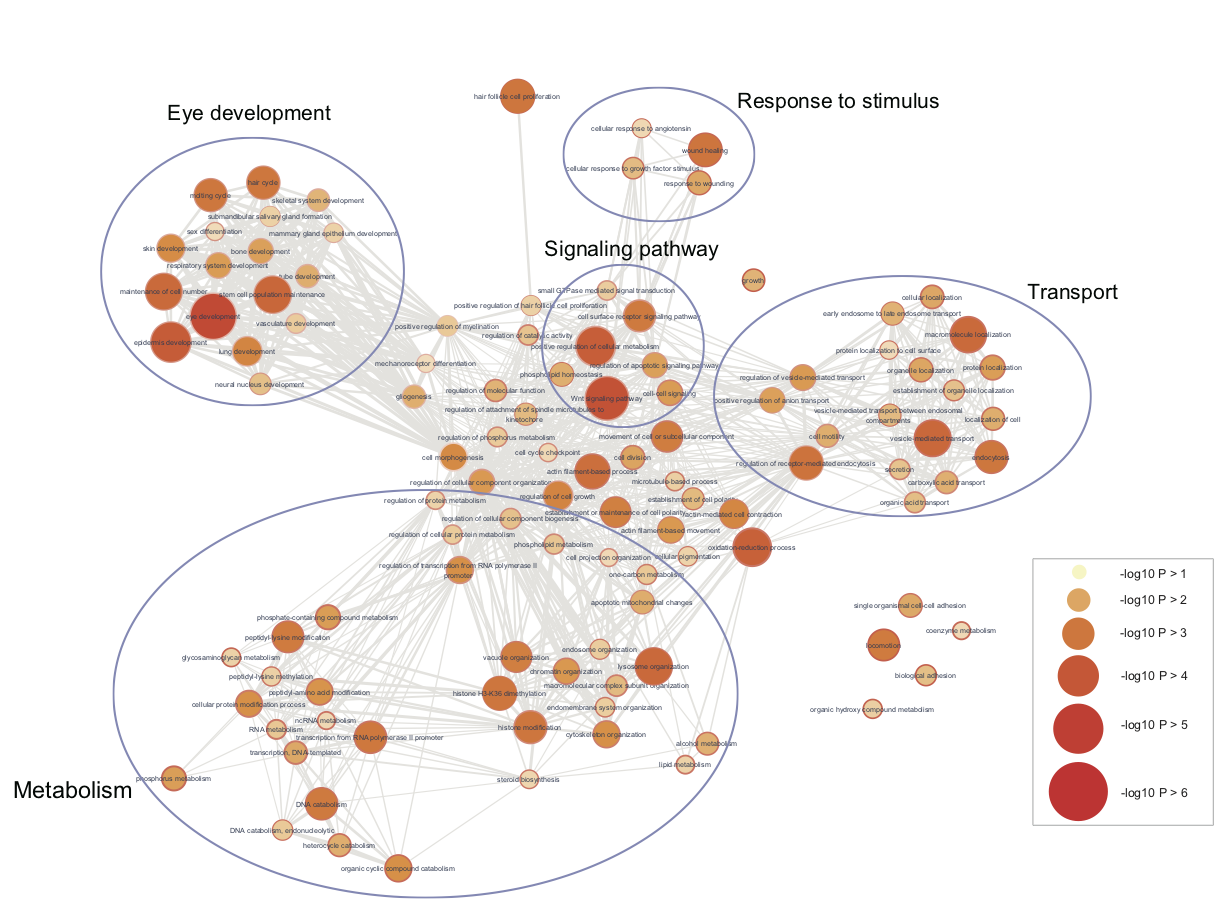


**Supplementary Figure 2:** ReviGO interactive graph of GO terms enriched in a list of genes associated with the eye phenotype (“Known”). Similar GO terms are linked by edges and node colour and size are correlated with modified Fisher exact P-values of GO terms.


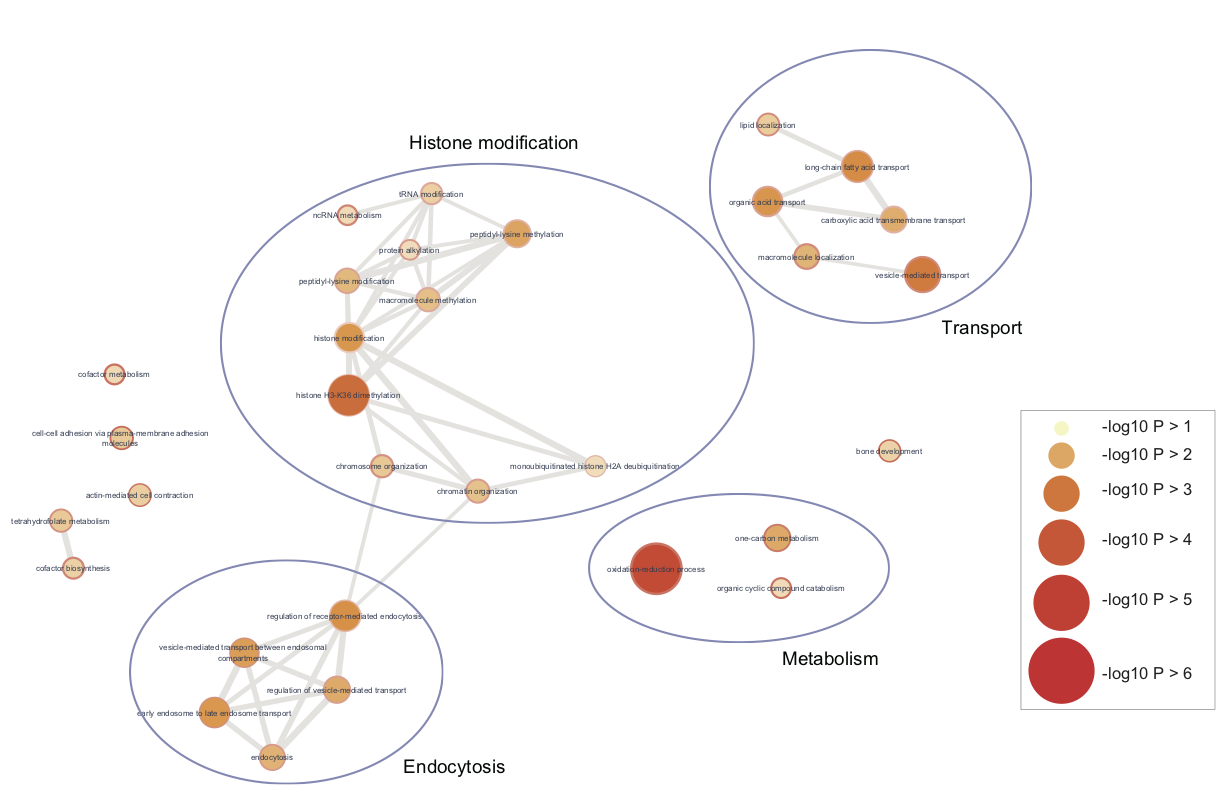


**Supplementary Figure 3:** ReviGO interactive graph of GO terms enriched in a list of genes associated with the eye phenotype (“Novel”). Similar GO terms are linked by edges and node colour and size are correlated with modified Fisher exact P-values of GO terms.


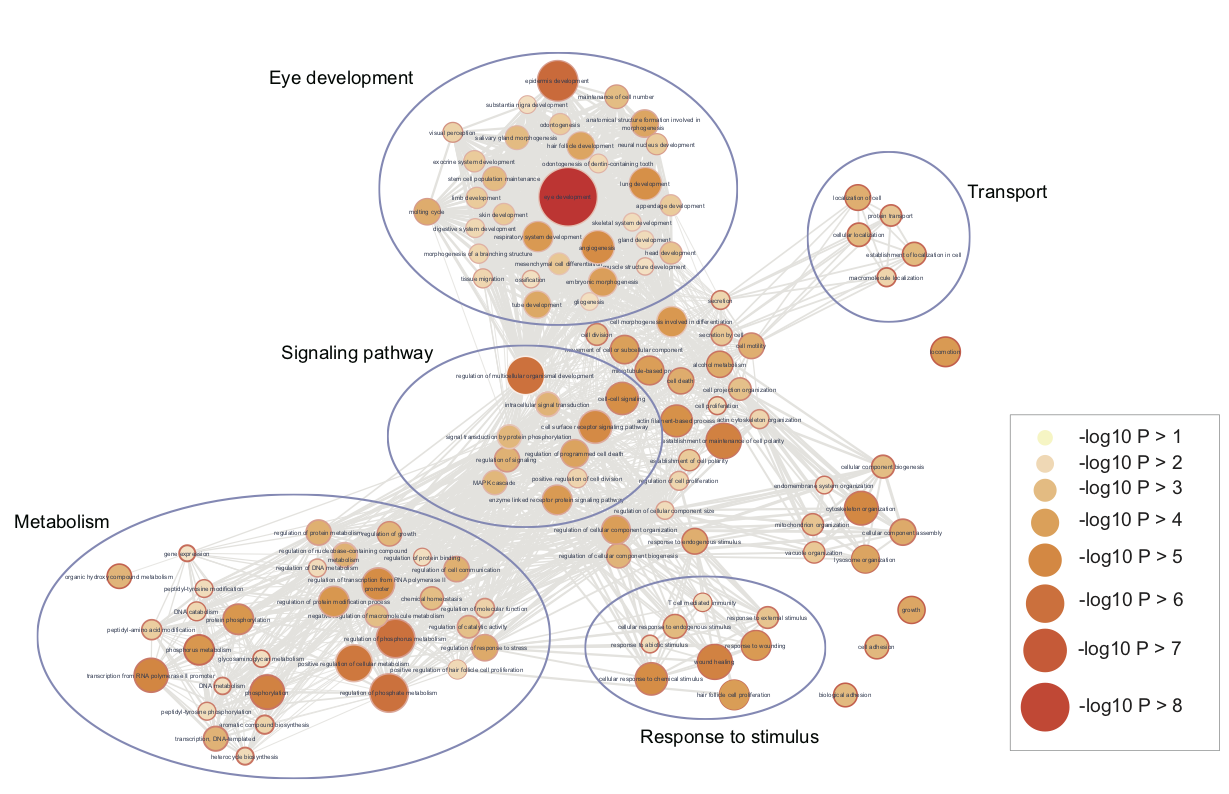


**Supplementary Figure 4:** A network model describing protein-protein interactions among genes associated with the eye phenotype. Node colour represents the eye location of genes.

**
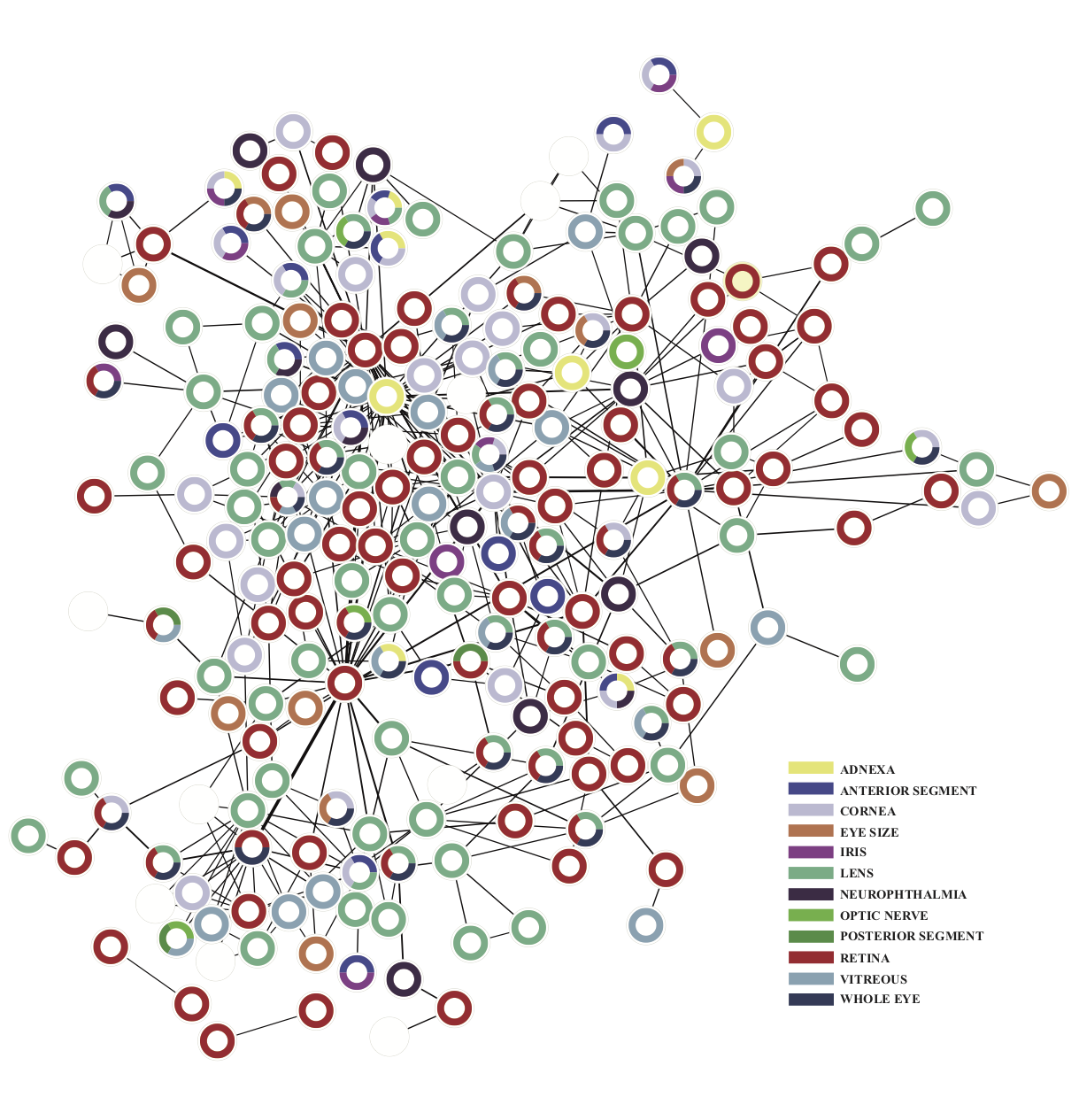
**

**Supplementary Table 1**: Complete gene and phenotype information. The gene, novelty of the gene (Novel Gene, Novel Phenotype, and Known Phenotype), Pubmed identification numbers (PMID), described human disease, phenotype ocular location, phenotyping center that described the phenotype, and the zygosity of the knockout mouse displaying the phenotype are presented.

| *Gene* | *Gene Name* | *Novel Gene* | *Novel Phenotype* | *Known Pheno type* | *PMID/PMC* | *PMID/PMC Knockout* | *Human Disease* | *Ocular Location* | *Specific Phenotype* | *IMPC Center* | *Zygosity* |
| --- | --- | --- | --- | --- | --- | --- | --- | --- | --- | --- | --- |
| 2700054A10Rik | RIKEN cDNA 2700054A10 gene | X |  |  |  |  |  | Eye Size | Anophthalmia | UC Davis | Hom |
| 2900026A02Rik | RIKEN cDNA 2900026A02 gene | X |  |  |  |  |  | Retina | Abnormal retina morphology | ICS | Hom |
| 4932438A13Rik | RIKEN cDNA 4932438A13 gene | X |  |  |  |  |  | Lens | Cataract | HMGU | Het |
| 5330417C22Rik | RIKEN cDNA 5330417C22 gene | X |  |  |  |  |  | Vitreous | Abnormal vitreous body morphology | ICS | Het |
| Aak1 | AP2 associated kinase 1 | X |  |  |  |  |  | Anterior Segment | Abnormal eye anterior chamber depth | ICS | Hom |
| Abcb11 | ATP-binding cassette, sub-family B (MDR/TAP), member 11 | X |  |  |  | 22619174 | Progressive familial intrahepatic cholestases | Retina | Abnormal retina morphology | HMGU | Hom |
| Abcd1 | ATP-binding cassette, sub-family D (ALD), member 1 | X |  |  |  | 9418970 | X-linked Adrenoleukodystrophy, Addison's Disease | Lens | Cataract | WTSI | Hemi |
| Abhd11 | Abhydrolase domain containing 11 | X |  |  |  |  | Williams-Beuren syndrome | Eye Size | Microphthalmia | UC Davis | Hom |
| Abi2 | Abl-interactor 2 |  | X |  |  | 15572692 |  | Anterior Segment | Abnormal cornea morphology, abnormal iris pigmentation | TCP | Hom |
| Ackr3 | Atypical chemokine receptor 3 |  | X |  |  | 17804806 | Glioma | Retina | Decreased total retina thickness | ICS | Het |
| Acsl5 | Acyl-CoA Synthetase Long-Chain Family Member 5 | X |  |  |  |  | Glioma and Chronic Intestinal Vascular Insufficiency | Anterior Segment | Corneal opacity, abnormal lens morphology | UC Davis | Hom |
| Adam32 | A disintegrin and metallopeptidase domain 32 | X |  |  |  |  |  | Neurophthalmia | Impaired pupillary reflex | JAX | Hom |
| Adamts18 | A disintegrin-like and metallopeptidase (reprolysin type) with thrombospondin type 1 motif, 18 |  |  | X | 24874986 | 27638769 | Microcornea, Myopic Chorioretinal Atrophy, Telecanthus and Knobloch Syndrome | Lens | Cataract | UC Davis | Hom |
| Adipor1 | adiponectin receptor 1 |  |  | X | 26662040 | 25736573 | Retinitis Pigmentosa, obesity | Retina | Abnormal retina morphology | MRC Harwell | Hom |
| Adsl | Adenylosuccinate Lyase | X |  |  |  |  |  | Retina | Abnormal retinal vasculature morphology | BCM | Het |
| Agap1 | ArfGAP with GTPase domain, ankyrin repeat and PH domain 1 | X |  |  |  |  |  | Whole Eye | Corneal opacity, fused cornea and lens, abnormal iris morphology, cataract, persistence of hyaloid vasculature | WTSI | Hom |
| Agl | Amylo-Alpha-1, 6-Glucosidase, 4-Alpha-Glucanotransferase | X |  |  |  | 25092169 | Glycogen storage disease 3a | Lens | Cataract | MRC Harwell | Hom |
| Ahcyl1 | S-adenosylhomocysteine hydrolase-like 1 | X |  |  |  |  |  | Whole Eye | Abnormal cornea morphology, abnormal retina morphology | WTSI | Hom |
| Ahrr | Aryl-hydrocarbon receptor repressor |  |  | X | 24675095 |  |  | Retina | Abnormal retina morphology | JAX | Het |
| Ahsa2 | AHA1, activator of heat shock protein ATPase 2 | X |  |  |  |  |  | Retina | Decreased total retina thickness | ICS | Hom |
| Aire | Autoimmune Polyendocrine Syndrome Type 1. |  |  | X | 25926518 | 26238369 |  | Retina | Abnormal retina morphology | TCP | Hom |
| Akr1d1 | Aldo-keto reductase family 1, member D1 | X |  |  |  |  | Congenital Bile Acid Synthesis Defect | Vitreous | Persistence of hyaloid vasculature | JAX | Hom |
| Aktip | Thymoma viral proto-oncogene 1 interacting protein | X |  |  |  |  | Thymic Hyperplasia, Tenosynovitis | Retina | Abnormal retina morphology | TCP | Hom |
| Aldh16a1 | Aldehyde dehydrogenase 16 family, member A1 | X |  |  |  | 28254523 | Mast Syndrome and Gout | Retina | Abnormal retina morphology | WTSI | Hom |
| Aldh1l1 | Aldehyde dehydrogenase 1 family, member L1 | X |  |  |  |  | Pilocytic Astrocytoma | Whole Eye | Cataract, abnormal retinal vasculature morphology | HMGU | Hom |
| Aldh2 | Aldehyde dehydrogenase 2, mitochondrial |  | X |  |  |  | Alcohol sensitivity and dependence | Lens | Abnormal lens morphology | HMGU | Hom |
| Ankrd11 | Ankyrin Repeat Domain 11 | X |  |  |  |  | Kbg Syndrome, Intellectual Disability | Posterior Segment | Abnormal vitreous body morphology, abnormal retinal vasculature morphology | ICS | Het |
| Ankrd27 | Ankyrin Repeat Domain 27 | X |  |  |  |  |  | Retina | Decreased total retina thickness | ICS | Hom |
| Anxa6 | Annexin A6 | X |  |  |  |  | Neonatal Lupus Erythematosus, Papillary Hidradenoma | Eye Size | Abnormal eye size | WTSI | Hom |
| Aoc1 | Amine Oxidase, Copper Containing 1 | X |  |  |  |  | Phlyctenulosis, Radiation Proctitis | Retina | Abnormal retinal vasculature morphology | HMGU | Hom |
| Apoc4 | Apolipoprotein C-IV | X |  |  |  |  |  | Posterior Segment | Persistence of hyaloid vascular system, abnormal retinal morphology | HMGU | Hom |
| Aqp6 | Aquaporin 6 |  | X |  |  |  |  | Retina | Increased total retina thickness | HMGU | Hom |
| Arap3 | ArfGAP With RhoGAP Domain, Ankyrin Repeat And PH Domain 3 | X |  |  |  |  |  | Anterior Segment | Cataract, Impaired pupillary reflex | JAX | Hom |
| Arf3 | ADP-ribosylation factor 3 | X |  |  |  |  | Distal Hereditary Motor Neuropathy, Type II | Whole Eye | Abnormal lens morphology, abnormal retinal morphology | HMGU | Hom |
| Arhgap25 | Rho GTPase Activating Protein 25 | X |  |  |  | 27566826 |  | Retina | Abnormal retina morphology, abnormal retinal pigmentation | WTSI | Hom |
| Arhgap33 | Rho GTPase activating protein 33 |  | X |  | 25563673 |  |  | Whole Eye | Abnormal retinal vasculature morphology, microphthalmia | BCM | Het |
| Arhgef11 | Rho Guanine Nucleotide Exchange Factor 11 | X |  |  |  |  |  | Retina | Abnormal retina morphology | MRC Harwell | Hom |
| Arl6ip1 | ADP-ribosylation factor-like 6 interacting protein 1 | X |  |  | 26864484 |  | Spastic Paraplegia 61 | Retina | Abnormal retina morphology | MRC Harwell | Hom |
| Arvcf | Armadillo Repeat Gene Deleted In Velocardiofacial Syndrome | X |  |  |  |  | Velocardiofacial Syndrome | Lens | Cataract | WTSI | Hom |
| Ascc2 | Activating signal cointegrator 1 complex subunit 2 | X |  |  |  |  |  | Retina | Abnormal retinal inner nuclear layer morphology | BCM | Het |
| Ash1l | ASH1 Like Histone Lysine Methyltransferase | X |  |  |  |  | Autism spectrum disorder | Neurophthalmia | Impaired pupillary reflex | MARC | Het |
| Aspa | Aspartoacylase | X |  |  |  | 10894262 | Canavan Disease | Cornea | Corneal Opacity | HMGU | Hom |
| Asxl1 | Additional sex combs like 1 | X |  |  |  |  | Bohring-Opitz syndrome | Anterior Segment | Corneal opacity, Impaired pupillary reflex | WTSI | Het |
| Atf3 | Activating transcription factor 3 | X |  |  |  | 15199129 | Hypospadias, Bone Cancer | Retina | Abnormal retina morphology | MRC Harwell | Hom |
| Atp11a | ATPase Phospholipid Transporting 11A | X |  |  |  |  | Idiopathic and Cerebellar Ataxia, Mental Retardation And Dysequlibrium Syndrome | Retina | Abnormal retina morphology | WTSI | Het |
| Atp5s | ATP Synthase, H+ Transporting, Mitochondrial Fo Complex Subunit S | X |  |  |  |  |  | Adnexa | Narrow eye opening | MRC Harwell | Hom |
| Bahd1 | Bromo adjacent homology domain containing 1 | X |  |  |  | 26938916 |  | Retina | Decreased total retina thickness | ICS | Het |
| Baiap2l2 | BAI1 Associated Protein 2 Like 2 | X |  |  |  | 27278019 |  | Anterior Segment | Abnormal eye anterior chamber depth | ICS | Hom |
| Barx2 | BARX Homeobox 2 |  |  | X |  | 21750040 |  | Anterior Segment | Abnormal cornea morphology, cornea opacity, corneal vascularization, eyelids fail to open, abnormal eyelid morphology, narrow eye opening | MRC Harwell | Hom |
| Bbs5 | Bardet-Biedl Syndrome 5 |  |  | X |  |  | Bardet-Biedl Syndrome | Retina | Abnormal retina morphology | MRC Harwell | Hom |
| Bmp2k | BMP2 Inducible Kinase | X |  |  | 19927351 |  | Possible High Myopia | Lens | Abnormal lens morphology | JAX | Hom |
| Bms1 | BMS1, Ribosome Biogenesis Factor | X |  |  |  |  | Aplasia Cutis Congenita | Lens | Cataract | HMGU | Het |
| Brcc3 | BRCA1/BRCA2-Containing Complex Subunit 3 |  | X |  | 21596366 |  | Moyamoya Disease 4, Hypergonadotropic Hypogonadism, Facial Dysmorphism, Early Cataract | Cornea | Corneal opacity | MARC | Hom |
| C1qtnf5 | C1q and tumor necrosis factor related protein 5 |  |  | X | 28939808 | 27143553 | Retinal Degeneration, Late-Onset, Autosomal Dominant | Retina | Abnormal retina morphology | JAX | Hom, Het |
| Cant1 | Calcium Activated Nucleotidase 1 | X |  |  |  |  | Desbuquois Dysplasia | Retina | Abnormal retinal vasculature morphology | HMGU | Het |
| Cap2 | CAP, Adenylate Cyclase-Associated Protein, 2 |  |  | X | 26616005 | 26616005 |  | Lens | Cataract | HMGU | Het |
| Capn12 | Calpain 12 | X |  |  |  |  | Autosomal Recessive Congenital Ichthyosis | Lens | Cataract | HMGU | Hom |
| Cbx2 | Chromobox 2 | X |  |  | 9043087 |  | 46Xy Sex Reversal 5 and 46 Xy Gonadal Dysgenesis | Vitreous | Persistence of hyaloid vasculature | MRC Harwell | Het |
| Cdc42 | Cell Division Cycle 42 |  |  | X | 23372671 | 23372671 | Takenouchi-Kosaki Syndrome, Facial Dysmorphism-Camptodactyly Syndrome | Retina | Abnormal retina morphology | MRC Harwell | Het |
| Cdh10 | Cadherin 10 | X |  |  |  |  |  | Eye Size | Microphthalmia | UC Davis | Hom |
| Cdkal1 | CDK5 Regulatory Subunit Associated Protein 1 Like 1 | X |  |  |  | 21151568 | Diabetes Mellitus, Sapho Syndrome | Lens | Cataract | HMGU | Hom |
| Cdkn2a | Cyclin Dependent Kinase Inhibitor 2A |  |  | X | 16620915 | 8620534 | Numerous cancers | Lens | Cataract | WTSI | Hom |
| Cdsn | Corneodesmosin | X |  |  |  | 19596793 | Peeling Skin Syndrome 1, Hypotrichosis 2 | Whole Eye | Cataract, persistence of hyaloid vasculature, abnormal retinal vasculature morphology, increased retina thickness | HMGU | Hom |
| Cenpe | Centromere Protein E | X |  |  |  |  | Microcephaly 13, Seckel Syndrome | Lens | Cataract | HMGU | Het |
| Chic2 | Cysteine Rich Hydrophobic Domain 2 | X |  |  |  |  |  | Anterior Segment | Iris synechia | MRC Harwell | Het |
| Chst5 | Carbohydrate Sulfotransferase 5 |  | X |  |  | 21440637 | Colon Mucinous Adenocarcinoma | Whole Eye | Abnormal lens morphology, abnormal retinal morphology | HMGU | Het |
| Cisd2 | CDGSH Iron Sulfur domain 2 |  | X |  |  | 19451219 | Wolfram Syndrome 2 | Eye Size | Macrophthalmia | MRC Harwell | Hom |
| Cln6 | CLN6, Transmembrane ER Protein |  |  | X | 11727201 | 24124525 | Ceroid Lipofuscinosis | Retina | Abnormal retina morphology | TCP | Hom |
| Clstn1 | Calsyntenin 1 | X |  |  |  | 24966372 |  | Vitreous | Persistence of hyaloid vasculature | MRC Harwell | Hom |
| Col4a5 | Collagen Type IV Alpha 5 Chain |  |  | X | 27485810 | 15153557 | Alport Syndrome with retinopathy | Whole Eye | Abnormal lens morphology, persistence of hyaloid vasculature | MRC Harwell | Hemi |
| Coq6 | Coenzyme Q6, Monooxygenase | X |  |  |  |  |  | Vitreous | Persistence of hyaloid vasculature, abnormal vitreous body morphology | ICS | Het |
| Cpa2 | Carboxypeptidase A2 | X |  |  |  |  |  | Lens | Cataract | HMGU | Hom |
| Crb2 | Crumbs 2, Cell Polarity Complex Component |  | X |  | 12915475 | 24493795 | Murine retinal progenitor cell abnormalities, Human ventriculomegaly | Retina | Abnormal retina morphology | WTSI | Het |
| Csnk1g2 | Casein Kinase 1 Gamma 2 | X |  |  |  |  |  | Retina | Decreased total retina thickness, impaired pupillary light reflex | HMGU | Hom |
| Cth | Cystathionase (cystathionine gamma-lyase) | X |  |  |  |  | Cystathioninuria and Hyperhomocysteinemia | Whole Eye | Cataract, abnormal retina morphology | HMGU | Hom |
| Ctla2b | Lipin 1 | X |  |  |  |  | Myoglobinuria | Retina | Abnormal retina morphology | TCP | Het |
| Cts6 | Cathepsin L | X |  |  |  |  |  | Retina | Abnormal retinal vasculature morphology | BCM | Hom |
| Cyb561 | Cytochrome B561 | X |  |  |  |  |  | Whole Eye | Correctopia, abnormal iris morphology, narrow eye opening, abnormal retinal blood vessel morphology | MRC Harwell | Hom |
| Cyb561a3 | Cytochrome B561 Family Member A3 | X |  |  |  |  |  | Lens | Cataract | HMGU | Hom |
| Cyba | Cytochrome B-245 Alpha Chain | X |  |  |  |  | Chronic Granulomatous Disease | Whole Eye | Cataract, persistence of hyaloid vasculature | WTSI | Hom |
| Cyp4b1 | Cytochrome P450 Family 4 Subfamily B Member 1 | X |  |  |  | 23748241 |  | Lens | Cataract | HMGU | Hom |
| Cyp4f14 | Cytochrome P450 Family 4 Subfamily F Member 12 | X |  |  |  | 22665481 |  | Lens | Cataract | HMGU | Hom |
| Cyp7a1 | Cytochrome P450 Family 7 Subfamily A Member 1 |  |  | X |  | 8663429 | Hypercholesterolemia | Lens | Cataract | HMGU | Hom |
| D16Ertd472e | Chromosome 21 Open Reading Frame 91 | X |  |  |  |  |  | Posterior Segment | Decreased total retina thickness, abnormal vitreous body morphology | ICS | Hom |
| D630023F18Rik | Chromosome 2 Open Reading Frame 80 | X |  |  |  |  |  | Retina | Abnormal retina morphology | WTSI | Hom |
| Dbn1 | Drebrin 1 | X |  |  |  |  |  | Retina | Abnormal retinal inner nuclear layer morphology, decreased total retina thickness | ICS | Het |
| Dbr1 | Debranching RNA Lariats 1 | X |  |  |  |  |  | Lens | Abnormal lens morphology | JAX | Het |
| Dcp2 | Decapping MRNA 2 | X |  |  |  | 21070968 | Isolated Cleft Palate | Vitreous | Abnormal vitreous body morphology | ICS | Het |
| Dctn5 | Dynactin Subunit 5 | X |  |  |  |  | Polycystic Kidney Disease 4 | Lens | Cataract | WTSI | Het |
| Dhx40 | DEAH-Box Helicase 40 |  | X |  |  |  | Norum Disease (fish-eye disease) | Retina | Abnormal retinal vasculature morphology | BCM | Hom |
| Dicer1 | Dicer 1, Ribonuclease III |  |  | X | 18463241 | 14528307 | Pleuropulmonary Blastoma, Goiter | Retina | Abnormal retina morphology | MRC Harwell | Het |
| Dixdc1 | DIX domain containing 1 |  | X |  | 16378754 | 27829159 |  | Retina | Abnormal retina morphology | JAX | Het |
| Dlec1 | Deleted In Lung And Esophageal Cancer 1 | X |  |  |  |  | Esophageal Cancer, Lung Cancer | Lens | Abnormal lens morphology | HMGU | Hom |
| Dnase1l2 | Deoxyribonuclease 1-like 2 | X |  |  |  | 28743926 |  | Whole Eye | Cataract, persistence of hyaloid vasculature | BCM | Hom |
| Dner | Delta/Notch Like EGF Repeat Containing | X |  |  |  | 15965470 |  | Lens | Cataract | HMGU | Hom |
| Dnm1l | Dynamin 1 Like |  | X |  | 28969390 | 19752021 | Role in glaucoma, optic nerve disease | Vitreous | Persistence of hyaloid vasculature, abnormal vitreous body morphology | ICS | Het |
| Dot1l | DOT1 Like Histone Lysine Methyltransferase | X |  |  |  | 26404731 | Gastric Cancer, Leukemia | Retina | Abnormal retinal vasculature morphology | WTSI | Het |
| Dpp9 | Dipeptidyl Peptidase 9 | X |  |  |  | 24223149 | Pulmonary Fibrosis | Lens | Abnormal lens morphology | HMGU | Het |
| Dsc2 | Desmocollin 2 | X |  |  |  |  | Arrhythmogenic Right Ventricular Dysplasia | Anterior Segment | Corneal opacity, corneal vascularization, narrow eye opening | WTSI | Hom |
| Dsg1b | Desmoglein 1 | X |  |  | 23024760 |  | Erythroderma, Palmoplantar Keratoderma, Hypotrichosis | Anterior Segment | Abnormal cornea morphology, abnormal eyelid morphology, abnormal iris morphology, cataract | WTSI | Hom |
| Dtnbp1 | Dystrobrevin Binding Protein 1 |  |  | X | 12923531 | 12923531 | Hermansky-Pudlak syndrome type 7 | Whole Eye | Abnormal iris pigmentation, abnormal retina morphology | TCP | Hom |
| Duoxa2 | Dual oxidase maturation factor 2 | X |  |  |  | 26273529 | Thyroid dyshormonogenesis 5 | Adnexa | Abnormal eyelid morphology | WTSI | Hom |
| Dusp11 | Dual Specificity Phosphatase 11 | X |  |  |  |  | Amyotrophic Lateral Sclerosis 1 | Whole Eye | Decreased total retina thickness, decreased cornea thickness | ICS | Hom |
| Eddm3b | Epididymal Protein 3B | X |  |  |  |  |  | Iris | Abnormal iris pigmentation | MARC | Hom |
| Efna4 | Ephrin A4 | X |  |  |  | 24488013 |  | Anterior Segment | Abnormal cornea morphology, Impaired pupillary reflex | JAX | Hom |
| Efna5 | Ephrin A5 |  |  | X |  | 26643403 |  | Anterior Segment | Cataract, Dyscoria | HMGU | Hom |
| Elk3 | ELK3, ETS Transcription Factor |  | X |  | 25203538 | 25203538 | Retinopathy | Retina | Abnormal retinal vasculature morphology | ICS | Hom |
| Ell2 | Elongation Factor For RNA Polymerase II 2 | X |  |  |  | 25238757 |  | Lens | Cataract | WTSI | Hom |
| Elmo3 | Engulfment and cell motility 3 | X |  |  |  |  |  | Cornea | Corneal opacity | UC Davis | Hom |
| Elmod1 | ELMO Domain Containing 1 | X |  |  |  |  |  | Neurophthalmia | Impaired pupillary reflex | MRC Harwell | Hom |
| Emc8 | ER membrane protein complex subunit 8 | X |  |  | 25715730 |  |  | Retina | Abnormal retinal blood vessel morphology | MRC Harwell | Het |
| Epc2 | Enhancer Of Polycomb Homolog 2 | X |  |  |  |  |  | Whole Eye | Cataract, abnormal retinal blood vessel morphology | HMGU | Het |
| Etfdh | Electron Transfer Flavoprotein Dehydrogenase | X |  |  |  |  | Glutaric Acidemia, Multiple Acyl-Coa Dehydrogenase Deficiency | Retina | Abnormal retinal vasculature morphology | HMGU | Het |
| Fabp3 | Fatty Acid Binding Protein 3 | X |  |  |  | 10224224 | Acute Myocardial Infarction, Intermediate Charcot-Marie-Tooth Neuropathy | Lens | Cataract | ICS | Het |
| Fam151b | Family With Sequence Similarity 151 Member B | X |  |  |  |  |  | Retina | Abnormal retina morphology | MRC Harwell | Hom |
| Fam162a | Family With Sequence Similarity 162 Member A | X |  |  |  |  |  | Lens | Cataract | HMGU | Hom |
| Fam216a | Family With Sequence Similarity 216 Member A | X |  |  |  |  |  | Lens | Cataract | HMGU | Hom |
| Fam49b | Family With Sequence Similarity 49 Member B | X |  |  |  |  |  | Retina | Abnormal retina morphology | TCP | Het |
| Fancl | Fanconi Anemia Complementation Group L | X |  |  |  |  |  | Adnexa | Narrow eyelid opening | MARC | Hom |
| Fbp2 | Fructose-Bisphosphatase 2 | X |  |  |  |  |  | Retina | Abnormal retinal vasculature morphology | HMGU | Het |
| Fbxw9 | F-Box And WD Repeat Domain Containing 9 | X |  |  |  |  |  | Eye Size | Anophthalmia | BCM | Hom |
| Fdx1 | Ferredoxin 1 | X |  |  |  |  |  | Whole Eye | Cataract, abnormal retina morphology | HMGU | Het |
| Fgf10 | Fibroblast Growth Factor 10 |  |  | X |  | 19407009 | Aplasia Of Lacrimal And Salivary Glands, Ladd Syndrome | Anterior Segment | Narrow eye opening, corneal opacity, Impaired pupillary reflex | MRC Harwell | Het |
| Fgf2 | Fibroblast Growth Factor 2 |  | X |  | 9736026 | 9736026 | Kaposi Sarcoma, Corneal Neovascularization | Retina | Abnormal retina morphology | MRC Harwell | Hom |
| Fgf7 | Fibroblast Growth Factor 7 |  | X |  |  | 8566750 | Mucositis, Apert Syndrome | Neurophthalmia | Impaired pupillary reflex | MRC Harwell | Hom |
| Fgf9 | Fibroblast growth factor 9 |  | X |  |  |  | de Multiple Synostoses Syndrome | Whole Eye | Cataract, abnormal retinal vasculature morphology | MRC Harwell | Het |
| Fgfr1op | Fgfr1 oncogene partner | X |  |  |  |  |  | Whole Eye | Cataract, abnormal retinal vasculature morphology | HMGU | Het |
| Fmc1 | Formation Of Mitochondrial Complex V Assembly Factor 1 Homolog | X |  |  |  |  |  | Whole Eye | Decreased total retina thickness, microphthalmia | BCM | Hom, Het |
| Foxo3 | Forkhead Box O3 | X |  |  |  | 28432353 |  | Retina | Abnormal retina morphology | JAX | Hom |
| Frmd6 | FERM Domain Containing 6 | X |  |  |  |  |  | Whole Eye | Cataract, narrow eye opening, persistence of hyaloid vasculature | MRC Harwell | Hom |
| Ftsj1 | FtsJ RNA Methyltransferase Homolog 1 | X |  |  |  |  | Mental Retardation, X-Linked | Retina | Abnormal retinal inner nuclear layer morphology, abnormal retinal vasculature morphology, decreased total retina thickness | BCM | Hom, Het |
| Fyb | FYN Binding Protein 1 | X |  |  |  | 11567141 | Thrombocytopenia | Lens | Cataract | HMGU | Hom |
| Galk2 | Galactokinase 2 | X |  |  |  |  |  | Lens | Cataract | HMGU | Hom |
| Gbp2 | Guanylate Binding Protein 2 | X |  |  |  | 23248289 |  | Lens | Cataract | HMGU | Hom |
| Gdi2 | GDP Dissociation Inhibitor 2 | X |  |  |  |  |  | Lens | Cataract | HMGU | Het |
| Ggps1 | Geranylgeranyl Diphosphate Synthase 1 | X |  |  |  |  |  | Lens | Cataract | HMGU | Het |
| Gja8 | Gap Junction Protein Alpha 8 |  | X |  | 11782410 | 11782410 | Cataract, Multiple Types and Cataract Microcornea Syndrome | Retina | Abnormal retina morphology | MRC Harwell | Hom |
| Glycam1 | Glycosylation Dependent Cell Adhesion Molecule 1 |  | X |  | 28446179 | 28446179 |  | Retina | Abnormal retina morphology | JAX | Het |
| Gmnn | Geminin, DNA Replication Inhibitor |  | X |  | 14973488 | 17054725 | Meier-Gorlin Syndrome, Embryonic lethal | Lens | Cataract | WTSI | Het |
| Gp6 | Glycoprotein VI Platelet | X |  |  |  | 17991808 | Bleeding Disorder | Anterior Segment | Abnormal eye anterior chamber depth | ICS | Hom |
| Gpsm2 | G Protein Signaling Modulator 2 | X |  |  | 15623799 |  | Chudley-Mccullough Syndrome, Sensorineural Deafness Type Dfnb | Whole Eye | Cataract, abnormal retina morphology | HMGU | Het |
| Gpx7 | Glutathione Peroxidase 7 | X |  |  |  | 23123197 | Childhood Kidney Cell Carcinoma | Optic Nerve | Absent optic nerve | UC Davis | Hom |
| Grb7 | Growth Factor Receptor Bound Protein 7 | X |  |  |  |  | Silver-Russell Syndrome, Breast Cancer | Cornea | Abnormal cornea morphology | WTSI | Hom |
| Grhl3 | Grainyhead Like Transcription Factor 3 |  | X |  | 18485343 | 18485343 | Van Der Woude Syndrome | Retina | Abnormal retina morphology | ICS | Het |
| Grm3 | Glutamate Metabotropic Receptor 3 | X |  |  |  | 18720515 | Schizophrenia | Eye Size | Microphthalmia | MRC Harwell | Hom |
| Grm6 | Glutamate Metabotropic Receptor 6 |  | X |  | 26628857 |  | Congenital Stationary Night Blindness | Anterior Segment | Cataract, Impaired pupillary reflex, mydriasis | MRC Harwell | Hom |
| Grtp1 | Growth Hormone Regulated TBC Protein 1 | X |  |  |  |  |  | Eye Size | Microphthalmia | TCP | Hom |
| Gspt2 | G1 To S Phase Transition 2 | X |  |  |  |  | Autistic Disorder, Visual Epilepsy | Neurophthalmia | Impaired pupillary reflex | JAX | Hom |
| Gtf2b | General transcription factor IIB | X |  |  |  |  | Herpes Simplex, Familial Atrial Fibrillation | Retina | Abnormal retina morphology | TCP | Hom |
| Hbs1l | HBS1 Like Translational GTPase | X |  |  |  | 23428869 | Sickle Cell Anemia | Whole Eye | Corneal opacity, abnormal retina morphology | WTSI | Hom |
| Hdac1 | Histone deacetylase 1 |  |  | X | 15944187 | 28028172 |  | Whole Eye | Cataract, abnormal retinal vasculature morphology | HMGU | Het |
| Hip1r | Huntingtin Interacting Protein 1 Related | X |  |  |  | 15121852 |  | Lens | Abnormal lens morphology | BCM | Hom |
| Hmbs | Hydroxymethylbilane Synthase | X |  |  |  | 26071363 | Porphyria | Whole Eye | Abnormal lens morphology, abnormal vitreous body morphology | JAX | Het |
| Hnf4a | Hepatocyte Nuclear Factor 4 Alpha | X |  |  |  |  | Mody, Fanconi Renotubular Syndrome, Maturity-Onset Diabetes Of The Young | Retina | Increased total retina thickness, abnormal retinal vasculature morphology | HMGU | Het |
| Hsd17b1 | Hydroxysteroid 17-Beta Dehydrog | X |  |  |  | 29180785 | Acute T Cell Leukemia, Endometriosis | Whole Eye | Abnormal lens morphology, abnormal retinal morphology | JAX | Hom |
| Hsf1 | Heat shock factor 1 |  | X |  |  | 27173427 | Synucleinopathy, Amyotrophic Lateral Sclerosis 1 | Retina | Abnormal retina morphology | JAX | Het |
| Hsf2bp | Heat Shock Transcription Factor 2 Binding Protein | X |  |  |  |  |  | Lens | Cataract | HMGU | Hom |
| Htr4 | 5-Hydroxytryptamine Receptor 4 | X |  |  |  |  | Irritable Bowel Syndrome | Neurophthalmia | Impaired pupillary reflex | MARC | Hom |
| Ift81 | Intraflagellar transport 81 |  | X |  | 28460050 | 28430876 | Spastic Paraplegia 36, Autosomal Dominant, Dengue Disease | Posterior Segment | Persistence of hyaloid vasculature, abnormal retinal vasculature morphology | HMGU | Het |
| Il18rap | Interleukin 18 Receptor Accessory Protein | X |  |  |  | 15843532 | Celiac Disease, Leprosy | Anterior Segment | Abnormal eye anterior chamber depth | ICS | Hom |
| Il2ra | Interleukin 2 Receptor Subunit Alpha | X |  |  |  |  | Immunodeficiency 41 With Lymphoproliferation And Autoimmunity | Anterior Segment | Abnormal eye anterior chamber depth | ICS | Hom |
| Ints10 | Integrator Complex Subunit 10 | X |  |  |  |  |  | Neurophthalmia | Impaired pupillary reflex | JAX | Het |
| Iqce | IQ motif containing E | X |  |  |  |  | Polydactyly, Postaxial, Type A7 | Retina | Abnormal retinal inner nuclear layer morphology | ICS | Hom |
| Itgb5 | Integrin Subunit Beta 5 |  |  | X | 22566632 | 22566632 | Villous Adenocarcinoma, Arrhythmogenic Right Ventricular Cardiomyopathy | Retina | Abnormal retina morphology | ICS | Hom |
| Itm2c | Integral Membrane Protein 2C | X |  |  |  |  |  | Lens | Cataract | HMGU | Het |
| Jam2 | Junctional Adhesion Molecule 2 | X |  |  |  | 16914739 |  | Retina | Abnormal retina morphology | JAX | Hom |
| Jmjd1c | Jumonji Domain Containing 1C | X |  |  |  | 27649575 | Central Nervous System Germinoma, Rett Syndrome | Iris | Mydriasis | WTSI | Het |
| Jmjd6 | Jumonji domain containing 6 | X |  |  |  | 17947579 | Deep Angioma, Intramuscular Hemangioma | Retina | Abnormal retinal vasculature morphology | HMGU | Het |
| Kansl1 | KAT8 Regulatory NSL Complex Subunit 1 |  | X |  | 28704368 | 28704368 | Koolen-De Vries Syndrome | Retina | Abnormal retinal outer nuclear laye rmorphology, decreased total retinal thickness | ICS | Het |
| Kat14 | Lysine Acetyltransferase 14 | X |  |  |  | 19103755 | Embryonic lethal | Anterior Segment | Corneal opacity, corneal vascularization, abnormal iris morphology, abnormal eyelid aperture, abnormal eye pigmentation | WTSI | Hom |
| Kdm8 | Lysine Demethylase 8 | X |  |  |  | 5295821 | Embryonic lethal | Iris | Abnormal iris pigmentation | ICS | Het |
| Khdrbs3 | KH RNA Binding Domain Containing, Signal Transduction Associated 3 | X |  |  |  | 5199341 | Transvestism, Fetishism | Lens | Cataract | HMGU | Hom |
| Kif5a | Kinesin Family Member 5A | X |  |  |  | 3332386 | Spastic Paraplegia | Neurophthalmia | Impaired pupillary reflex | JAX | Het |
| Klhdc2 | Kelch Domain Containing 2 | X |  |  |  | 5295821 | Osteochondrosis, Ischemic Bone Disease | Cornea | Corneal opacity | MRC Harwell | Het |
| Knstrn | Kinetochore Localized Astrin/SPAG5 Binding Protein | X |  |  |  | 4738695 |  | Retina | Abnormal retinal inner nuclear layer morphology | ICS | Hom |
| Lactb | Lactamase Beta | X |  |  |  |  | Lung Abscess, Otitis Media | Whole Eye | Abnormal lens morphology, persistent hyaloid vasculature, abnormal retinal vasculature | HMGU | Hom |
| Lama1 | Laminin Subunit Alpha 1 |  |  | X | 3215647 | 3259268 | Poretti-Boltshauser Syndrome, Myopia | Vitreous | Persistence of hyaloid vasculature | HMGU | Het |
| Lcp2 | Lymphocyte Cytosolic Protein 2 | X |  |  |  | 10377180 | B-Cell Linker Protein Deficiency | Lens | Cataract | ICS | Het |
| Limch1 | LIM And Calponin Homology Domains 1 | X |  |  |  |  |  | Retina | Abnormal retina morphology | JAX | Hom |
| Lmna | Lamin A/C |  |  | X | 14988595 |  | Hutchinson-Gilford Progeria, Mandibuloacral Dysplasia, Dense Deposit Disease (DDD) | Retina | Abnormal retina morphology | ICS | Het |
| Lrrc51 | Leucine rich repeat containing 51 | X |  |  |  |  | Deafness, Autosomal Recessive 63 | Retina | Abnormal retinal blood vessel morphology | BCM | Hom |
| Lrrk1 | Leucine Rich Repeat Kinase 1 | X |  |  |  | 23526378 | Snca-Related Parkinson Disease | Adnexa | Abnormal eyelid morphology | MRC Harwell | Hom |
| Lsm1 | LSM1 Homolog, MRNA Degradation Associated | X |  |  |  |  |  | Lens | Cataract | HMGU | Hom |
| Lss | Lanosterol Synthase |  |  | X | 29016354 | 16440058 | Cataract, Holoprosencephaly | Whole Eye | Abnormal lens morphology, abnormal retinal vasculature morphology | HMGU | Het |
| Lyst | Lysosomal Trafficking Regulator |  |  | X |  | 8717042 | Chediak-Higashi Syndrome | Whole Eye | Abnormal iris morphology, abnormal retina morphology | TCP | Hom |
| Map3k1 | Mitogen-Activated Protein Kinase Kinase Kinase 1 |  |  | X | 21862560 | 21862560 | 46Xy Sex Reversal 6 | Retina | Abnormal retina morphology | WTSI | Het |
| Map3k7 | Mitogen-Activated Protein Kinase Kinase Kinase 7 |  | X |  | 27094066 | 18573910 | Cardiospondylocarpofacial Syndrome, Frontometaphyseal Dysplasia 2 | Lens | Abnormal lens morphology | MRC Harwell | Het |
| Mapkapk2 | Mitogen-Activated Protein Kinase-Activated Protein Kinase 2 | X |  |  |  | 10559880 |  | Optic Nerve | Abnormal optic disc morphology | MRC Harwell | Hom |
| Mau2 | MAU2 Sister Chromatid Cohesion Factor | X |  |  |  |  |  | Lens | Cataract | WTSI | Het |
| Mcph1 | Microcephalin 1 |  |  | X | 23516444 | 23516444 | Microcephaly | Anterior Segment | Corneal opacity, corneal vascularization, abnormal eye pigmentation | WTSI | Hom |
| Mfsd8 | Major Facilitator Superfamily Domain Containing 8 |  |  | X | 28586915 | 24423645 | Ceroid Lipofuscinosis, Neuronal, 7 | Retina | Impaired pupillary reflex, abnormal retina morphology | MRC Harwell | Hom |
| Mib2 | Mindbomb E3 Ubiquitin Protein Ligase 2 | X |  |  |  | 18043734 | Gastritis, Familial Giant Hypertrophic, Left Ventricular Noncompaction | Retina | Abnormal retina morphology | ICS | Hom |
| Miga1 | Mitoguardin 1 | X |  |  |  |  |  | Lens | Cataract | HMGU | Hom |
| Mindy1 | MINDY Lysine 48 Deubiquitinase 1 | X |  |  |  |  |  | Cornea | Corneal opacity | MRC Harwell | Hom |
| Mipol1 | Mirror-Image Polydactyly 1 | X |  |  |  |  | Polydactyly, Fibular Hypoplasia | Lens | Cataract | HMGU | Hom |
| Mir96 | MicroRNA 96 |  | X |  | 28559309 | 28559309 | Deafness, Autosomal Dominant 50 | Cornea | Abnormal cornea morphology | WTSI | Hom |
| Mpdz | Multiple PDZ Domain Crumbs Cell Polarity Complex Component |  |  | X | 28556411 | 24118405 | Hydrocephalus, foveal dysplasia, inner retinal thinning | Retina | Abnormal retina morphology, abnormal retinal vasculature morphology | JAX | Hom |
| Mrpl22 | Mitochondrial Ribosomal Protein L22 | X |  |  |  |  |  | Neurophthalmia | Impaired pupillary reflex | JAX | Het |
| Mtf1 | Metal Regulatory Transcription Factor 1 | X |  |  |  | 15226267 | Enamel Erosion, Dentine Erosion | Whole Eye | Abnormal retina morphology, anophthalmia, microphthalmia | JAX | Het |
| Mthfd2 | Methylenetetrahydrofolate Dehydrogenase (NADP+ Dependent) 2 | X |  |  |  |  |  | Lens | Cataract | BCM | Het |
| Mthfsl | 5, 10-methenyltetrahydrofolate synthetase-like | X |  |  |  |  |  | Lens | Abnormal lens morphology, cataract | HMGU | Hom |
| Mvk | Mevalonate Kinase |  | X |  | 25390116 |  | Familial Mediterranean fever (FMF), hyperimmunoglobulinemia D syndrome (HIDS) | Eye Size | Microphthalmia | TCP | Het |
| Myo10 | Myosin X |  |  | X | 29229982 | 29229982 |  | Whole Eye | Corneal opacity, persistence of hyaloid vasculature, abnormal retina morphology, fused cornea and lens, impaired pupillary reflex | WTSI | Hom |
| Myo15 | Myosin XVA | X |  |  |  | 21236676 | Deafness, Autosomal Recessive 3 | Lens | Cataract | WTSI | Hom |
| Myo7a | Myosin VIIA |  |  | X | 19074810 | 21493626 | Usher Syndrome, Type 1B and Deafness, Autosomal Dominant 11 | Vitreous | Persistence of hyaloid vasculature | WTSI | Hom |
| Mysm1 | Myb Like, SWIRM And MPN Domains 1 | X |  |  |  | 26915790 | Hemorrhagic Cystitis | Adnexa | Narrow eye opening | WTSI | Hom |
| Nab2 | NGFI-A Binding Protein 2 | X |  |  |  | 28282643 | Hemangiopericytoma, Melanoma | Cornea | Abnormal cornea morphology, corneal mineralization | ICS | Hom |
| Nadk2 | NAD Kinase 2, Mitochondrial | X |  |  |  | 28923496 | 2,4-Dienoyl-Coa Reductase Deficiency, Hyperlysinemia | Vitreous | Persistence of hyaloid vasculature | JAX | Het |
| Nbas | Neuroblastoma Amplified Sequence |  |  | X | 26286438 |  | Short Stature, Optic Nerve Atrophy, Pelger-Huet Anomaly | Whole Eye | Abnormal lens morphology, abnormal optic disc morphology | HMGU | Het |
| Ncald | Neurocalcin Delta | X |  |  |  |  |  | Retina | Abnormal retinal blood vessel morphology, impaired pupillary reflex, mydriasis | JAX | Hom |
| Ncoa3 | Nuclear Receptor Coactivator 3 | X |  |  |  | 12650696 | Breast Cancer | Vitreous | Persistence of hyaloid vasculature | MRC Harwell | Het |
| Ncoa6 | Nuclear Receptor Coactivator 6 | X |  |  |  | 15161927 | Breast Cancer | Retina | Abnormal retina morphology | JAX | Het |
| Ncs1 | Neuronal Calcium Sensor 1 |  | X |  |  | 21737792 | Labyrinthitis, Tracheitis | Cornea | Abnormal cornea morphology, corneal mineralization | ICS | Hom |
| Ndfip2 | Nedd4 Family Interacting Protein 2 | X |  |  |  | 27048792 |  | Lens | Cataract | HMGU | Hom |
| Ndufa8 | NADH:Ubiquinone Oxidoreductase Subunit A8 | X |  |  |  |  | Mitochondrial Complex I Deficiency | Whole Eye | Cataract, decreased total retina thickness | HMGU | Het |
| Ndufs1 | NADH:Ubiquinone Oxidoreductase Core Subunit S1 |  | X |  | 15824269 |  | Mitochondrial Complex I Deficiency, Leigh Syndrome With Leukodystrophy | Lens | Cataract | HMGU | Het |
| Nedd4l | Neural Precursor Cell Expressed, Developmentally Down-Regulated 4-Like, E3 Ubiquitin Protein Ligase | X |  |  |  |  | Periventricular Nodular Heterotopia | Retina | Abnormal retina morphology | MRC Harwell | Hom |
| Nfil3 | Nuclear Factor, Interleukin 3 Regulated | X |  |  |  | 19749763 |  | Whole Eye | Abnormal cornea morphology, microphthalmia | UC Davis | Hom |
| Nfkb1 | Nuclear Factor Kappa B Subunit 1 |  |  | X | 23843455 | 9359707 | Immunodeficiency, Common Variable | Cornea | Abnormal cornea morphology | WTSI | Hom |
| Nol8 | Nucleolar Protein 8 | X |  |  |  |  |  | Anterior Segment | Abnormal eye posterior chamber depth | ICS | Het |
| Nos1ap | Nitric Oxide Synthase 1 Adaptor Protein | X |  |  |  | 27170476 | Long Qt Syndrome | Retina | Abnormal retinal blood vessel morphology | BCM | Hom |
| Nsun2 | NOP2/Sun RNA Methyltransferase Family Member 2 | X |  |  |  | 23401851 | Mental Retardation, Dubowitz Syndrome | Adnexa | Excessive tearing | WTSI | Het |
| Nt5dc1 | 5'-Nucleotidase Domain Containing 1 | X |  |  |  |  | COPD | Vitreous | Persistence of hyaloid vasculature | JAX | Hom |
| Nutf2 | Nuclear Transport Factor 2 | X |  |  |  |  |  | Lens | Abnormal lens morphology, cataract | HMGU | Het |
| Nxn | Nucleoredoxin | X |  |  |  | 20970343 | Vulvitis, Vulvar Disease | Whole Eye | Anophthalmia, abnormal eyelid morphology | BCM | Het |
| Oplah | 5-Oxoprolinase, ATP-Hydrolysing | X |  |  |  |  | 5-Oxoprolinase Deficiency, Glutathione Synthetase Deficiency | Lens | Abnormal lens morphology, cataract | HMGU | Hom |
| P2rx7 | Purinergic Receptor P2X 7 |  | X |  | 21983632 | 11016935 | Extrapulmonary Tuberculosis, Chronic Lymphocytic Leukemia | Lens | Cataract | HMGU | Hom |
| Pard3 | Par-3 Family Cell Polarity Regulator |  |  | X | 16431366 | 9716134 | Neural Tube Defects | Posterior Segment | Abnormal optic disc morphology, abnormal retina morphology | MRC Harwell | Het |
| Parp1 | Poly(ADP-Ribose) Polymerase 1 |  |  | X | 21124852 | 21124852 | Diphtheria, Hemorrhagic Cystitis | Retina | Decreased total retina thickness, abnormal retinal inner nuclear layer morphology | ICS | Hom |
| Pcdh12 | Protocadherin 12 | X |  |  |  | 15541725 | Aicardi-Goutieres Syndrome | Lens | Abnormal lens morphology | JAX | Hom |
| Pcdh18 | Protocadherin 18 | X |  |  |  |  | Hemophagocytic Lymphohistiocytosis, Patent Foramen Ovale | Cornea | Corneal opacity | UC Davis | Hom |
| Pdcd2 | Programmed Cell Death 2 | X |  |  |  | 20813103 | Chronic Fatigue Syndrome, Type 1 Diabetes Mellitus 10 | Adnexa | Abnormal eyelid morphology | WTSI | Het |
| Pdhx | Pyruvate Dehydrogenase Complex Component X | X |  |  |  | 8625829 | Lacticacidemia due To Pdx1 Deficiency, Primary Biliary Cirrhosis | Vitreous | Persistence of hyaloid vasculature | HMGU | Het |
| Pex3 | Peroxisomal Biogenesis Factor 3 | X |  |  |  |  | Peroxisome Biogenesis Disorder | Whole Eye | Abnormal eye size, abnormal cornea morphology | WTSI | Hom |
| Pfn1 | Profilin 1 | X |  |  |  | 11274401 | Amyotrophic Lateral Sclerosis 18 | Anterior Segment | Fused cornea and lens | WTSI | Het |
| Pgam5 | Phosphoglycerate mutase family member 5 | X |  |  |  | 25222142 | Autoimmune Lymphoproliferative Syndrome Type Iib | Retina | Decreased total retina thickness | ICS | Hom |
| Phykpl | 5-Phosphohydroxy-L-Lysine Phospho-Lyase | X |  |  |  |  | Phosphohydroxylysinuria | Retina | Abnormal retina morphology | ICS | Hom |
| Pip5k1c | Phosphatidylinositol-4-Phosphate 5-Kinase Type 1 Gamma | X |  |  |  | 24853942 | Lethal congenital contractural syndrome | Vitreous | Persistence of hyaloid vasculature | JAX | Het |
| Pitx2 | Paired Like Homeodomain 2 |  |  | X | 28611552 | 16203745 | Peter's Anomaly, Ring Corneal Dermoid, Axenfeld-Rieger Syndrome Type 1 | Whole Eye | Corneal opacity, abnormal optic disc morphology, dyscoria | MRC Harwell | Het |
| Pitx3 | Paired Like Homeodomain 3 |  |  | X | 29314435 | 25347445 | Anterior Segment Dysgenesis, Cataract 11 | Eye Size | Anophthalmia | JAX | Hom |
| Pkd1l2 | polycystic kidney disease gene 1-like 2 | X |  |  |  |  | Polycystic kidney disease 1 | Lens | Abnormal lens morphology | MRC Harwell | Hom |
| Pkig | CAMP-Dependent protein kinase inhibitor gamma | X |  |  |  |  |  | Whole Eye | Abnormal lens morphology, abnormal retinal vasculature morphology | HMGU | Hom |
| Pla2g10 | Phospholipase A2 Group X | X |  |  |  | 23349189 | Potter's Syndrome | Lens | Cataract | HMGU | Hom |
| Pla2g2e | Phospholipase A2 Group IIE | X |  |  |  | 24910243 |  | Lens | Cataract | HMGU | Hom |
| Plac8 | Placenta Specific 8 | X |  |  |  |  |  | Lens | Cataract | HMGU | Hom |
| Plekhg3 | Pleckstrin Homology And RhoGEF Domain Containing G3 | X |  |  |  |  |  | Anterior Segment | Abnormal eye anterior chamber depth | ICS | Hom |
| Plekhm1 | Pleckstrin Homology and RUN Domain Containing M1 | X |  |  |  |  | Osteopetrosis | Optic Nerve | Abnormal optic disc morphology | TCP | Hom |
| Ppfia2 | PTPRF Interacting Protein Alpha 2 | X |  |  |  |  |  | Whole Eye | Abnormal optic disc morphology, abnormal retinal blood vessel morphology | MRC Harwell | Hom |
| Prc1 | Protein Regulator of Cytokines 1 | X |  |  |  |  |  | Lens | Cataract | HMGU | Het |
| Prdm4 | PR/SET Domain 4 |  | X |  | 28228349 |  |  | Cornea | Abnormal cornea morphology | MRC Harwell | Hom |
| Primpol | Primase and polymerase (DNA-directed) |  | X |  | 25262353 |  | Myopia | Retina | Abnormal retina morphology | MRC Harwell | Hom |
| Prkab1 | Protein kinase, AMP-activated, beta 1 non-catalytic subunit | X |  |  | 21896769 |  | Obesity | Whole Eye | Abnormal retinal vasculature morphology, abnormal retinal blood vessel pattern, cataract | WTSI | Het |
| Prom2 | Prominin 2 | X |  |  |  |  | Chromophobe renal cell carcinoma | Retina | Abnormal retina morphology | JAX | Hom, Het |
| Ptma | Prothymosin, Alpha | X |  |  |  |  | Invasive malignant Thymoma | Cornea | Corneal opacity | UC Davis | Het |
| Ptpn23 | Protein Tyrosine Phosphatase, Non-Receptor Type 23 | X |  |  |  |  | Developmental and epileptic encephalopathies | Lens | Cataract | HMGU | Het |
| Ptpru | Protein Tyrosine Phosphatase, Receptor Type U | X |  |  |  |  |  | Vitreous | Persistence of hyaloid vasculature | JAX | Hom |
| R3hcc1l | R3H Domain and Coiled-coil Containing 1 Like | X |  |  |  |  |  | Vitreous | Persistence of hyaloid vasculature | JAX | Hom |
| Rab11a | RAB11A, Member RAS oncogene family |  | X |  | 27529348 | 27529348 | Barrett Esophagus | Vitreous | Persistence of hyaloid vasculature | JAX | Het |
| Rab11fip4 | RAB11 Family Interacting Protein 4 | X |  |  |  |  |  | Retina | Abnormal retina morphology | JAX | Hom |
| Rab19 | Member RAS Oncogene Family | X |  |  |  |  |  | Cornea | Corneal opacity | ICS | Hom |
| Rab35 | RAB35, Member RAS Oncogene Family | X |  |  |  |  | Charcot-Marie-Tooth Disease, Hypotrichosis | Lens | Cataract | HMGU | Het |
| Rab5a | RAB5A, Member RAS Oncogene Family | X |  |  |  |  | Motor Neuron Disease | Retina | Abnormal retina morphology | JAX | Hom |
| Raet1c | Retinoic acid early transcript gamma | X |  |  |  |  |  | Lens | Cataract | HMGU | Hom |
| Raf1 | Raf-1 Proto-oncogene, Serine/Threonine Kinase |  | X |  | 29271604 |  | Noonan Syndrome 5, Leopardy Syndrome 2 | Lens | Cataract | ICS | Het |
| Rbfox1 | RNA binging protein, Fox-1 Homolog 1 | X |  |  |  |  | Spinocerebellar Ataxia 2 | Vitreous | Abnormal vitreous body morphology | ICS | Het |
| Retreg3 | Reticulophagy regulator family member 3 | X |  |  |  |  |  | Retina | Abnormal retinal pigmentation | WTSI | Hom |
| Rfx7 | Regulatory Factor X7 | X |  |  |  |  |  | Retina | Abnormal retina morphology | MRC Harwell | Het |
| Rhbdf1 | Rhomboid 5 Homolog 1 | X |  |  |  |  | Palmoplantar keratoderma | Neurophthalmia | Impaired pupillary reflex | JAX | Het |
| Ric8a | RIC8 Guanine Nucleotide Exchange Factor A | X |  |  |  | 25641781 |  | Whole Eye | Persistence of hyaloid vasculature, impaired pupillary light reflex | JAX | Het |
| Rnf10 | Ring Finger Protein 10 | X |  |  |  |  |  | Whole Eye | Decreased total retina thickness, cataract, abnormal retinal blood vessel morphology, decreased total retina thickness | ICS | Hom, Het |
| Rnf144b | Ring finger protein 144B | X |  |  |  |  |  | Retina | Decreased total retina thickness | ICS | Hom |
| Rnf157 | Ring finger protein 157 | X |  |  |  |  |  | Lens | Cataract | WTSI | Hom |
| Rnf38 | Ring finger protein 38 | X |  |  |  |  |  | Retina | Abnormal retinal blood vessel morphology | BCM | Het |
| Rock1 | Rho-associated coiled-coil containing protein kinase 1 |  | X |  | 24326423 |  | Pediatric Osteosarcoma | Retina | Abnormal retina morphology | TCP | Het |
| Rpe65 | Retinal pigment epithelium 65 |  |  | X | 26626312 | 21551411 | Retinitis Pigmentosa 20, Leber Congenital Amaurosis 2 | Retina | Abnormal retina morphology, abnormal iris morphology, impaired pupillary light reflex | MRC Harwell | Hom |
| Rspo1 | R-spondin 1 | X |  |  |  | 23095882 | Mucositis, Palmoplantar hyperkeratosis | Whole Eye | Abnormal eye anterior chamber depth, abnormal retinal outer nuclear layer morphology, decreased total retina thickness, abnormal vitreous body morphology | ICS | Hom |
| Rtbdn | Retbindin | X |  |  |  |  |  | Retina | Abnormal retina morphology | WTSI | Hom |
| Rwdd1 | RWD Domain Containing 1 | X |  |  |  |  |  | Retina | Abnormal retina morphology | WTSI | Hom |
| S1pr3 | Sphingosine-1-phosphate receptor 3 | X |  |  |  |  |  | Retina | Abnormal retinal blood vessel morphology | BCM | Hom |
| Sarnp | SAP domain containing ribonucleoprotein | X |  |  |  |  | Hepatocellular carcinoma | Lens | Cataract | HMGU | Het |
| Satb1 | SATB homeobox 1 |  | X |  | 28781169 | 29127143 |  | Vitreous | Abnormal vitreous body morphology | ICS | Het |
| Scrib | Scribbled planar cell polarity | X |  |  |  |  | Neural Tube Defects | Retina | Abnormal retina morphology | TCP | Het |
| Sdc2 | Syndecan 2 | X |  |  |  |  | Trichorhinophalangeal Syndrome Type 1 | Lens | Cataract | HMGU | Hom |
| Sdc4 | Syndecan 4 | X |  |  |  | 28395201 |  | Neurophthalmia | Mydriasis | MRC Harwell | Het |
| Sema3f | Semaphorin 3F |  | X |  | 27309587 | 26856818 | Wallerian Degeneration | Lens | Cataract | MRC Harwell | Hom |
| Setd2 | SET domain containing 2 | X |  |  |  |  | Luscan-Lumish Syndrome, Sotos Syndrome 1 | Retina | Abnormal retina morphology | TCP | Hom |
| Setd6 | SET domain containing 6 | X |  |  |  |  |  | Cornea | Sclerocornea | JAX | Hom |
| Setmar | SET domain without mariner transposase fusion | X |  |  |  |  | Mantle Cell Lymphoma | Retina | Abnormal retina morphology, abnormal retinal pigmentation | WTSI | Hom |
| Sgo1 | Shugoshin 1 | X |  |  |  |  | Chronic atrial and intestinal dysrhythmia | Lens | Cataract | WTSI | Het |
| Shroom4 | Shroom family member 4 | X |  |  |  |  | Stocco dos Santos Syndrome | Cornea | Corneal opacity | TCP | Hemi |
| Siva1 | SIVA1 Apoptosis Inducing Factor | X |  |  |  |  | Scapuloperoneal myopathy | Lens | Cataract | HMGU | Hom |
| Slc20a2 | Solute carrier family 20 member 2 |  |  | X | 27380911 | 27380911 | Basal ganglia calcification Idiopathic 1 | Anterior Segment | Eyelids fail to open, abnormal iris morphology, cataract | WTSI | Hom, Het |
| Slc24a5 | Solute carrier family 24 member 5 |  |  | X | 18424845 | 18424845 | Oculocutaneous albinism | Whole Eye | Abnormal iris transillumination, abnormal retina morphology | JAX | Hom |
| Slc25a30 | Solute Carrier Family 25 Member 30 | X |  |  |  |  |  | Vitreous | Persistence of hyaloid vasculature | WTSI | Hom |
| Slc2a5 | Solute carrier family 2 member 5 | X |  |  |  | 26316589 | Hemophagocytic lymphohistiocytosis familial 5 | Whole Eye | Abnormal lens morphology, cataract, abnormal retina morphology | HMGU | Hom |
| Slc38a10 | Solute carrier family 38 member 10 | X |  |  |  |  |  | Retina | Abnormal retina morphology | ICS | Hom |
| Slc44a3 | Solute carrier family 44 member 3 | X |  |  |  |  |  | Lens | Abnormal lens morphology, cataract | HMGU | Hom |
| Slc7a11 | Solute Carrier Family 7 Member 11 | X |  |  | 23404113 | 29350434 | Dyscalculia, Kaposi sarcoma | Posterior Segment | Abnormal vitreous body morphology, abnormal optic disc morphology | ICS | Hom |
| Slc9a8 | Solute carrier family 9 member 8 |  |  | X | 25736793 | 25736793 | Proximal renal tubular acidosis | Retina | Abnormal retina morphology, abnormal retinal pigmentation | WTSI | Hom |
| Slx4 | SLX4 structure-specific endonuclease subunit | X |  |  |  | 21240276 | Fanconi anemia | Whole Eye | Anophthalmia, corneal opacity, abnormal corneal morphology, correctopia | WTSI | Hom |
| Smoc1 | SPARC related modular calcium binding 1 |  | X |  | 21194678 |  | Anophthalmia and microphthalmia with limb deformities | Anterior Segment | Narrow eye opening, mydriasis, impaired pupillary reflex | MRC Harwell | Hom |
| Smurf2 | SMAD specific E3 Ubiquitin Protein Ligase 2 | X |  |  |  |  |  | Whole Eye | Abnormal eyelid morphology, narrow eye opening, persistece of hyaloid vasculature | MRC Harwell | Hom, Het |
| Sorbs2 | Sorbin and SH3 Domain containing 2 | X |  |  |  | 26888934 | Facioscapulohumeral muscular dystrophy 1 | Retina | Abnormal retina morphology | JAX | Hom |
| Sparc | Secreted protein acidic and cysteine rich |  |  | X | 21384171 | 28122087 | Osteogenesis imperfecta | Lens | Abnormal lens morphology | WTSI | Hom |
| Spin1 | Spindlin 1 | X |  |  |  |  |  | Optic Nerve | Abnormal optic disc morphology | JAX | Het |
| Spns2 | Sphingolipid transporter 2 | X |  |  |  | 25356849 |  | Whole Eye | Abnormal eye pigmentation, abnormal eye size, excessive tearing, corneal opacity, corneal vascularization, abnormal iris morphology, narrow eye opening | WTSI | Hom |
| St6galnac3 | ST6 N-Acetylgalactosaminide Alpha-2,6-Sialyltransferase 3 | X |  |  |  |  |  | Lens | Cataract | JAX | Hom |
| Stra6l | Stimulated By Retinoic Acid 6 | X |  |  |  |  |  | Cornea | Corneal opacity | UC Davis | Hom |
| Synj1 | Synaptojanin 1 | X |  |  | 24392132 |  | Parkinson Disease 20 early onset, epileptic encephalopathy early infantile 53 | Vitreous | Persistence of hyaloid vasculature | MRC Harwell | Het |
| Tbce | Tubulin folding cofactor E |  |  | X | 17257873 |  | Kenny-Caffey Symdrome | Lens | Cataract | HMGU | Het |
| Tcf4 | Transcription Factor 4 |  | X |  | 29196769 |  | Pitt-Hopkins Syndrome, Fuchs Endothelial Dystrophy 3 | Retina | Abnormal retina morphology | WTSI | Het |
| Tcf7 | Transcription Factor 7 |  | X |  | 23524971 |  | Cleidocranial dysplasia | Lens | Cataract | ICS | Hom |
| Tcf7l1 | Transcription Factor 7 Like 1 | X |  |  |  |  | Anogenital Venereal Wart | Cornea | Corneal opacity | WTSI | Het |
| Tead1 | TEA Domain Transcription Factor 1 |  | X |  | 15016762 |  | Sveinsson Chorioreitnal Atrophy, Aicardi Syndrome | Eye Size | Microphthalmia | BCM | Het |
| Thpo | Thrombopoietin | X |  |  |  |  | Thrombocythemia | Cornea | Corneal opacity | UC Davis | Hom |
| Timp3 | TIMP Metallopeptidase Inhibitor 3 |  |  | X | 27601084 | 29134286 | Sorsby Fundus Dystrophy | Retina | Abnormal retinal blood vessel morphology | JAX | Hom |
| Tmem108 | Transmembrane protein 108 | X |  |  |  |  |  | Cornea | Abnormal cornea morphology | ICS | Hom |
| Tmem165 | Transmembrane protein 165 | X |  |  |  |  | Congenital disorder of glycosylation Type lik | Iris | Abnormal iris morphology | WTSI | Hom |
| Tmem189 | Transmembrane protein 189 | X |  |  |  |  | Fish Allergy | Retina | Abnormal retina morphology | WTSI | Hom |
| Tmem63b | Transmembrane protein 63b | X |  |  |  |  |  | Cornea | Abnormal cornea morphology | ICS | Het |
| Tmem79 | Transmembrane protein 79 | X |  |  |  | 24060273 | Angular Cheilitis | Retina | Abnormal retina morphology | JAX | Hom |
| Tnpo2 | Transportin 2 | X |  |  |  |  |  | Retina | Abnormal retinal blood vessel morphology | BCM | Hom |
| Tomm40 | Translocase of outer mitochondrial membrane 40 |  | X |  | 24146538 |  | Early-onset autosomal dominant alzheimer disease | Lens | Cataract | JAX | Het |
| Trmt10a | TRNA Methyltransferase 10A | X |  |  |  |  | Microcephaly, short stature, and impaired glucose metabolism 1 | Lens | Cataract | WTSI | Hom |
| Trrap | Transformation/Transcription domain associated protein | X |  |  |  |  | Lung large cell carcinoma | Cornea | Abnormal cornea morphology | WTSI | Het |
| Tsfm | Ts translation elongation factor, mitochondrial |  | X |  | 25037205 |  | Combined oxidative phosphorylation deficiency 3 | Retina | Abnormal retina morphology | WTSI | Het |
| Tuft1 | Tuftelin 1 | X |  |  |  |  | X-linked amelogenesis imperfecta hypoplastic/hypomaturation 2 | Adnexa | Abnormal eyelid morphology, narrow eye opening | WTSI | Hom |
| Tyw1 | TRNA-YW Synthesizing Protein 1 Homolog | X |  |  |  |  |  | Neurophthalmia | Impaired pupillary reflex | JAX | Hom |
| Ucp1 | Uncoupling protein 1 |  | X |  | 23033381 | 28679625 | Obesity | Lens | Cataract | HMGU | Hom |
| Uqcrb | Ubiquinol-Cytochrome C Reductase Binding Protein | X |  |  |  |  | Mitochondrial Complex Iii Deficiency | Lens | Cataract | HMGU | Het |
| Usp50 | Ubiquitin Specific Peptidase 50 | X |  |  |  |  |  | Iris | Dyscoria | MARC | Het |
| Vopp1 | Vesicular, overexpressed in cancer, prosurvival protein 1 | X |  |  |  |  |  | Neurophthalmia | Impaired pupillary reflex | JAX | Hom |
| Vps37d | VPS37D, ESCRT-I Subunit | X |  |  |  |  | Williams-Beuren Syndrome | Retina | Abnormal retinal inner nuclear layer morphology, decreased total retina thickness | BCM | Het |
| Vwa8 | Von Willebrand factor A domain containing 8 | X |  |  |  |  |  | Retina | Abnormal retinal vasculature morphology | HMGU | Hom |
| Wars | Tryptophanyl-TRNA Synthetase | X |  |  |  |  | Reflex Epilepsy | Lens | Cataract | HMGU | Het |
| Wsb2 | WD repeat and SOCS box containing 2 | X |  |  |  |  |  | Retina | Decreased total retinal thickness, abnormal retinal vasculature morphology, abnormal retinal vasculature morphology | HMGU | Hom |
| Xxylt1 | Xyloside Xylosyltransferase 1 | X |  |  |  |  |  | Retina | Abnormal retina morphology | WTSI | Hom |
| Yae1d1 | Yae1 Domain Containing 1 | X |  |  |  |  |  | Lens | Cataract | HMGU | Het |
| Ydjc | YdjC Chitooligosaccharide Deacetylase Homolog | X |  |  |  |  |  | Retina | Abnormal retina morphology | WTSI | Hom |
| Yipf5 | Yip1 domain family member 5 | X |  |  |  |  | Pleomorphic adenoma carcinoma | Cornea | Increased and decreased cornea thickness | ICS | Het |
| Ylpm1 | YLP motif containing 1 | X |  |  |  |  | Early-onset familial Alzheimer disease | Whole Eye | Cataract, Persistent of hyaloid vasculature | JAX | Het |
| Ywhaz | Tyrosine 3-monooxygenase/tryptophan 5-monooxygenase activation protein zeta | X |  |  |  |  |  | Retina | Abnormal retina morphology | TCP | Het |
| Zbtb24 | Zinc Finger And BTB Domain Containing 24 | X |  |  |  |  | Immunodeficiency-centromeric instability facial anomalies syndrome 2 | Retina | Abnormal retinal vasculature morphology | HMGU | Het |
| Zbtb4 | Zinc Finger And BTB Domain Containing 4 | X |  |  |  |  |  | Vitreous | Persistence of hyaloid vasculature | JAX | Hom |
| Zc3hc1 | Zinc finger C3HC-type containing 1 | X |  |  |  |  |  | Cornea | Abnormal cornea morphology | WTSI | Hom |
| Zdhhc23 | Zinc finger DHHC-type containing 23 | X |  |  |  |  |  | Cornea | Corneal opacity | UC Davis | Hom |
| Zfp182 | Zinc finger protein 182 | X |  |  |  |  |  | Lens | Cataract | WTSI | Hemi |

Supplementary Table 2. A list of GO terms of “GOTERM_BP_FAT” with modified Fisher exact P-values less than 0.05 and their associated genes.

| Term | Count | P-Value | Genes | Benjamini |
| --- | --- | --- | --- | --- |
| *GO:0007423~sensory organ development* | 27 | 2.63.E-05 | GJA8, ADAMTS18, FGF9, MYO7A, DICER1, ABI2, FGF10, RPE65, MAPKAPK2, SDC4, SCRIB, C1QTNF5, CRB2, MAP3K1, SMOC1, PITX3, PITX2, GRHL3, SPARC, SLC7A11, RAB11FIP4, LAMA1, HDAC1, JMJD6, GRM6, MYO15, MIR96 | 1.07.E-01 |
| *GO:0001654~eye development* | 21 | 3.10.E-05 | ADAMTS18, GJA8, FGF9, FGF10, ABI2, GRHL3, RPE65, SLC7A11, SCRIB, RAB11FIP4, LAMA1, C1QTNF5, HDAC1, CRB2, JMJD6, MAP3K1, SMOC1, GRM6, MIR96, PITX3, PITX2 | 6.47.E-02 |
| *GO:0016055~Wnt signaling pathway* | 20 | 5.98.E-05 | DIXDC1, TCF7, SPIN1, FGF9, CSNK1G2, FGF10, NFKB1, GRHL3, FOXO3, PTPRU, TCF7L1, SCRIB, CDC42, RSPO1, HDAC1, NXN, RAB5A, LRRK1, FGF2, PITX2 | 8.24.E-02 |
| *GO:0198738~cell-cell signaling by wnt* | 20 | 6.32.E-05 | DIXDC1, TCF7, SPIN1, FGF9, CSNK1G2, FGF10, NFKB1, GRHL3, FOXO3, PTPRU, TCF7L1, SCRIB, CDC42, RSPO1, HDAC1, NXN, RAB5A, LRRK1, FGF2, PITX2 | 6.60.E-02 |
| *GO:0008544~epidermis development* | 17 | 1.45.E-04 | SATB1, FGF7, ROCK1, MYO7A, DICER1, FGF10, GRHL3, DNASE1L2, SCRIB, CDC42, CDKN2A, BARX2, NCOA3, HDAC1, MIR96, MYSM1, TMEM79 | 1.17.E-01 |
| *GO:0031325~positive regulation of cellular metabolic process* | 74 | 1.53.E-04 | FGF7, FGF9, HIP1R, DICER1, FGF10, NFKB1, FOXO3, SDC4, PDCD2, MAP3K7, CDC42, CDKN2A, BARX2, RSPO1, HSF1, CYP7A1, SETMAR, PITX3, FGF2, PITX2, KHDRBS3, BRCC3, NOS1AP, ACKR3, GRHL3, GTF2B, ELL2, ARHGEF11, HNF4A, MTF1, NCOA3, ASH1L, NCOA6, EFNA5, LRRK1, LCP2, CLN6, PARD3, ELK3, MAPKAPK2, DTNBP1, TCF7L1, SCRIB, SLX4, PFN1, AHRR, MAP3K1, FAM162A, RNF10, TCF4, MYSM1, ACSL5, THPO, DIXDC1, RNF144B, PLA2G10, ASXL1, LMNA, RAF1, NDFIP2, TEAD1, CENPE, PLAC8, DOT1L, CYBA, P2RX7, ATF3, PROM2, AKTIP, HDAC1, AIRE, FABP3, KDM8, PARP1 | 1.04.E-01 |
| *GO:0060070~canonical Wnt signaling pathway* | 14 | 1.73.E-04 | DIXDC1, CDC42, TCF7, RSPO1, HDAC1, FGF9, RAB5A, FGF10, NFKB1, FOXO3, PTPRU, FGF2, LRRK1, TCF7L1 | 1.01.E-01 |
| *GO:0043010~camera-type eye development* | 18 | 1.87.E-04 | GJA8, FGF10, ABI2, GRHL3, RPE65, SLC7A11, SCRIB, RAB11FIP4, LAMA1, C1QTNF5, HDAC1, CRB2, JMJD6, MAP3K1, GRM6, MIR96, PITX3, PITX2 | 9.58.E-02 |
| *GO:0055114~oxidation-reduction process* | 33 | 2.05.E-04 | ALDH1L1, HSD17B1, FDX1, ABCD1, RPE65, MTHFD2, CYP7A1, TYW1, ETFDH, GPX7, NDUFS1, AGL, ACSL5, GDI2, NDUFA8, ADIPOR1, CYP4F14, PCDH12, CYB561A3, ALDH16A1, CYB561, CYP4B1, COQ6, CYBA, NXN, JMJD6, KDM8, ALDH2, ADSL, JMJD1C, AOC1, AKR1D1, UQCRB | 9.37.E-02 |
| *GO:1905114~cell surface receptor signaling pathway involved in cell-cell signaling* | 21 | 2.10.E-04 | DIXDC1, TCF7, SPIN1, FGF9, CSNK1G2, FGF10, NFKB1, GRHL3, FOXO3, PTPRU, TCF7L1, SCRIB, CDC42, RSPO1, HDAC1, NXN, RAB5A, DBN1, LRRK1, FGF2, PITX2 | 8.65.E-02 |
| *GO:0009893~positive regulation of metabolic process* | 78 | 2.36.E-04 | FGF7, FGF9, HIP1R, DICER1, FGF10, NFKB1, FOXO3, SDC4, PDCD2, MAP3K7, CDC42, CDKN2A, BARX2, RSPO1, HSF1, CYP7A1, SETMAR, NFIL3, PITX3, FGF2, PITX2, KHDRBS3, BRCC3, NOS1AP, PRKAB1, ACKR3, GRHL3, GTF2B, ELL2, ARHGEF11, NCOA3, HNF4A, MTF1, ASH1L, NCOA6, EFNA5, LRRK1, LCP2, CLN6, PARD3, FDX1, ELK3, MAPKAPK2, DTNBP1, TCF7L1, SCRIB, SLX4, PFN1, AHRR, MAP3K1, FAM162A, RNF10, NEDD4L, TCF4, MYSM1, ACSL5, THPO, DIXDC1, RNF144B, PLA2G10, ASXL1, LMNA, RAF1, NDFIP2, TEAD1, CENPE, PLAC8, DOT1L, CYBA, P2RX7, ATF3, PROM2, AKTIP, HDAC1, AIRE, FABP3, KDM8, PARP1 | 8.84.E-02 |
| *GO:0019827~stem cell population maintenance* | 11 | 3.12.E-04 | CDKN2A, MCPH1, DICER1, SETD6, RAF1, FGF10, TEAD1, FOXO3, JMJD1C, LSM1, TCF7L1 | 1.06.E-01 |
| *GO:0016192~vesicle-mediated transport* | 38 | 3.32.E-04 | PARD3, YWHAZ, HIP1R, MYO7A, SYNJ1, NCS1, PTPN23, PIP5K1C, MAPKAPK2, SDC4, DTNBP1, SCRIB, PFN1, CDC42, CHIC2, RSPO1, AAK1, RAB11A, NEDD4L, TMEM79, GDI2, DNM1L, NBAS, KIF5A, CSNK1G2, ACKR3, ELMO3, ANKRD27, CYBA, P2RX7, PROM2, AKTIP, JMJD6, ARF3, RAB35, GRTP1, RAB5A, YIPF5 | 1.04.E-01 |
| *GO:0010604~positive regulation of macromolecule metabolic process* | 73 | 3.42.E-04 | FGF7, FGF9, HIP1R, DICER1, FGF10, NFKB1, FOXO3, SDC4, PDCD2, MAP3K7, CDC42, CDKN2A, BARX2, RSPO1, HSF1, SETMAR, NFIL3, PITX3, FGF2, PITX2, KHDRBS3, BRCC3, NOS1AP, PRKAB1, ACKR3, GRHL3, GTF2B, ELL2, ARHGEF11, NCOA3, HNF4A, MTF1, ASH1L, NCOA6, EFNA5, LRRK1, LCP2, CLN6, PARD3, ELK3, MAPKAPK2, DTNBP1, TCF7L1, SCRIB, SLX4, PFN1, AHRR, MAP3K1, FAM162A, RNF10, NEDD4L, TCF4, MYSM1, THPO, DIXDC1, RNF144B, PLA2G10, ASXL1, LMNA, RAF1, NDFIP2, TEAD1, CENPE, PLAC8, DOT1L, P2RX7, ATF3, PROM2, AKTIP, HDAC1, AIRE, KDM8, PARP1 | 1.00.E-01 |
| *GO:0080171~lytic vacuole organization* | 7 | 3.46.E-04 | P2RX7, MFSD8, AKTIP, MYO7A, LYST, TMEM165, CLN6 | 9.49.E-02 |
| *GO:0007040~lysosome organization* | 7 | 3.46.E-04 | P2RX7, MFSD8, AKTIP, MYO7A, LYST, TMEM165, CLN6 | 9.49.E-02 |
| *GO:0033036~macromolecule localization* | 71 | 3.75.E-04 | FGF7, FGF9, PLEKHM1, MYO7A, PTPN23, PIP5K1C, FGF10, PEX3, VPS37D, CDC42, FRMD6, AGAP1, DUOXA2, ROCK1, ABCB11, KIF5A, PKIG, PTPRU, SARNP, ANKRD27, ARHGAP33, HNF4A, LYST, RAB5A, EFNA5, IFT81, PLA2G2E, DBN1, LCP2, ARL6IP1, BBS5, PARD3, YWHAZ, ABCD1, SCRIB, PFN1, ZDHHC23, C1QTNF5, APOC4, RAB11A, NEDD4L, TNPO2, 4932438A13RIK, ACSL5, ELMOD1, FYB, SPNS2, GDI2, DNM1L, PLA2G10, RHBDF1, LMNA, TOMM40, RAF1, NDFIP2, ATP11A, CENPE, P2RX7, STRA6L, AKTIP, HDAC1, ARF3, RAB35, GRTP1, MCPH1, FABP3, YIPF5, NUTF2, AHCYL1, SETD2, PARP1 | 9.64.E-02 |
| *GO:0098727~maintenance of cell number* | 11 | 3.85.E-04 | CDKN2A, MCPH1, DICER1, SETD6, RAF1, FGF10, TEAD1, FOXO3, JMJD1C, LSM1, TCF7L1 | 9.31.E-02 |
| *GO:0030029~actin filament-based process* | 25 | 5.47.E-04 | DIXDC1, PARD3, FGF7, CAP2, ROCK1, SHROOM4, BAIAP2L2, HIP1R, MYO7A, ABI2, ITGB5, FGF10, GRHL3, SDC4, DTNBP1, CDC42, PFN1, FRMD6, SORBS2, LIMCH1, MAP3K1, DSC2, EFNA5, NEDD4L, DBN1 | 1.23.E-01 |
| *GO:0048259~regulation of receptor-mediated endocytosis* | 8 | 7.69.E-04 | PARD3, PROM2, RSPO1, HIP1R, AAK1, SYNJ1, NEDD4L, DTNBP1 | 1.60.E-01 |
| *GO:0016569~covalent chromatin modification* | 21 | 7.77.E-04 | SATB1, SPIN1, BRCC3, KANSL1, ASXL1, CBX2, TRRAP, DOT1L, MAP3K7, EPC2, HDAC1, JMJD6, ASH1L, SETMAR, NCOA6, SETD6, KDM8, JMJD1C, SETD2, MYSM1, BAHD1 | 1.54.E-01 |
| *GO:0042060~wound healing* | 18 | 8.33.E-04 | ADAMTS18, PARD3, FGF7, DICER1, PIP5K1C, FGF10, GRHL3, SPARC, ELK3, SDC4, DTNBP1, SLC7A11, SCRIB, GP6, HNF4A, MAP3K1, LYST, FGF2 | 1.57.E-01 |
| *GO:0071335~hair follicle cell proliferation* | 3 | 9.03.E-04 | CDC42, DICER1, FGF10 | 1.62.E-01 |
| *GO:0097676~histone H3-K36 dimethylation* | 3 | 9.03.E-04 | ASH1L, SETMAR, SETD2 | 1.62.E-01 |
| *GO:0016570~histone modification* | 18 | 9.35.E-04 | SATB1, BRCC3, KANSL1, ASXL1, TRRAP, DOT1L, MAP3K7, EPC2, HDAC1, JMJD6, ASH1L, SETMAR, NCOA6, SETD6, KDM8, JMJD1C, SETD2, MYSM1 | 1.61.E-01 |
| *GO:0006897~endocytosis* | 21 | 9.75.E-04 | PARD3, DNM1L, CSNK1G2, HIP1R, MYO7A, SYNJ1, PIP5K1C, ACKR3, MAPKAPK2, ELMO3, DTNBP1, SCRIB, CYBA, CDC42, P2RX7, PROM2, RSPO1, JMJD6, AAK1, RAB5A, NEDD4L | 1.61.E-01 |
| *GO:0042303~molting cycle* | 9 | 9.87.E-04 | CDC42, FGF7, BARX2, HDAC1, DICER1, FGF10, DNASE1L2, MYSM1, TMEM79 | 1.57.E-01 |
| *GO:0042633~hair cycle* | 9 | 9.87.E-04 | CDC42, FGF7, BARX2, HDAC1, DICER1, FGF10, DNASE1L2, MYSM1, TMEM79 | 1.57.E-01 |
| *GO:0006366~transcription from RNA polymerase II promoter* | 48 | 1.07.E-03 | FGF9, YLPM1, DICER1, FGF10, CBX2, NFKB1, FOXO3, ELK3, TCF7L1, USP50, PFN1, AHRR, BARX2, CDKN2A, HSF1, CRB2, HSF2BP, RNF10, TCF4, PITX3, NFIL3, FGF2, MYSM1, PITX2, SATB1, TCF7, PKIG, ASXL1, LMNA, RAF1, TEAD1, GRHL3, PLAC8, ELL2, DOT1L, SARNP, ATF3, MTF1, PRDM4, HDAC1, HNF4A, NCOA3, NCOA6, ASH1L, AIRE, ZBTB4, SETD2, PARP1 | 1.63.E-01 |
| *GO:0006308~DNA catabolic process* | 5 | 1.17.E-03 | CDKN2A, HSF1, SETMAR, DICER1, DNASE1L2 | 1.71.E-01 |
| *GO:0007166~cell surface receptor signaling pathway* | 60 | 1.17.E-03 | SPIN1, FGF7, FGF9, HIP1R, FGF10, RPE65, NFKB1, FOXO3, MAP3K7, S1PR3, CDC42, RSPO1, AAK1, SEMA3F, FGF2, PITX2, ZC3HC1, CSNK1G2, ADIPOR1, ACKR3, GRHL3, PTPRU, HNF4A, MIB2, GRM6, RAB5A, EFNA5, EFNA4, DBN1, LRRK1, LCP2, SIVA1, ITGB5, MAPKAPK2, ITM2C, TIMP3, TCF7L1, SCRIB, CRB2, SORBS2, MAP3K1, DNER, THPO, FYB, DIXDC1, TCF7, IL2RA, BAIAP2L2, RHBDF1, LMNA, RAF1, COL4A5, LAMA1, P2RX7, ATF3, NXN, HDAC1, JMJD6, SMURF2, PARP1 | 1.66.E-01 |
| *GO:0090263~positive regulation of canonical Wnt signaling pathway* | 7 | 1.20.E-03 | DIXDC1, RSPO1, FGF9, FGF10, NFKB1, FGF2, LRRK1 | 1.63.E-01 |
| *GO:0040011~locomotion* | 44 | 1.27.E-03 | PARD3, FGF7, PTPN23, ABI2, FGF10, PIP5K1C, SDC4, SCRIB, SDC2, CDC42, PFN1, SORBS2, SEMA3F, MAP3K1, RAB11A, FGF2, MYSM1, PITX2, DIXDC1, SPNS2, PLA2G10, ROCK1, KIF5A, RHBDF1, LMNA, ADIPOR1, ACKR3, SPARC, PTPRU, ELMO3, RIC8A, LAMA1, MYO10, FGFR1OP, LYST, RAB5A, EFNA5, SMURF2, EFNA4, SETD2, ARAP3, DBN1, GRB7, CLN6 | 1.67.E-01 |
| *GO:0018205~peptidyl-lysine modification* | 15 | 1.28.E-03 | KANSL1, TRRAP, MAP3K7, DOT1L, EPC2, CTH, AHRR, CDKN2A, HDAC1, JMJD6, ASH1L, SETMAR, NCOA6, SETD6, SETD2 | 1.63.E-01 |
| *GO:0048839~inner ear development* | 12 | 1.30.E-03 | C1QTNF5, FGF9, MYO7A, MYO15, DICER1, FGF10, GRHL3, MAPKAPK2, SPARC, MIR96, SDC4, SCRIB | 1.61.E-01 |
| *GO:0009887~organ morphogenesis* | 33 | 1.33.E-03 | FGF7, FGF9, MYO7A, DICER1, FGF10, RPE65, AQP6, FOXO3, CDSN, SCRIB, CDC42, C1QTNF5, CDKN2A, BARX2, CRB2, ANKRD11, PITX3, FGF2, PITX2, TCF7, GMNN, ASXL1, TEAD1, GRHL3, LAMA1, P2RX7, NCOA3, HDAC1, NAB2, ASH1L, MYO15, MIR96, SETD2 | 1.59.E-01 |
| *GO:0006928~movement of cell or subcellular component* | 48 | 1.43.E-03 | PARD3, FGF7, MYO7A, PTPN23, ABI2, FGF10, SDC4, DTNBP1, SCRIB, SDC2, CDC42, PFN1, FRMD6, SORBS2, SEMA3F, MAP3K1, RAB11A, NEDD4L, FGF2, MYSM1, PITX2, DIXDC1, SPNS2, PLA2G10, ROCK1, KIF5A, RHBDF1, LMNA, ADIPOR1, CENPE, SPARC, PTPRU, ELMO3, RIC8A, LAMA1, MYO10, FGFR1OP, LYST, RAB5A, DSC2, EFNA5, SMURF2, IFT81, EFNA4, SETD2, ARAP3, DBN1, GRB7 | 1.66.E-01 |
| *GO:0045944~positive regulation of transcription from RNA polymerase II promoter* | 33 | 1.57.E-03 | DICER1, FGF10, NFKB1, FOXO3, ELK3, TCF7L1, PFN1, CDKN2A, BARX2, HSF1, RNF10, TCF4, PITX3, FGF2, MYSM1, PITX2, ASXL1, LMNA, TEAD1, RAF1, GRHL3, PLAC8, ELL2, DOT1L, ATF3, MTF1, NCOA3, HNF4A, HDAC1, ASH1L, NCOA6, AIRE, PARP1 | 1.76.E-01 |
| *GO:0007033~vacuole organization* | 11 | 1.70.E-03 | P2RX7, MFSD8, 5330417C22RIK, AKTIP, MYO7A, LYST, SYNJ1, TMEM165, RAB5A, RAB11A, CLN6 | 1.84.E-01 |
| *GO:0002064~epithelial cell development* | 13 | 1.78.E-03 | PARD3, ROCK1, MYO7A, DICER1, DNASE1L2, SCRIB, S1PR3, CDC42, FRMD6, MAP3K1, JMJD1C, MIR96, TMEM79 | 1.87.E-01 |
| *GO:0003383~apical constriction* | 3 | 1.78.E-03 | PARD3, FRMD6, ROCK1 | 1.84.E-01 |
| *GO:0007163~establishment or maintenance of cell polarity* | 11 | 1.85.E-03 | LAMA1, CDC42, PARD3, CAP2, ROCK1, CRB2, MCPH1, LMNA, GPSM2, FGF10, SCRIB | 1.85.E-01 |
| *GO:0030177~positive regulation of Wnt signaling pathway* | 8 | 2.09.E-03 | DIXDC1, SPIN1, RSPO1, FGF9, FGF10, NFKB1, FGF2, LRRK1 | 2.02.E-01 |
| *GO:0001942~hair follicle development* | 8 | 2.09.E-03 | CDC42, FGF7, BARX2, HDAC1, DICER1, FGF10, DNASE1L2, TMEM79 | 2.02.E-01 |
| *GO:0022404~molting cycle process* | 8 | 2.20.E-03 | CDC42, FGF7, BARX2, HDAC1, DICER1, FGF10, DNASE1L2, TMEM79 | 2.07.E-01 |
| *GO:0022405~hair cycle process* | 8 | 2.20.E-03 | CDC42, FGF7, BARX2, HDAC1, DICER1, FGF10, DNASE1L2, TMEM79 | 2.07.E-01 |
| *GO:0098773~skin epidermis development* | 8 | 2.33.E-03 | CDC42, FGF7, BARX2, HDAC1, DICER1, FGF10, DNASE1L2, TMEM79 | 2.13.E-01 |
| *GO:0001558~regulation of cell growth* | 17 | 2.42.E-03 | NOL8, CLSTN1, ADIPOR1, CDC42, CYBA, CTH, CDKN2A, HNF4A, PRDM4, NCOA3, FGFR1OP, SEMA3F, RAB11A, EFNA5, NEDD4L, 4932438A13RIK, DBN1 | 2.16.E-01 |
| *GO:0030324~lung development* | 12 | 2.56.E-03 | LAMA1, CDC42, FGF7, FGF9, JMJD6, ASXL1, DICER1, GPSM2, FGF10, SPARC, FGF2, PITX2 | 2.23.E-01 |
| *GO:0030323~respiratory tube development* | 12 | 2.83.E-03 | LAMA1, CDC42, FGF7, FGF9, JMJD6, ASXL1, DICER1, GPSM2, FGF10, SPARC, FGF2, PITX2 | 2.38.E-01 |
| *GO:0070252~actin-mediated cell contraction* | 6 | 2.91.E-03 | PARD3, FRMD6, ROCK1, DSC2, NEDD4L, DBN1 | 2.39.E-01 |
| *GO:0031069~hair follicle morphogenesis* | 5 | 3.13.E-03 | CDC42, FGF7, DICER1, FGF10, TMEM79 | 2.50.E-01 |
| *GO:0000902~cell morphogenesis* | 37 | 3.34.E-03 | BBS5, PARD3, SHROOM4, MYO7A, DICER1, PTPN23, PIP5K1C, DTNBP1, SCRIB, SDC2, CDC42, FRMD6, CRB2, SEMA3F, MAP3K1, RAB11A, NEDD4L, DIXDC1, CAP2, PLA2G10, ROCK1, KIF5A, CSNK1G2, ASXL1, TBCE, ADIPOR1, ANKRD27, LAMA1, MYO10, P2RX7, EFNA5, IFT81, EFNA4, MIR96, JMJD1C, ARAP3, DBN1 | 2.60.E-01 |
| *GO:0043583~ear development* | 12 | 3.41.E-03 | C1QTNF5, FGF9, MYO7A, MYO15, DICER1, FGF10, GRHL3, MAPKAPK2, SPARC, MIR96, SDC4, SCRIB | 2.60.E-01 |
| *GO:0006898~receptor-mediated endocytosis* | 11 | 3.46.E-03 | PARD3, PROM2, RSPO1, HIP1R, AAK1, SYNJ1, RAB5A, ACKR3, NEDD4L, DTNBP1, SCRIB | 2.58.E-01 |
| *GO:0043588~skin development* | 13 | 3.61.E-03 | FGF7, DICER1, FGF10, GRHL3, DNASE1L2, CDSN, TCF7L1, CDC42, BARX2, NCOA3, HDAC1, ASH1L, TMEM79 | 2.64.E-01 |
| *GO:1902680~positive regulation of RNA biosynthetic process* | 38 | 3.94.E-03 | FGF7, FGF9, DICER1, FGF10, NFKB1, FOXO3, ELK3, TCF7L1, PFN1, BARX2, CDKN2A, HSF1, RNF10, TCF4, PITX3, FGF2, MYSM1, PITX2, ASXL1, LMNA, RAF1, TEAD1, GRHL3, GTF2B, PLAC8, ARHGEF11, ELL2, DOT1L, ATF3, MTF1, NCOA3, HNF4A, HDAC1, ASH1L, NCOA6, AIRE, KDM8, PARP1 | 2.79.E-01 |
| *GO:1901361~organic cyclic compound catabolic process* | 14 | 4.16.E-03 | ALDH1L1, NBAS, DICER1, DNASE1L2, CANT1, CDKN2A, HSF1, DCP2, CYP7A1, GSPT2, SETMAR, AHCYL1, LSM1, AKR1D1 | 2.88.E-01 |
| *GO:0006357~regulation of transcription from RNA polymerase II promoter* | 47 | 4.20.E-03 | FGF9, YLPM1, DICER1, FGF10, CBX2, NFKB1, FOXO3, ELK3, TCF7L1, PFN1, EPC2, AHRR, CDKN2A, BARX2, HSF1, CRB2, RNF10, TCF4, PITX3, NFIL3, FGF2, MYSM1, PITX2, SATB1, TCF7, PKIG, RFX7, ASXL1, LMNA, RAF1, TEAD1, GRHL3, UCP1, PLAC8, ELL2, DOT1L, SARNP, ATF3, MTF1, HDAC1, HNF4A, NCOA3, NCOA6, ASH1L, AIRE, ZBTB4, PARP1 | 2.86.E-01 |
| *GO:0003382~epithelial cell morphogenesis* | 6 | 4.22.E-03 | PARD3, FRMD6, ROCK1, MAP3K1, JMJD1C, SCRIB | 2.83.E-01 |
| *GO:0036211~protein modification process* | 81 | 4.24.E-03 | FGF7, FGF9, DICER1, PTPN23, FGF10, SDC4, USP50, FANCL, MAP3K7, CDC42, EPC2, ST6GALNAC3, CDKN2A, RSPO1, HSF1, AAK1, SETMAR, RNF38, FGF2, SATB1, BRCC3, ZC3HC1, ROCK1, NOS1AP, CSNK1G2, HMBS, PKIG, PRKAB1, ACKR3, PTPRU, TMEM189, CTH, HNF4A, XXYLT1, MIB2, FGFR1OP, ASH1L, NCOA6, BMP2K, EFNA5, JMJD1C, LRRK1, LCP2, PARD3, ABI2, TRRAP, DUSP11, MAPKAPK2, DTNBP1, TIMP3, ZDHHC23, AHRR, MAP3K1, RNF10, NEDD4L, MYSM1, THPO, DIXDC1, RNF144B, KANSL1, ASXL1, RAF1, NDFIP2, TEAD1, CENPE, DOT1L, LAMA1, P2RX7, ATF3, PROM2, AKTIP, HDAC1, NXN, JMJD6, WSB2, TMEM165, KDM8, SETD6, SMURF2, SETD2, PARP1 | 2.80.E-01 |
| *GO:0006464~cellular protein modification process* | 81 | 4.24.E-03 | FGF7, FGF9, DICER1, PTPN23, FGF10, SDC4, USP50, FANCL, MAP3K7, CDC42, EPC2, ST6GALNAC3, CDKN2A, RSPO1, HSF1, AAK1, SETMAR, RNF38, FGF2, SATB1, BRCC3, ZC3HC1, ROCK1, NOS1AP, CSNK1G2, HMBS, PKIG, PRKAB1, ACKR3, PTPRU, TMEM189, CTH, HNF4A, XXYLT1, MIB2, FGFR1OP, ASH1L, NCOA6, BMP2K, EFNA5, JMJD1C, LRRK1, LCP2, PARD3, ABI2, TRRAP, DUSP11, MAPKAPK2, DTNBP1, TIMP3, ZDHHC23, AHRR, MAP3K1, RNF10, NEDD4L, MYSM1, THPO, DIXDC1, RNF144B, KANSL1, ASXL1, RAF1, NDFIP2, TEAD1, CENPE, DOT1L, LAMA1, P2RX7, ATF3, PROM2, AKTIP, HDAC1, NXN, JMJD6, WSB2, TMEM165, KDM8, SETD6, SMURF2, SETD2, PARP1 | 2.80.E-01 |
| *GO:0007399~nervous system development* | 57 | 4.25.E-03 | FGF9, MYO7A, SYNJ1, CLSTN1, DICER1, NCS1, PIP5K1C, FGF10, RPE65, FOXO3, SDC4, SDC2, MAP3K7, CDC42, ASPA, SEMA3F, LSM1, PITX3, FGF2, PITX2, RBFOX1, KIF5A, MPDZ, TBCE, GRHL3, SLC7A11, ANKRD27, NAB2, NCOA6, EFNA5, EFNA4, DBN1, PARD3, MFSD8, SHROOM4, ABI2, DTNBP1, ITM2C, SCRIB, PFN1, C1QTNF5, CRB2, DNER, RAB11A, RNF10, NEDD4L, TCF4, DIXDC1, TCF7, PLA2G10, PCDH18, LAMA1, HDAC1, RAB35, MCPH1, MIR96, SETD2 | 2.76.E-01 |
| *GO:0007010~cytoskeleton organization* | 33 | 4.28.E-03 | PARD3, FGF7, SHROOM4, PRC1, HIP1R, DICER1, ITGB5, ABI2, FGF10, PIP5K1C, SDC4, DTNBP1, CDC42, PFN1, FRMD6, SORBS2, MAP3K1, RAB11A, GPSM2, DIXDC1, CAP2, BAIAP2L2, ROCK1, LMNA, TBCE, RAF1, GRHL3, KNSTRN, LIMCH1, FGFR1OP, MCPH1, EFNA5, DBN1 | 2.73.E-01 |
| *GO:0051276~chromosome organization* | 32 | 4.29.E-03 | SPIN1, MAU2, DICER1, YLPM1, CBX2, TRRAP, PTMA, MAP3K7, SLX4, EPC2, CDKN2A, SETMAR, RAB11A, MYSM1, BAHD1, SATB1, BRCC3, KANSL1, ASXL1, CENPE, KNSTRN, DOT1L, HDAC1, JMJD6, MCPH1, ASH1L, NCOA6, KDM8, SETD6, JMJD1C, PARP1, SETD2 | 2.70.E-01 |
| *GO:0051254~positive regulation of RNA metabolic process* | 39 | 4.32.E-03 | FGF7, FGF9, DICER1, FGF10, NFKB1, FOXO3, ELK3, TCF7L1, PFN1, BARX2, CDKN2A, HSF1, RNF10, TCF4, PITX3, FGF2, MYSM1, PITX2, KHDRBS3, ASXL1, LMNA, RAF1, TEAD1, GRHL3, GTF2B, PLAC8, ARHGEF11, ELL2, DOT1L, ATF3, MTF1, NCOA3, HNF4A, HDAC1, ASH1L, NCOA6, AIRE, KDM8, PARP1 | 2.68.E-01 |
| *GO:0023056~positive regulation of signaling* | 41 | 4.67.E-03 | SPIN1, FGF9, HIP1R, CLSTN1, NCS1, FGF10, NFKB1, ITM2C, DTNBP1, TIMP3, SCRIB, CANT1, MAP3K7, CDC42, CDKN2A, HSF1, RSPO1, CRB2, AAK1, MAP3K1, FAM162A, FGF2, THPO, DIXDC1, DNM1L, ASXL1, ADIPOR1, NDFIP2, RAF1, ACKR3, CYBA, P2RX7, CTH, ATF3, HDAC1, HNF4A, NCOA3, MIB2, PARP1, DBN1, LRRK1 | 2.82.E-01 |
| *GO:0045935~positive regulation of nucleobase-containing compound metabolic process* | 44 | 4.71.E-03 | FGF7, FGF9, DICER1, FGF10, NFKB1, FOXO3, ELK3, TCF7L1, SLX4, PFN1, CDC42, BARX2, CDKN2A, HSF1, SETMAR, RNF10, TCF4, PITX3, FGF2, MYSM1, PITX2, KHDRBS3, BRCC3, ASXL1, LMNA, RAF1, TEAD1, GRHL3, GTF2B, PLAC8, ARHGEF11, ELL2, DOT1L, P2RX7, ATF3, MTF1, HDAC1, HNF4A, NCOA3, NCOA6, ASH1L, AIRE, KDM8, PARP1 | 2.80.E-01 |
| *GO:0018193~peptidyl-amino acid modification* | 30 | 4.80.E-03 | PARD3, FGF7, ABI2, FGF10, MAPKAPK2, TRRAP, MAP3K7, CDC42, EPC2, ST6GALNAC3, AHRR, CDKN2A, HSF1, SETMAR, KANSL1, NOS1AP, CSNK1G2, HMBS, RAF1, DOT1L, CTH, HNF4A, HDAC1, JMJD6, ASH1L, NCOA6, SETD6, EFNA5, SETD2, LRRK1 | 2.81.E-01 |
| *GO:2000026~regulation of multicellular organismal development* | 49 | 5.07.E-03 | PARD3, FGF7, FGF9, DICER1, SYNJ1, CLSTN1, NCS1, FGF10, FOXO3, ITM2C, DTNBP1, SCRIB, SDC2, PDCD2, CDC42, S1PR3, WARS, ASPA, CDKN2A, CRB2, SEMA3F, RAB11A, RNF10, NEDD4L, LSM1, PITX3, TCF4, FGF2, MYSM1, THPO, RBFOX1, DIXDC1, IL2RA, ROCK1, LMNA, ADIPOR1, GRHL3, SPARC, ANKRD27, LAMA1, CTH, P2RX7, HDAC1, NCOA3, HNF4A, BMP2K, EFNA5, SMURF2, DBN1 | 2.90.E-01 |
| *GO:0048730~epidermis morphogenesis* | 5 | 5.10.E-03 | CDC42, FGF7, DICER1, FGF10, TMEM79 | 2.88.E-01 |
| *GO:0006325~chromatin organization* | 24 | 5.62.E-03 | SATB1, SPIN1, BRCC3, KANSL1, DICER1, ASXL1, CBX2, TRRAP, PTMA, DOT1L, MAP3K7, EPC2, CDKN2A, HDAC1, JMJD6, ASH1L, SETMAR, NCOA6, SETD6, KDM8, JMJD1C, SETD2, MYSM1, BAHD1 | 3.08.E-01 |
| *GO:0009967~positive regulation of signal transduction* | 37 | 5.73.E-03 | SPIN1, FGF9, HIP1R, FGF10, NFKB1, ITM2C, TIMP3, CANT1, MAP3K7, CDC42, CDKN2A, HSF1, RSPO1, CRB2, AAK1, MAP3K1, FAM162A, FGF2, THPO, DIXDC1, DNM1L, ASXL1, ADIPOR1, NDFIP2, RAF1, ACKR3, CYBA, P2RX7, CTH, ATF3, NCOA3, HNF4A, HDAC1, MIB2, PARP1, DBN1, LRRK1 | 3.09.E-01 |
| *GO:0030048~actin filament-based movement* | 7 | 5.80.E-03 | PARD3, FRMD6, ROCK1, MYO7A, DSC2, NEDD4L, DBN1 | 3.09.E-01 |
| *GO:0051128~regulation of cellular component organization* | 59 | 6.03.E-03 | HIP1R, YLPM1, SYNJ1, CLSTN1, PTPN23, NCS1, SDC4, SDC2, MAP3K7, CDC42, CDKN2A, 5330417C22RIK, HSF1, RSPO1, AAK1, SEMA3F, SETMAR, ROCK1, CSNK1G2, NOL8, ADIPOR1, GRHL3, ANKRD27, CTH, HNF4A, NCOA3, PRDM4, FGFR1OP, RAB5A, EFNA5, DBN1, PARD3, ABI2, ITM2C, DTNBP1, SLX4, PFN1, CRB2, MAP3K1, RAB11A, FAM162A, NEDD4L, 4932438A13RIK, ELMOD1, DIXDC1, DNM1L, BAIAP2L2, LMNA, RAF1, CENPE, SPARC, CYBA, MYO10, P2RX7, PROM2, GRTP1, MCPH1, ARAP3, PARP1 | 3.15.E-01 |
| *GO:0090596~sensory organ morphogenesis* | 13 | 6.08.E-03 | FGF9, MYO7A, FGF10, GRHL3, RPE65, SCRIB, C1QTNF5, HDAC1, CRB2, MYO15, MIR96, PITX3, PITX2 | 3.13.E-01 |
| *GO:0032989~cellular component morphogenesis* | 38 | 6.23.E-03 | BBS5, PARD3, SHROOM4, MYO7A, DICER1, PTPN23, PIP5K1C, DTNBP1, SCRIB, SDC2, CDC42, FRMD6, CRB2, SEMA3F, MAP3K1, RAB11A, NEDD4L, DIXDC1, CAP2, DNM1L, PLA2G10, ROCK1, KIF5A, CSNK1G2, ASXL1, TBCE, ADIPOR1, ANKRD27, LAMA1, MYO10, P2RX7, EFNA5, IFT81, EFNA4, MIR96, JMJD1C, ARAP3, DBN1 | 3.16.E-01 |
| *GO:0060627~regulation of vesicle-mediated transport* | 17 | 6.30.E-03 | PARD3, HIP1R, SYNJ1, PTPN23, NCS1, SDC4, DTNBP1, ANKRD27, CYBA, PROM2, RSPO1, AAK1, GRTP1, RAB5A, RAB11A, YIPF5, NEDD4L | 3.15.E-01 |
| *GO:1903508~positive regulation of nucleic acid-templated transcription* | 37 | 6.47.E-03 | FGF7, FGF9, DICER1, FGF10, NFKB1, FOXO3, ELK3, TCF7L1, PFN1, CDKN2A, BARX2, HSF1, RNF10, TCF4, PITX3, FGF2, MYSM1, PITX2, ASXL1, LMNA, TEAD1, RAF1, GRHL3, PLAC8, ARHGEF11, ELL2, DOT1L, ATF3, MTF1, NCOA3, HNF4A, HDAC1, ASH1L, NCOA6, AIRE, KDM8, PARP1 | 3.19.E-01 |
| *GO:0045893~positive regulation of transcription, DNA-templated* | 37 | 6.47.E-03 | FGF7, FGF9, DICER1, FGF10, NFKB1, FOXO3, ELK3, TCF7L1, PFN1, CDKN2A, BARX2, HSF1, RNF10, TCF4, PITX3, FGF2, MYSM1, PITX2, ASXL1, LMNA, TEAD1, RAF1, GRHL3, PLAC8, ARHGEF11, ELL2, DOT1L, ATF3, MTF1, NCOA3, HNF4A, HDAC1, ASH1L, NCOA6, AIRE, KDM8, PARP1 | 3.19.E-01 |
| *GO:0007267~cell-cell signaling* | 36 | 6.66.E-03 | GJA8, SPIN1, YWHAZ, FGF7, FGF9, CLSTN1, SYNJ1, NCS1, FGF10, NFKB1, FOXO3, DTNBP1, TCF7L1, SCRIB, CDC42, RSPO1, RAB11A, FGF2, PITX2, DIXDC1, TCF7, CSNK1G2, HTR4, RAF1, GRHL3, PTPRU, GRM3, P2RX7, NXN, HNF4A, HDAC1, GRM6, RAB5A, EFNA5, DBN1, LRRK1 | 3.23.E-01 |
| *GO:0060541~respiratory system development* | 12 | 6.75.E-03 | LAMA1, CDC42, FGF7, FGF9, JMJD6, ASXL1, DICER1, GPSM2, FGF10, SPARC, FGF2, PITX2 | 3.23.E-01 |
| *GO:0098602~single organism cell adhesion* | 25 | 6.86.E-03 | ADAMTS18, PARD3, DICER1, PIP5K1C, ITGB5, SDC4, CDSN, SCRIB, CDC42, CDKN2A, SATB1, TCF7, IL2RA, ROCK1, PTPRU, SLC7A11, RIC8A, ARVCF, P2RX7, MYO10, JMJD6, AIRE, DSC2, EFNA5, DBN1 | 3.24.E-01 |
| *GO:0006796~phosphate-containing compound metabolic process* | 68 | 6.87.E-03 | FGF7, FGF9, DICER1, SYNJ1, PTPN23, PIP5K1C, FGF10, SDC4, CANT1, MAP3K7, CDC42, CDKN2A, HSF1, RSPO1, PGAM5, AAK1, ATP5S, FGF2, NDUFS1, ROCK1, CSNK1G2, PKIG, PRKAB1, ACKR3, PTPRU, FBP2, HNF4A, LYST, FGFR1OP, ASH1L, BMP2K, ADSL, EFNA5, MVK, PLA2G2E, LRRK1, LCP2, UQCRB, PARD3, FDX1, ABI2, DUSP11, MAPKAPK2, TIMP3, DTNBP1, GALK2, MAP3K1, GGPS1, PDHX, ACSL5, THPO, NADK2, DIXDC1, CAP2, DNM1L, PLA2G10, RAF1, TEAD1, CENPE, LAMA1, P2RX7, ATF3, PROM2, AKTIP, HDAC1, MCPH1, FABP3, PARP1 | 3.21.E-01 |
| *GO:0060429~epithelium development* | 33 | 6.88.E-03 | PARD3, FGF7, MYO7A, DICER1, FGF10, DNASE1L2, SDC4, SCRIB, MAP3K7, PFN1, CDC42, S1PR3, BARX2, CDKN2A, FRMD6, CRB2, MAP3K1, GPSM2, PITX3, FGF2, TMEM79, PITX2, TCF7, ROCK1, TEAD1, GRHL3, SLC7A11, LAMA1, HDAC1, NCOA3, MIR96, JMJD1C, SETD2 | 3.18.E-01 |
| *GO:0008104~protein localization* | 58 | 7.04.E-03 | FGF7, FGF9, PLEKHM1, MYO7A, PTPN23, PIP5K1C, FGF10, VPS37D, PEX3, CDC42, FRMD6, AGAP1, DUOXA2, ROCK1, KIF5A, PKIG, PTPRU, ANKRD27, ARHGAP33, HNF4A, LYST, RAB5A, EFNA5, IFT81, DBN1, LCP2, ARL6IP1, PARD3, YWHAZ, BBS5, SCRIB, PFN1, ZDHHC23, C1QTNF5, RAB11A, NEDD4L, TNPO2, ELMOD1, FYB, GDI2, DNM1L, RHBDF1, LMNA, TOMM40, NDFIP2, RAF1, CENPE, P2RX7, AKTIP, HDAC1, ARF3, RAB35, GRTP1, MCPH1, YIPF5, NUTF2, AHCYL1, PARP1 | 3.20.E-01 |
| *GO:0006793~phosphorus metabolic process* | 68 | 7.09.E-03 | FGF7, FGF9, DICER1, SYNJ1, PTPN23, PIP5K1C, FGF10, SDC4, CANT1, MAP3K7, CDC42, CDKN2A, HSF1, RSPO1, PGAM5, AAK1, ATP5S, FGF2, NDUFS1, ROCK1, CSNK1G2, PKIG, PRKAB1, ACKR3, PTPRU, FBP2, HNF4A, LYST, FGFR1OP, ASH1L, BMP2K, ADSL, EFNA5, MVK, PLA2G2E, LRRK1, LCP2, UQCRB, PARD3, FDX1, ABI2, DUSP11, MAPKAPK2, TIMP3, DTNBP1, GALK2, MAP3K1, GGPS1, PDHX, ACSL5, THPO, NADK2, DIXDC1, CAP2, DNM1L, PLA2G10, RAF1, TEAD1, CENPE, LAMA1, P2RX7, ATF3, PROM2, AKTIP, HDAC1, MCPH1, FABP3, PARP1 | 3.19.E-01 |
| *GO:0051094~positive regulation of developmental process* | 35 | 7.20.E-03 | PARD3, FGF7, FGF9, CLSTN1, SYNJ1, DICER1, FGF10, FOXO3, PDCD2, CDC42, ASPA, CDKN2A, HSF1, CRB2, RAB11A, RNF10, NEDD4L, TCF4, PITX3, FGF2, TMEM79, THPO, DIXDC1, IL2RA, DNM1L, LMNA, TEAD1, ANKRD27, P2RX7, CTH, NCOA3, HDAC1, EFNA5, SMURF2, DBN1 | 3.20.E-01 |
| *GO:0016578~histone deubiquitination* | 4 | 7.22.E-03 | BRCC3, ASXL1, TRRAP, MYSM1 | 3.17.E-01 |
| *GO:0010647~positive regulation of cell communication* | 40 | 7.27.E-03 | SPIN1, FGF9, HIP1R, CLSTN1, NCS1, FGF10, NFKB1, ITM2C, DTNBP1, TIMP3, CANT1, MAP3K7, CDC42, CDKN2A, HSF1, RSPO1, CRB2, AAK1, MAP3K1, FAM162A, FGF2, THPO, DIXDC1, DNM1L, ASXL1, ADIPOR1, NDFIP2, RAF1, ACKR3, CYBA, P2RX7, CTH, ATF3, HDAC1, HNF4A, NCOA3, MIB2, PARP1, DBN1, LRRK1 | 3.16.E-01 |
| *GO:0000904~cell morphogenesis involved in differentiation* | 25 | 7.56.E-03 | DIXDC1, PARD3, ROCK1, PLA2G10, KIF5A, MYO7A, TBCE, ADIPOR1, PIP5K1C, DTNBP1, SDC2, SCRIB, ANKRD27, LAMA1, CDC42, FRMD6, CRB2, MAP3K1, SEMA3F, RAB11A, EFNA5, EFNA4, MIR96, JMJD1C, DBN1 | 3.23.E-01 |
| *GO:2001233~regulation of apoptotic signaling pathway* | 15 | 7.57.E-03 | ZC3HC1, DNM1L, HIP1R, LMNA, FGF10, RAF1, ACKR3, TIMP3, ITM2C, MAP3K7, CTH, ATF3, HDAC1, FAM162A, PARP1 | 3.20.E-01 |
| *GO:0060348~bone development* | 10 | 7.71.E-03 | SPNS2, HSD17B1, NAB2, ANKRD11, MCPH1, ASXL1, SLC38A10, SPARC, LRRK1, PITX2 | 3.22.E-01 |
| *GO:1903793~positive regulation of anion transport* | 5 | 7.76.E-03 | ARL6IP1, P2RX7, PLA2G10, DTNBP1, ACSL5 | 3.21.E-01 |
| *GO:0032970~regulation of actin filament-based process* | 14 | 7.93.E-03 | DIXDC1, PARD3, ROCK1, BAIAP2L2, HIP1R, ABI2, GRHL3, SDC4, PFN1, FRMD6, MAP3K1, DSC2, EFNA5, DBN1 | 3.23.E-01 |
| *GO:0030855~epithelial cell differentiation* | 21 | 8.08.E-03 | PARD3, ROCK1, MYO7A, DICER1, FGF10, DNASE1L2, SLC7A11, SCRIB, LAMA1, S1PR3, CDC42, FRMD6, NCOA3, HDAC1, MAP3K1, GPSM2, MIR96, JMJD1C, PITX3, FGF2, TMEM79 | 3.25.E-01 |
| *GO:0051301~cell division* | 19 | 8.92.E-03 | DIXDC1, PARD3, FGF7, BRCC3, ZC3HC1, PRC1, MAU2, FGF9, DICER1, CENPE, KNSTRN, RAB11FIP4, CDC42, CDKN2A, NCOA3, RAB35, RAB11A, FGF2, NSUN2 | 3.49.E-01 |
| *GO:0010628~positive regulation of gene expression* | 44 | 9.32.E-03 | FGF7, FGF9, DICER1, FGF10, NFKB1, FOXO3, ELK3, DTNBP1, TCF7L1, PFN1, CDC42, BARX2, CDKN2A, HSF1, RNF10, TCF4, PITX3, NFIL3, FGF2, MYSM1, PITX2, KHDRBS3, ASXL1, PRKAB1, LMNA, RAF1, TEAD1, GRHL3, GTF2B, PLAC8, ARHGEF11, ELL2, DOT1L, P2RX7, ATF3, MTF1, HDAC1, HNF4A, NCOA3, NCOA6, ASH1L, AIRE, KDM8, PARP1 | 3.59.E-01 |
| *GO:0051640~organelle localization* | 16 | 9.50.E-03 | YWHAZ, PARD3, DNM1L, MYO7A, SYNJ1, FGF10, CENPE, DTNBP1, SCRIB, CDC42, P2RX7, MCPH1, RAB5A, RAB11A, GPSM2, 4932438A13RIK | 3.61.E-01 |
| *GO:0040008~regulation of growth* | 22 | 9.74.E-03 | FGF9, NOL8, CLSTN1, ADIPOR1, PLAC8, CDC42, CYBA, CTH, CDKN2A, HSF1, HNF4A, PRDM4, NCOA3, FGFR1OP, SEMA3F, RAB11A, EFNA5, NEDD4L, 4932438A13RIK, PARP1, DBN1, FGF2 | 3.65.E-01 |
| *GO:0045927~positive regulation of growth* | 12 | 9.77.E-03 | CYBA, CDC42, HSF1, NCOA3, FGF9, FGFR1OP, NOL8, RAB11A, EFNA5, NEDD4L, FGF2, DBN1 | 3.63.E-01 |
| *GO:0009611~response to wounding* | 18 | 1.01.E-02 | ADAMTS18, PARD3, FGF7, DICER1, PIP5K1C, FGF10, GRHL3, SPARC, ELK3, SDC4, DTNBP1, SLC7A11, SCRIB, GP6, HNF4A, MAP3K1, LYST, FGF2 | 3.69.E-01 |
| *GO:0010452~histone H3-K36 methylation* | 3 | 1.01.E-02 | ASH1L, SETMAR, SETD2 | 3.67.E-01 |
| *GO:0051641~cellular localization* | 56 | 1.01.E-02 | FGF7, FGF9, MYO7A, SYNJ1, PTPN23, PIP5K1C, FGF10, PEX3, CDC42, CDKN2A, FRMD6, FGF2, ROCK1, KIF5A, PKIG, PTPRU, SARNP, ANKRD27, HNF4A, LYST, RAB5A, EFNA5, IFT81, DBN1, ARL6IP1, PARD3, YWHAZ, BBS5, DTNBP1, SCRIB, PFN1, ZDHHC23, GPSM2, RAB11A, NEDD4L, TNPO2, 4932438A13RIK, ELMOD1, FYB, DNM1L, LMNA, TOMM40, RAF1, CENPE, CYBA, P2RX7, MYO10, AKTIP, RAB35, GRTP1, MCPH1, YIPF5, NUTF2, AHCYL1, PARP1, SETD2 | 3.65.E-01 |
| *GO:0006351~transcription, DNA-templated* | 72 | 1.04.E-02 | SPIN1, FGF7, FGF9, DICER1, YLPM1, FGF10, CBX2, NFKB1, FOXO3, USP50, MAP3K7, EPC2, BARX2, CDKN2A, HSF1, NFIL3, PITX3, FGF2, PITX2, SATB1, KHDRBS3, PKIG, GRHL3, GTF2B, ZBTB24, ARHGEF11, ELL2, SARNP, CTH, PRDM4, HNF4A, ASCC2, MTF1, NCOA3, NAB2, ASH1L, NCOA6, VOPP1, JMJD1C, ELK3, TRRAP, TCF7L1, PFN1, AHRR, CRB2, HSF2BP, RNF10, TCF4, MYSM1, BAHD1, TCF7, PLA2G10, GMNN, PRIMPOL, ASXL1, RFX7, LMNA, RAF1, TEAD1, UCP1, PLAC8, DOT1L, ATF3, HDAC1, JMJD6, ZFP182, AIRE, KDM8, ZBTB4, SETD6, SETD2, PARP1 | 3.69.E-01 |
| *GO:0016337~single organismal cell-cell adhesion* | 23 | 1.06.E-02 | ADAMTS18, SATB1, TCF7, PARD3, IL2RA, ROCK1, DICER1, PIP5K1C, ITGB5, PTPRU, SDC4, CDSN, SLC7A11, SCRIB, RIC8A, ARVCF, CDC42, P2RX7, MYO10, CDKN2A, JMJD6, AIRE, DSC2 | 3.73.E-01 |
| *GO:0051649~establishment of localization in cell* | 44 | 1.08.E-02 | ARL6IP1, BBS5, PARD3, YWHAZ, FGF9, MYO7A, SYNJ1, PTPN23, FGF10, PEX3, DTNBP1, SCRIB, CDC42, CDKN2A, RAB11A, GPSM2, TNPO2, FGF2, ELMOD1, KIF5A, PKIG, LMNA, TOMM40, RAF1, CENPE, SARNP, ANKRD27, CYBA, MYO10, P2RX7, AKTIP, HNF4A, RAB35, LYST, GRTP1, MCPH1, RAB5A, NUTF2, YIPF5, IFT81, AHCYL1, SETD2, PARP1, DBN1 | 3.73.E-01 |
| *GO:0030307~positive regulation of cell growth* | 9 | 1.09.E-02 | CYBA, CDC42, NCOA3, FGFR1OP, NOL8, RAB11A, EFNA5, NEDD4L, DBN1 | 3.74.E-01 |
| *GO:0030111~regulation of Wnt signaling pathway* | 11 | 1.13.E-02 | DIXDC1, SPIN1, NXN, RSPO1, HDAC1, FGF9, FGF10, NFKB1, FOXO3, FGF2, LRRK1 | 3.81.E-01 |
| *GO:0008637~apoptotic mitochondrial changes* | 7 | 1.17.E-02 | DNM1L, CDKN2A, HIP1R, MAP3K1, LMNA, FAM162A, NDUFS1 | 3.91.E-01 |
| *GO:0048468~cell development* | 55 | 1.18.E-02 | MYO7A, SYNJ1, DICER1, NCS1, PIP5K1C, RPE65, FOXO3, DNASE1L2, SDC4, SDC2, S1PR3, CDC42, ASPA, FRMD6, SEMA3F, LSM1, PITX3, FGF2, PITX2, ROCK1, KIF5A, TBCE, ADIPOR1, ANKRD27, EFNA5, EFNA4, JMJD1C, DBN1, LRRK1, PARD3, MFSD8, ABI2, DTNBP1, ITM2C, SCRIB, C1QTNF5, CRB2, SORBS2, MAP3K1, DNER, RAB11A, RNF10, NEDD4L, TCF4, NSUN2, TMEM79, DIXDC1, PLA2G10, LMNA, LAMA1, HDAC1, JMJD6, RAB35, MIR96, SETD2 | 3.89.E-01 |
| *GO:0022008~neurogenesis* | 42 | 1.19.E-02 | PARD3, MFSD8, MYO7A, SYNJ1, DICER1, NCS1, ABI2, FGF10, PIP5K1C, RPE65, FOXO3, SDC4, ITM2C, DTNBP1, SCRIB, SDC2, CDC42, ASPA, C1QTNF5, SEMA3F, DNER, RAB11A, RNF10, NEDD4L, TCF4, LSM1, PITX3, FGF2, PITX2, DIXDC1, PLA2G10, KIF5A, TBCE, ANKRD27, LAMA1, HDAC1, NAB2, RAB35, EFNA5, EFNA4, MIR96, DBN1 | 3.89.E-01 |
| *GO:0035295~tube development* | 22 | 1.19.E-02 | TCF7, FGF7, FGF9, DICER1, ASXL1, TEAD1, FGF10, GRHL3, SPARC, SDC4, SCRIB, MAP3K7, LAMA1, CDC42, PFN1, NCOA3, JMJD6, GPSM2, JMJD1C, SETD2, FGF2, PITX2 | 3.87.E-01 |
| *GO:0015909~long-chain fatty acid transport* | 5 | 1.20.E-02 | PLA2G10, ABCD1, FABP3, PLA2G2E, ACSL5 | 3.86.E-01 |
| *GO:0051674~localization of cell* | 36 | 1.21.E-02 | PARD3, FGF7, PTPN23, ABI2, FGF10, SDC4, SCRIB, SDC2, CDC42, PFN1, SORBS2, SEMA3F, MAP3K1, RAB11A, FGF2, MYSM1, PITX2, DIXDC1, SPNS2, ROCK1, RHBDF1, LMNA, ADIPOR1, SPARC, PTPRU, ELMO3, RIC8A, LAMA1, MYO10, FGFR1OP, LYST, RAB5A, SMURF2, ARAP3, SETD2, GRB7 | 3.85.E-01 |
| *GO:0048870~cell motility* | 36 | 1.21.E-02 | PARD3, FGF7, PTPN23, ABI2, FGF10, SDC4, SCRIB, SDC2, CDC42, PFN1, SORBS2, SEMA3F, MAP3K1, RAB11A, FGF2, MYSM1, PITX2, DIXDC1, SPNS2, ROCK1, RHBDF1, LMNA, ADIPOR1, SPARC, PTPRU, ELMO3, RIC8A, LAMA1, MYO10, FGFR1OP, LYST, RAB5A, SMURF2, ARAP3, SETD2, GRB7 | 3.85.E-01 |
| *GO:0046700~heterocycle catabolic process* | 12 | 1.22.E-02 | ALDH1L1, CDKN2A, NBAS, HSF1, DCP2, SETMAR, GSPT2, DICER1, AHCYL1, LSM1, DNASE1L2, CANT1 | 3.85.E-01 |
| *GO:0042063~gliogenesis* | 12 | 1.22.E-02 | ASPA, PARD3, HDAC1, DNER, NAB2, SYNJ1, DICER1, FGF10, RNF10, PITX3, FGF2, SCRIB | 3.85.E-01 |
| *GO:0055091~phospholipid homeostasis* | 3 | 1.25.E-02 | HNF4A, LYST, FABP3 | 3.89.E-01 |
| *GO:0044270~cellular nitrogen compound catabolic process* | 12 | 1.25.E-02 | ALDH1L1, CDKN2A, NBAS, HSF1, DCP2, SETMAR, GSPT2, DICER1, AHCYL1, LSM1, DNASE1L2, CANT1 | 3.87.E-01 |
| *GO:0060828~regulation of canonical Wnt signaling pathway* | 9 | 1.27.E-02 | DIXDC1, RSPO1, HDAC1, FGF9, FGF10, NFKB1, FOXO3, FGF2, LRRK1 | 3.90.E-01 |
| *GO:0042472~inner ear morphogenesis* | 7 | 1.28.E-02 | FGF9, MYO7A, MYO15, FGF10, GRHL3, MIR96, SCRIB | 3.88.E-01 |
| *GO:0051173~positive regulation of nitrogen compound metabolic process* | 44 | 1.28.E-02 | FGF7, FGF9, DICER1, FGF10, NFKB1, FOXO3, ELK3, TCF7L1, SLX4, PFN1, CDC42, BARX2, CDKN2A, HSF1, SETMAR, RNF10, TCF4, PITX3, FGF2, MYSM1, PITX2, KHDRBS3, BRCC3, ASXL1, LMNA, RAF1, TEAD1, GRHL3, GTF2B, PLAC8, ARHGEF11, ELL2, DOT1L, P2RX7, ATF3, MTF1, HDAC1, HNF4A, NCOA3, NCOA6, ASH1L, AIRE, KDM8, PARP1 | 3.86.E-01 |
| *GO:0046942~carboxylic acid transport* | 11 | 1.28.E-02 | ARL6IP1, P2RX7, PLA2G10, ABCB11, ABCD1, FABP3, SLC38A10, PLA2G2E, DTNBP1, SLC7A11, ACSL5 | 3.84.E-01 |
| *GO:0009891~positive regulation of biosynthetic process* | 44 | 1.31.E-02 | FGF7, FGF9, DICER1, FGF10, NFKB1, MAPKAPK2, FOXO3, ELK3, TCF7L1, CDC42, PFN1, BARX2, CDKN2A, HSF1, CYP7A1, RNF10, TCF4, PITX3, FGF2, MYSM1, ACSL5, PITX2, ASXL1, LMNA, RAF1, TEAD1, GRHL3, GTF2B, PLAC8, ARHGEF11, ELL2, DOT1L, CYBA, ATF3, MTF1, HDAC1, HNF4A, NCOA3, ASH1L, NCOA6, AIRE, FABP3, KDM8, PARP1 | 3.88.E-01 |
| *GO:0006066~alcohol metabolic process* | 11 | 1.32.E-02 | FDX1, CYP7A1, SYNJ1, ALDH2, NFKB1, LSS, MVK, RPE65, AKR1D1, FGF2, CLN6 | 3.87.E-01 |
| *GO:0060119~inner ear receptor cell development* | 5 | 1.37.E-02 | MYO7A, DICER1, MIR96, SDC4, SCRIB | 3.96.E-01 |
| *GO:0045022~early endosome to late endosome transport* | 4 | 1.38.E-02 | ANKRD27, AKTIP, RAB5A, PTPN23 | 3.96.E-01 |
| *GO:0002009~morphogenesis of an epithelium* | 18 | 1.40.E-02 | PARD3, FGF7, DICER1, TEAD1, FGF10, GRHL3, SDC4, SCRIB, MAP3K7, LAMA1, CDC42, PFN1, NCOA3, MAP3K1, SETD2, FGF2, TMEM79, PITX2 | 3.98.E-01 |
| *GO:0034655~nucleobase-containing compound catabolic process* | 11 | 1.42.E-02 | CDKN2A, NBAS, HSF1, DCP2, SETMAR, GSPT2, DICER1, AHCYL1, LSM1, DNASE1L2, CANT1 | 4.00.E-01 |
| *GO:0097190~apoptotic signaling pathway* | 19 | 1.42.E-02 | SIVA1, DNM1L, ZC3HC1, HIP1R, LMNA, FGF10, RAF1, ACKR3, FOXO3, TIMP3, ITM2C, ANXA6, MAP3K7, CTH, P2RX7, ATF3, HDAC1, FAM162A, PARP1 | 3.97.E-01 |
| *GO:0065009~regulation of molecular function* | 53 | 1.43.E-02 | HIP1R, FGF10, NFKB1, SDC4, PDCD2, MAP3K7, CDC42, CDKN2A, HSF1, PGAM5, SETMAR, FGF2, PITX2, NOS1AP, ROCK1, PKIG, PRKAB1, GRHL3, ANKRD27, CTH, HNF4A, NCOA3, FGFR1OP, EFNA5, DBN1, LCP2, ARL6IP1, FDX1, TIMP3, DTNBP1, SCRIB, PFN1, CRB2, MAP3K1, FAM162A, NEDD4L, ELMOD1, CAP2, PLA2G10, NDFIP2, TEAD1, RAF1, CENPE, DOT1L, CYBA, P2RX7, PROM2, AKTIP, GRTP1, MCPH1, SETD6, AHCYL1, PARP1 | 3.96.E-01 |
| *GO:0040007~growth* | 29 | 1.45.E-02 | FGF7, FGF9, CLSTN1, DICER1, FGF10, FOXO3, CDC42, CDKN2A, HSF1, SORBS2, SEMA3F, ANKRD11, RAB11A, NEDD4L, 4932438A13RIK, FGF2, NOL8, TBCE, ADIPOR1, PLAC8, CYBA, CTH, NCOA3, HNF4A, PRDM4, FGFR1OP, EFNA5, PARP1, DBN1 | 3.98.E-01 |
| *GO:0001501~skeletal system development* | 17 | 1.48.E-02 | SPNS2, FGF9, HSD17B1, DICER1, ASXL1, SLC38A10, SPARC, P2RX7, BARX2, NAB2, ASH1L, MCPH1, ANKRD11, SETD2, LRRK1, FGF2, PITX2 | 4.03.E-01 |
| *GO:0031328~positive regulation of cellular biosynthetic process* | 43 | 1.51.E-02 | FGF7, FGF9, DICER1, FGF10, NFKB1, MAPKAPK2, FOXO3, ELK3, TCF7L1, CDC42, PFN1, BARX2, CDKN2A, HSF1, CYP7A1, RNF10, TCF4, PITX3, FGF2, MYSM1, ACSL5, PITX2, ASXL1, LMNA, RAF1, TEAD1, GRHL3, GTF2B, PLAC8, ARHGEF11, ELL2, DOT1L, ATF3, MTF1, HDAC1, NCOA3, HNF4A, ASH1L, NCOA6, AIRE, FABP3, KDM8, PARP1 | 4.06.E-01 |
| *GO:0015908~fatty acid transport* | 6 | 1.51.E-02 | P2RX7, PLA2G10, ABCD1, FABP3, PLA2G2E, ACSL5 | 4.04.E-01 |
| *GO:0048260~positive regulation of receptor-mediated endocytosis* | 5 | 1.55.E-02 | PARD3, HIP1R, SYNJ1, NEDD4L, DTNBP1 | 4.10.E-01 |
| *GO:0007015~actin filament organization* | 13 | 1.57.E-02 | CDC42, PFN1, CAP2, BAIAP2L2, SHROOM4, ROCK1, SORBS2, HIP1R, MAP3K1, ABI2, ITGB5, SDC4, DBN1 | 4.11.E-01 |
| *GO:0030100~regulation of endocytosis* | 10 | 1.60.E-02 | CYBA, PARD3, PROM2, RSPO1, HIP1R, AAK1, SYNJ1, RAB5A, NEDD4L, DTNBP1 | 4.14.E-01 |
| *GO:0019439~aromatic compound catabolic process* | 12 | 1.61.E-02 | ALDH1L1, CDKN2A, NBAS, HSF1, DCP2, SETMAR, GSPT2, DICER1, AHCYL1, LSM1, DNASE1L2, CANT1 | 4.15.E-01 |
| *GO:0051247~positive regulation of protein metabolic process* | 37 | 1.66.E-02 | FGF7, FGF9, HIP1R, FGF10, MAPKAPK2, SDC4, DTNBP1, PDCD2, MAP3K7, CDC42, AHRR, CDKN2A, HSF1, RSPO1, MAP3K1, FAM162A, NEDD4L, FGF2, THPO, RNF144B, DIXDC1, NOS1AP, PLA2G10, TEAD1, RAF1, NDFIP2, CENPE, ACKR3, P2RX7, PROM2, AKTIP, HNF4A, HDAC1, EFNA5, LRRK1, CLN6, LCP2 | 4.22.E-01 |
| *GO:0032270~positive regulation of cellular protein metabolic process* | 35 | 1.71.E-02 | FGF7, FGF9, HIP1R, FGF10, SDC4, DTNBP1, PDCD2, MAP3K7, CDC42, AHRR, CDKN2A, HSF1, RSPO1, MAP3K1, FAM162A, FGF2, THPO, RNF144B, DIXDC1, NOS1AP, PLA2G10, TEAD1, RAF1, NDFIP2, CENPE, ACKR3, P2RX7, PROM2, HNF4A, HDAC1, AKTIP, EFNA5, LRRK1, CLN6, LCP2 | 4.30.E-01 |
| *GO:0016049~cell growth* | 16 | 1.72.E-02 | NOL8, ADIPOR1, CDC42, CYBA, CTH, CDKN2A, HNF4A, PRDM4, NCOA3, SORBS2, FGFR1OP, SEMA3F, RAB11A, EFNA5, NEDD4L, DBN1 | 4.29.E-01 |
| *GO:0044070~regulation of anion transport* | 6 | 1.74.E-02 | ARL6IP1, P2RX7, PLA2G10, AHCYL1, DTNBP1, ACSL5 | 4.30.E-01 |
| *GO:0015718~monocarboxylic acid transport* | 7 | 1.75.E-02 | P2RX7, PLA2G10, ABCB11, ABCD1, FABP3, PLA2G2E, ACSL5 | 4.30.E-01 |
| *GO:0030010~establishment of cell polarity* | 7 | 1.75.E-02 | LAMA1, PARD3, MCPH1, LMNA, GPSM2, FGF10, SCRIB | 4.30.E-01 |
| *GO:0010557~positive regulation of macromolecule biosynthetic process* | 40 | 1.76.E-02 | FGF7, FGF9, DICER1, FGF10, NFKB1, MAPKAPK2, FOXO3, ELK3, TCF7L1, CDC42, PFN1, BARX2, CDKN2A, HSF1, RNF10, TCF4, PITX3, FGF2, MYSM1, PITX2, ASXL1, LMNA, RAF1, TEAD1, GRHL3, GTF2B, PLAC8, ARHGEF11, ELL2, DOT1L, ATF3, MTF1, HDAC1, NCOA3, HNF4A, ASH1L, NCOA6, AIRE, KDM8, PARP1 | 4.28.E-01 |
| *GO:0043933~macromolecular complex subunit organization* | 53 | 1.78.E-02 | GJA8, SPIN1, HIP1R, HBS1L, SYNJ1, DICER1, CBX2, PTMA, MAP3K7, CDC42, EPC2, CDKN2A, SETMAR, SATB1, BRCC3, PRKAB1, TBCE, ANKRD27, CTH, NCOA6, ASH1L, ADSL, JMJD1C, PLA2G2E, DBN1, UQCRB, ABI2, TRRAP, ANXA6, PFN1, C1QTNF5, MAP3K1, TCF4, TNPO2, MYSM1, BAHD1, DNM1L, BAIAP2L2, KANSL1, GMNN, ASXL1, RAF1, DOT1L, CYBA, P2RX7, HDAC1, JMJD6, GSPT2, KDM8, SETD6, PARP1, SETD2, GRB7 | 4.30.E-01 |
| *GO:0051988~regulation of attachment of spindle microtubules to kinetochore* | 3 | 1.79.E-02 | CDC42, CENPE, KNSTRN | 4.29.E-01 |
| *GO:0098927~vesicle-mediated transport between endosomal compartments* | 4 | 1.80.E-02 | ANKRD27, AKTIP, RAB5A, PTPN23 | 4.29.E-01 |
| *GO:0098609~cell-cell adhesion* | 30 | 1.83.E-02 | ADAMTS18, YWHAZ, PARD3, CLSTN1, DICER1, PTPN23, PIP5K1C, ITGB5, SDC4, CDSN, SCRIB, CDC42, CDKN2A, SATB1, TCF7, IL2RA, ROCK1, PCDH12, PTPRU, SLC7A11, PCDH18, RIC8A, ARVCF, DSG1B, P2RX7, MYO10, JMJD6, AIRE, DSC2, CDH10 | 4.33.E-01 |
| *GO:0071363~cellular response to growth factor stimulus* | 19 | 1.85.E-02 | FGF7, FGF9, ITGB5, FGF10, RAF1, NFKB1, MAPKAPK2, SPARC, FOXO3, MAP3K7, PFN1, CRB2, MAP3K1, RAB35, SMURF2, TCF4, PARP1, FGF2, DBN1 | 4.34.E-01 |
| *GO:0015031~protein transport* | 43 | 1.85.E-02 | ARL6IP1, YWHAZ, PARD3, BBS5, FGF9, PLEKHM1, MYO7A, PTPN23, VPS37D, PEX3, CDC42, C1QTNF5, RAB11A, TNPO2, AGAP1, ELMOD1, GDI2, DNM1L, DUOXA2, RHBDF1, PKIG, LMNA, TOMM40, NDFIP2, RAF1, ANKRD27, P2RX7, ARHGAP33, AKTIP, HDAC1, HNF4A, ARF3, RAB35, LYST, GRTP1, RAB5A, EFNA5, NUTF2, YIPF5, IFT81, AHCYL1, PARP1, LCP2 | 4.31.E-01 |
| *GO:0030036~actin cytoskeleton organization* | 18 | 1.90.E-02 | FGF7, CAP2, ROCK1, SHROOM4, BAIAP2L2, HIP1R, ITGB5, FGF10, ABI2, SDC4, DTNBP1, PFN1, CDC42, FRMD6, SORBS2, LIMCH1, MAP3K1, DBN1 | 4.37.E-01 |
| *GO:0048729~tissue morphogenesis* | 20 | 1.93.E-02 | PARD3, FGF7, DICER1, TEAD1, FGF10, GRHL3, SDC4, TCF7L1, SCRIB, MAP3K7, LAMA1, CDC42, PFN1, NCOA3, CRB2, MAP3K1, SETD2, FGF2, TMEM79, PITX2 | 4.40.E-01 |
| *GO:0043085~positive regulation of catalytic activity* | 27 | 1.93.E-02 | HIP1R, FDX1, FGF10, SDC4, SCRIB, PDCD2, MAP3K7, CDC42, PFN1, CDKN2A, HSF1, PGAM5, MAP3K1, SETMAR, FAM162A, FGF2, ELMOD1, TEAD1, RAF1, CENPE, GRHL3, ANKRD27, CYBA, P2RX7, HNF4A, GRTP1, LCP2 | 4.38.E-01 |
| *GO:0048598~embryonic morphogenesis* | 20 | 1.96.E-02 | TCF7, FGF9, MYO7A, DICER1, ITGB5, TEAD1, FGF10, GRHL3, SDC4, SCRIB, RIC8A, MAP3K7, PFN1, NCOA3, HDAC1, CRB2, MYO15, MIR96, SETD2, PITX2 | 4.42.E-01 |
| *GO:0015849~organic acid transport* | 6 | 1.99.E-02 | ABCB11, FABP3, SLC38A10, PEX3, SLC7A11, ACSL5 | 4.44.E-01 |
| *GO:0022610~biological adhesion* | 40 | 2.02.E-02 | ADAMTS18, PARD3, YWHAZ, CLSTN1, DICER1, PTPN23, ITGB5, PIP5K1C, SDC4, CDSN, SCRIB, CDC42, CDKN2A, GLYCAM1, SORBS2, SMOC1, SATB1, TCF7, IL2RA, ROCK1, MPDZ, PCDH12, ACKR3, PTPRU, SLC7A11, RIC8A, PCDH18, LAMA1, ARVCF, DSG1B, MYO10, P2RX7, JMJD6, AIRE, DSC2, EFNA5, JAM2, DBN1, CDH10, GBP2 | 4.47.E-01 |
| *GO:0048857~neural nucleus development* | 5 | 2.07.E-02 | CDC42, FGF9, FGF2, SCRIB, PITX2 | 4.53.E-01 |
| *GO:0001932~regulation of protein phosphorylation* | 34 | 2.16.E-02 | PARD3, FGF7, FGF9, DICER1, FGF10, SDC4, DTNBP1, TIMP3, MAP3K7, CDC42, CDKN2A, HSF1, RSPO1, MAP3K1, FGF2, THPO, DIXDC1, PKIG, PRKAB1, TEAD1, RAF1, CENPE, ACKR3, P2RX7, ATF3, PROM2, AKTIP, HNF4A, HDAC1, FGFR1OP, ASH1L, EFNA5, LRRK1, LCP2 | 4.64.E-01 |
| *GO:0051656~establishment of organelle localization* | 13 | 2.19.E-02 | YWHAZ, MYO7A, SYNJ1, FGF10, CENPE, DTNBP1, SCRIB, CDC42, P2RX7, MCPH1, RAB5A, RAB11A, GPSM2 | 4.67.E-01 |
| *GO:0044087~regulation of cellular component biogenesis* | 23 | 2.22.E-02 | DNM1L, ROCK1, BAIAP2L2, HIP1R, CLSTN1, RAF1, ABI2, SDC4, MAP3K7, ANKRD27, CDC42, PFN1, P2RX7, MYO10, HSF1, 5330417C22RIK, HNF4A, MAP3K1, RAB5A, EFNA5, NEDD4L, PARP1, DBN1 | 4.69.E-01 |
| *GO:0097191~extrinsic apoptotic signaling pathway* | 10 | 2.22.E-02 | SIVA1, P2RX7, ZC3HC1, ATF3, LMNA, RAF1, FGF10, FOXO3, ITM2C, TIMP3 | 4.68.E-01 |
| *GO:0046903~secretion* | 29 | 2.30.E-02 | YWHAZ, FGF7, HIP1R, SYNJ1, NCS1, FGF10, PIP5K1C, SDC4, DTNBP1, SCRIB, PFN1, C1QTNF5, RAB11A, TMEM79, DNM1L, PLA2G10, ABCB11, RHBDF1, RAF1, CYBA, P2RX7, HNF4A, HDAC1, LYST, RAB5A, EFNA5, PARP1, PLA2G2E, LCP2 | 4.77.E-01 |
| *GO:1902589~single-organism organelle organization* | 42 | 2.34.E-02 | ARL6IP1, BBS5, PARD3, FGF7, PRC1, SHROOM4, MAU2, HIP1R, DICER1, HBS1L, ITGB5, ABI2, FGF10, PIP5K1C, MIGA1, PEX3, SDC4, DTNBP1, CDC42, PFN1, CISD2, FRMD6, SORBS2, MAP3K1, RAB11A, GPSM2, HSF2BP, CAP2, DNM1L, ROCK1, BAIAP2L2, LMNA, TBCE, RAF1, CENPE, KNSTRN, ANKRD27, LIMCH1, FGFR1OP, MCPH1, IFT81, DBN1 | 4.81.E-01 |
| *GO:0050790~regulation of catalytic activity* | 41 | 2.34.E-02 | ARL6IP1, FDX1, HIP1R, FGF10, NFKB1, SDC4, DTNBP1, TIMP3, SCRIB, PDCD2, MAP3K7, PFN1, CDC42, CDKN2A, HSF1, CRB2, PGAM5, MAP3K1, SETMAR, FAM162A, FGF2, ELMOD1, CAP2, ROCK1, PKIG, PRKAB1, RAF1, TEAD1, CENPE, GRHL3, ANKRD27, CYBA, P2RX7, PROM2, HNF4A, FGFR1OP, GRTP1, MCPH1, EFNA5, DBN1, LCP2 | 4.79.E-01 |
| *GO:0044093~positive regulation of molecular function* | 33 | 2.35.E-02 | HIP1R, FDX1, FGF10, SDC4, SCRIB, PDCD2, MAP3K7, PFN1, CDC42, CDKN2A, HSF1, PGAM5, MAP3K1, SETMAR, FAM162A, NEDD4L, FGF2, PITX2, ELMOD1, RAF1, TEAD1, CENPE, GRHL3, ANKRD27, CYBA, P2RX7, CTH, AKTIP, HNF4A, NCOA3, GRTP1, PARP1, LCP2 | 4.78.E-01 |
| *GO:0001934~positive regulation of protein phosphorylation* | 25 | 2.44.E-02 | DIXDC1, FGF7, FGF9, TEAD1, FGF10, RAF1, ACKR3, CENPE, SDC4, DTNBP1, MAP3K7, CDC42, P2RX7, PROM2, RSPO1, HSF1, HNF4A, HDAC1, AKTIP, MAP3K1, EFNA5, LRRK1, FGF2, THPO, LCP2 | 4.89.E-01 |
| *GO:0022603~regulation of anatomical structure morphogenesis* | 28 | 2.44.E-02 | FGF7, DICER1, FGF10, DTNBP1, SCRIB, SDC2, CDC42, WARS, CDKN2A, CRB2, SEMA3F, RAB11A, NEDD4L, TCF4, FGF2, DIXDC1, DNM1L, ROCK1, CSNK1G2, ADIPOR1, GRHL3, SPARC, ANKRD27, MYO10, HNF4A, EFNA5, ARAP3, DBN1 | 4.87.E-01 |
| *GO:0031401~positive regulation of protein modification process* | 29 | 2.46.E-02 | FGF7, FGF9, FGF10, SDC4, DTNBP1, MAP3K7, CDC42, AHRR, CDKN2A, HSF1, RSPO1, MAP3K1, FGF2, THPO, DIXDC1, NOS1AP, TEAD1, RAF1, NDFIP2, ACKR3, CENPE, P2RX7, PROM2, HNF4A, HDAC1, AKTIP, EFNA5, LRRK1, LCP2 | 4.88.E-01 |
| *GO:0001525~angiogenesis* | 15 | 2.47.E-02 | ROCK1, FGF9, DICER1, FGF10, ACKR3, SPARC, ELK3, MAP3K7, CDC42, WARS, JMJD6, TCF4, SETD2, FGF2, PITX2 | 4.87.E-01 |
| *GO:0006644~phospholipid metabolic process* | 12 | 2.49.E-02 | CDC42, FGF7, PLA2G10, LYST, SYNJ1, FABP3, GGPS1, PIP5K1C, MVK, PLA2G2E, FGF2, ACSL5 | 4.87.E-01 |
| *GO:0016571~histone methylation* | 7 | 2.50.E-02 | DOT1L, SATB1, ASH1L, NCOA6, SETMAR, SETD6, SETD2 | 4.87.E-01 |
| *GO:0009913~epidermal cell differentiation* | 9 | 2.53.E-02 | CDC42, HDAC1, NCOA3, ROCK1, MYO7A, MIR96, DNASE1L2, SCRIB, TMEM79 | 4.89.E-01 |
| *GO:0034968~histone lysine methylation* | 6 | 2.56.E-02 | DOT1L, ASH1L, NCOA6, SETMAR, SETD6, SETD2 | 4.90.E-01 |
| *GO:0016070~RNA metabolic process* | 92 | 2.59.E-02 | SPIN1, FGF7, FGF9, DICER1, YLPM1, TRMT10A, FGF10, CBX2, NFKB1, FOXO3, USP50, MAP3K7, WARS, EPC2, CDKN2A, BARX2, HSF1, RNF38, DBR1, LSM1, FTSJ1, NFIL3, PITX3, FGF2, PITX2, RBFOX1, SATB1, KHDRBS3, NBAS, NOL8, PKIG, GRHL3, GTF2B, ZBTB24, ELL2, ARHGEF11, SARNP, CTH, PRDM4, NCOA3, HNF4A, ASCC2, MTF1, NAB2, ASH1L, NCOA6, RAB5A, VOPP1, JMJD1C, TRRAP, ELK3, DUSP11, CDKAL1, BMS1, TCF7L1, PFN1, AHRR, CRB2, TYW1, HSF2BP, RNF10, TCF4, NSUN2, MYSM1, BAHD1, TCF7, PLA2G10, GMNN, PRIMPOL, ASXL1, RFX7, LMNA, RAF1, TEAD1, UCP1, INTS10, PLAC8, DOT1L, ATF3, HDAC1, DCP2, ZFP182, JMJD6, GSPT2, AIRE, KDM8, ZBTB4, SETD6, AHCYL1, DHX40, SETD2, PARP1 | 4.93.E-01 |
| *GO:0070848~response to growth factor* | 19 | 2.62.E-02 | FGF7, FGF9, ITGB5, FGF10, RAF1, NFKB1, MAPKAPK2, SPARC, FOXO3, MAP3K7, PFN1, CRB2, MAP3K1, RAB35, SMURF2, TCF4, PARP1, FGF2, DBN1 | 4.94.E-01 |
| *GO:0006730~one-carbon metabolic process* | 4 | 2.65.E-02 | MTHFSL, MTHFD2, ALDH1L1, AHCYL1 | 4.97.E-01 |
| *GO:0048566~embryonic digestive tract development* | 4 | 2.65.E-02 | TCF7, FGF9, FGF10, PITX2 | 4.97.E-01 |
| *GO:0048562~embryonic organ morphogenesis* | 12 | 2.69.E-02 | TCF7, FGF9, CRB2, MYO7A, MYO15, FGF10, TEAD1, GRHL3, MIR96, SETD2, SCRIB, PITX2 | 4.99.E-01 |
| *GO:0031643~positive regulation of myelination* | 3 | 2.75.E-02 | PARD3, DICER1, RNF10 | 5.06.E-01 |
| *GO:0061029~eyelid development in camera-type eye* | 3 | 2.75.E-02 | HDAC1, MAP3K1, GRHL3 | 5.06.E-01 |
| *GO:0000737~DNA catabolic process, endonucleolytic* | 3 | 2.75.E-02 | HSF1, SETMAR, DICER1 | 5.06.E-01 |
| *GO:0048568~embryonic organ development* | 16 | 2.76.E-02 | TCF7, FGF9, MYO7A, TEAD1, FGF10, PCDH12, GRHL3, SCRIB, HSF1, NCOA3, CRB2, NCOA6, MYO15, MIR96, SETD2, PITX2 | 5.05.E-01 |
| *GO:0019220~regulation of phosphate metabolic process* | 40 | 2.78.E-02 | PARD3, FGF7, FGF9, DICER1, FGF10, SDC4, DTNBP1, TIMP3, MAP3K7, CDC42, CDKN2A, HSF1, RSPO1, MAP3K1, FGF2, THPO, DIXDC1, CAP2, DNM1L, ROCK1, PKIG, PRKAB1, RAF1, TEAD1, CENPE, ACKR3, P2RX7, ATF3, PROM2, AKTIP, HDAC1, HNF4A, FGFR1OP, MCPH1, ASH1L, FABP3, EFNA5, PARP1, LRRK1, LCP2 | 5.05.E-01 |
| *GO:0051174~regulation of phosphorus metabolic process* | 40 | 2.81.E-02 | PARD3, FGF7, FGF9, DICER1, FGF10, SDC4, DTNBP1, TIMP3, MAP3K7, CDC42, CDKN2A, HSF1, RSPO1, MAP3K1, FGF2, THPO, DIXDC1, CAP2, DNM1L, ROCK1, PKIG, PRKAB1, RAF1, TEAD1, CENPE, ACKR3, P2RX7, ATF3, PROM2, AKTIP, HDAC1, HNF4A, FGFR1OP, MCPH1, ASH1L, FABP3, EFNA5, PARP1, LRRK1, LCP2 | 5.07.E-01 |
| *GO:0007155~cell adhesion* | 39 | 2.82.E-02 | ADAMTS18, PARD3, YWHAZ, CLSTN1, DICER1, PTPN23, ITGB5, PIP5K1C, SDC4, CDSN, SCRIB, CDC42, CDKN2A, GLYCAM1, SORBS2, SMOC1, SATB1, TCF7, IL2RA, ROCK1, MPDZ, PCDH12, ACKR3, PTPRU, SLC7A11, RIC8A, PCDH18, LAMA1, ARVCF, DSG1B, MYO10, P2RX7, JMJD6, AIRE, DSC2, EFNA5, JAM2, DBN1, CDH10 | 5.06.E-01 |
| *GO:1905039~carboxylic acid transmembrane transport* | 4 | 2.84.E-02 | SLC38A10, PEX3, SLC7A11, ACSL5 | 5.07.E-01 |
| *GO:1903825~organic acid transmembrane transport* | 4 | 2.84.E-02 | SLC38A10, PEX3, SLC7A11, ACSL5 | 5.07.E-01 |
| *GO:0042471~ear morphogenesis* | 7 | 2.85.E-02 | FGF9, MYO7A, MYO15, FGF10, GRHL3, MIR96, SCRIB | 5.06.E-01 |
| *GO:0001944~vasculature development* | 20 | 2.93.E-02 | ROCK1, FGF9, DICER1, FGF10, ACKR3, DCTN5, SPARC, ELK3, RIC8A, MAP3K7, LAMA1, WARS, CDC42, CTH, JMJD6, NCOA6, TCF4, SETD2, FGF2, PITX2 | 5.14.E-01 |
| *GO:0060425~lung morphogenesis* | 5 | 2.97.E-02 | LAMA1, CDC42, FGF7, FGF10, PITX2 | 5.16.E-01 |
| *GO:0032268~regulation of cellular protein metabolic process* | 53 | 2.97.E-02 | FGF7, FGF9, HIP1R, DICER1, FGF10, NFKB1, FOXO3, SDC4, PDCD2, MAP3K7, CDC42, CDKN2A, HSF1, RSPO1, FGF2, NOS1AP, ROCK1, PKIG, PRKAB1, ACKR3, SARNP, HNF4A, FGFR1OP, ASH1L, EFNA5, MVK, LRRK1, LCP2, CLN6, ARL6IP1, PARD3, TIMP3, DTNBP1, AHRR, CRB2, MAP3K1, FAM162A, THPO, DIXDC1, RNF144B, PLA2G10, RHBDF1, NDFIP2, TEAD1, RAF1, CENPE, P2RX7, ATF3, PROM2, NXN, HDAC1, AKTIP, GRB7 | 5.15.E-01 |
| *GO:0035239~tube morphogenesis* | 14 | 3.00.E-02 | TCF7, DICER1, TEAD1, FGF10, GRHL3, SDC4, SCRIB, MAP3K7, LAMA1, PFN1, NCOA3, SETD2, FGF2, PITX2 | 5.17.E-01 |
| *GO:0016477~cell migration* | 31 | 3.02.E-02 | PARD3, FGF7, PTPN23, ABI2, FGF10, SDC4, SCRIB, SDC2, CDC42, PFN1, SORBS2, SEMA3F, RAB11A, FGF2, PITX2, DIXDC1, SPNS2, ROCK1, RHBDF1, ADIPOR1, SPARC, PTPRU, ELMO3, RIC8A, FGFR1OP, LYST, RAB5A, SMURF2, ARAP3, SETD2, GRB7 | 5.17.E-01 |
| *GO:0007264~small GTPase mediated signal transduction* | 16 | 3.03.E-02 | GDI2, ROCK1, FGF10, ABI2, ARHGEF11, CDC42, PLEKHG3, ARHGAP33, RAB19, ARF3, RAB35, RAB5A, RAB11A, AGAP1, ARAP3, LRRK1 | 5.16.E-01 |
| *GO:0060562~epithelial tube morphogenesis* | 13 | 3.03.E-02 | DICER1, FGF10, TEAD1, GRHL3, SDC4, SCRIB, MAP3K7, LAMA1, PFN1, NCOA3, SETD2, FGF2, PITX2 | 5.14.E-01 |
| *GO:0007032~endosome organization* | 4 | 3.04.E-02 | AKTIP, SYNJ1, RAB5A, RAB11A | 5.13.E-01 |
| *GO:0007435~salivary gland morphogenesis* | 4 | 3.04.E-02 | LAMA1, CDC42, FGF7, FGF10 | 5.13.E-01 |
| *GO:0035088~establishment or maintenance of apical/basal cell polarity* | 4 | 3.04.E-02 | LAMA1, CDC42, CRB2, SCRIB | 5.13.E-01 |
| *GO:0061245~establishment or maintenance of bipolar cell polarity* | 4 | 3.04.E-02 | LAMA1, CDC42, CRB2, SCRIB | 5.13.E-01 |
| *GO:0045184~establishment of protein localization* | 45 | 3.05.E-02 | ARL6IP1, YWHAZ, PARD3, BBS5, FGF9, PLEKHM1, MYO7A, PTPN23, VPS37D, PEX3, CDC42, C1QTNF5, RAB11A, TNPO2, AGAP1, ELMOD1, GDI2, DNM1L, DUOXA2, ROCK1, RHBDF1, PKIG, LMNA, TOMM40, NDFIP2, RAF1, CENPE, ANKRD27, P2RX7, ARHGAP33, AKTIP, HDAC1, HNF4A, ARF3, RAB35, LYST, GRTP1, RAB5A, EFNA5, NUTF2, YIPF5, IFT81, AHCYL1, PARP1, LCP2 | 5.13.E-01 |
| *GO:1902580~single-organism cellular localization* | 26 | 3.06.E-02 | ARL6IP1, YWHAZ, PARD3, FGF9, MYO7A, SYNJ1, FGF10, PEX3, DTNBP1, SCRIB, CDC42, ZDHHC23, GPSM2, RAB11A, TNPO2, FYB, ROCK1, PKIG, LMNA, TOMM40, P2RX7, HNF4A, MCPH1, RAB5A, NUTF2, PARP1 | 5.12.E-01 |
| *GO:0008610~lipid biosynthetic process* | 17 | 3.06.E-02 | FGF7, FDX1, HSD17B1, PRKAB1, PIP5K1C, LSS, NFKB1, P2RX7, ST6GALNAC3, HNF4A, CYP7A1, FABP3, GGPS1, MVK, FGF2, AKR1D1, ACSL5 | 5.11.E-01 |
| *GO:0018027~peptidyl-lysine dimethylation* | 3 | 3.11.E-02 | ASH1L, SETMAR, SETD2 | 5.14.E-01 |
| *GO:0010256~endomembrane system organization* | 16 | 3.12.E-02 | ARL6IP1, FYB, YWHAZ, ROCK1, BAIAP2L2, SYNJ1, LMNA, DTNBP1, ZDHHC23, CDC42, P2RX7, AKTIP, LYST, RAB5A, RAB11A, NUTF2 | 5.14.E-01 |
| *GO:0051240~positive regulation of multicellular organismal process* | 39 | 3.13.E-02 | PARD3, FGF7, FGF9, CLSTN1, SYNJ1, DICER1, FGF10, MAPKAPK2, FOXO3, PDCD2, MAP3K7, CDC42, PFN1, ASPA, CDKN2A, HSF1, CRB2, RAB11A, RNF10, NEDD4L, TCF4, PITX3, FGF2, THPO, DIXDC1, IL2RA, LMNA, TEAD1, SPARC, ANKRD27, CYBA, P2RX7, CTH, HDAC1, NCOA3, EFNA5, SMURF2, PARP1, DBN1 | 5.12.E-01 |
| *GO:0007169~transmembrane receptor protein tyrosine kinase signaling pathway* | 16 | 3.17.E-02 | FGF7, BAIAP2L2, FGF9, HIP1R, RHBDF1, ADIPOR1, FGF10, RAF1, RPE65, MAPKAPK2, COL4A5, CDC42, EFNA5, EFNA4, FGF2, LCP2 | 5.16.E-01 |
| *GO:0007167~enzyme linked receptor protein signaling pathway* | 23 | 3.21.E-02 | FGF7, BAIAP2L2, FGF9, HIP1R, RHBDF1, ADIPOR1, ITGB5, FGF10, RAF1, RPE65, MAPKAPK2, COL4A5, MAP3K7, CDC42, HNF4A, CRB2, MAP3K1, EFNA5, SMURF2, EFNA4, PARP1, FGF2, LCP2 | 5.18.E-01 |
| *GO:0045682~regulation of epidermis development* | 5 | 3.26.E-02 | NCOA3, ROCK1, NAB2, MYSM1, TMEM79 | 5.22.E-01 |
| *GO:1901615~organic hydroxy compound metabolic process* | 13 | 3.28.E-02 | FDX1, HSD17B1, CYP7A1, SYNJ1, SLC24A5, ALDH2, NFKB1, LSS, MVK, RPE65, AKR1D1, FGF2, CLN6 | 5.22.E-01 |
| *GO:0007017~microtubule-based process* | 19 | 3.36.E-02 | DIXDC1, PARD3, PRC1, KIF5A, DICER1, TBCE, LMNA, FGF10, CENPE, KNSTRN, DTNBP1, CDC42, FGFR1OP, LYST, MCPH1, RAB11A, GPSM2, IFT81, EFNA5 | 5.28.E-01 |
| *GO:0042325~regulation of phosphorylation* | 35 | 3.40.E-02 | PARD3, FGF7, FGF9, DICER1, FGF10, SDC4, DTNBP1, TIMP3, MAP3K7, CDC42, CDKN2A, HSF1, RSPO1, MAP3K1, FGF2, THPO, DIXDC1, PKIG, PRKAB1, RAF1, TEAD1, CENPE, ACKR3, P2RX7, ATF3, PROM2, AKTIP, HNF4A, HDAC1, FGFR1OP, MCPH1, ASH1L, EFNA5, LRRK1, LCP2 | 5.31.E-01 |
| *GO:0051017~actin filament bundle assembly* | 7 | 3.44.E-02 | PFN1, CDC42, BAIAP2L2, ROCK1, ITGB5, SDC4, DBN1 | 5.34.E-01 |
| *GO:0016310~phosphorylation* | 48 | 3.45.E-02 | PARD3, FGF7, FGF9, DICER1, ABI2, FGF10, PIP5K1C, MAPKAPK2, SDC4, DTNBP1, TIMP3, GALK2, MAP3K7, CDC42, CDKN2A, HSF1, RSPO1, AAK1, MAP3K1, FGF2, NDUFS1, THPO, DIXDC1, NADK2, ROCK1, CSNK1G2, PKIG, PRKAB1, RAF1, TEAD1, CENPE, ACKR3, LAMA1, P2RX7, ATF3, PROM2, AKTIP, HDAC1, HNF4A, FGFR1OP, ASH1L, MCPH1, BMP2K, EFNA5, MVK, LRRK1, LCP2, UQCRB | 5.33.E-01 |
| *GO:0018022~peptidyl-lysine methylation* | 6 | 3.46.E-02 | DOT1L, ASH1L, NCOA6, SETMAR, SETD6, SETD2 | 5.32.E-01 |
| *GO:0046164~alcohol catabolic process* | 4 | 3.46.E-02 | CYP7A1, SYNJ1, ALDH2, AKR1D1 | 5.30.E-01 |
| *GO:0061033~secretion by lung epithelial cell involved in lung growth* | 2 | 3.46.E-02 | FGF7, FGF10 | 5.29.E-01 |
| *GO:0060661~submandibular salivary gland formation* | 2 | 3.46.E-02 | CDC42, FGF10 | 5.29.E-01 |
| *GO:0071338~positive regulation of hair follicle cell proliferation* | 2 | 3.46.E-02 | CDC42, FGF10 | 5.29.E-01 |
| *GO:0051345~positive regulation of hydrolase activity* | 15 | 3.47.E-02 | HIP1R, FGF10, GRHL3, SCRIB, PDCD2, ANKRD27, PFN1, CDKN2A, HSF1, PGAM5, SETMAR, GRTP1, FAM162A, FGF2, ELMOD1 | 5.29.E-01 |
| *GO:0061180~mammary gland epithelium development* | 5 | 3.57.E-02 | CDKN2A, NCOA3, FGF10, FGF2, SCRIB | 5.36.E-01 |
| *GO:0030203~glycosaminoglycan metabolic process* | 6 | 3.58.E-02 | NFKB1, CHST5, SDC4, FGF2, SDC2, CLN6 | 5.36.E-01 |
| *GO:0006629~lipid metabolic process* | 31 | 3.58.E-02 | FGF7, HSD17B1, FDX1, ABCD1, SYNJ1, PIP5K1C, LSS, NFKB1, RPE65, CDC42, ST6GALNAC3, CYP7A1, ETFDH, GGPS1, 4932438A13RIK, FGF2, ACSL5, SPNS2, PLA2G10, PRKAB1, ADIPOR1, CYP4F14, P2RX7, HNF4A, LYST, FABP3, MVK, PLA2G2E, AKR1D1, CLN6, FMC1 | 5.35.E-01 |
| *GO:0015711~organic anion transport* | 11 | 3.59.E-02 | ARL6IP1, P2RX7, PLA2G10, ABCB11, ABCD1, FABP3, SLC38A10, PLA2G2E, DTNBP1, SLC7A11, ACSL5 | 5.34.E-01 |
| *GO:0010632~regulation of epithelial cell migration* | 8 | 3.61.E-02 | PFN1, FGF7, PTPN23, FGF10, ADIPOR1, RAB11A, SPARC, FGF2 | 5.34.E-01 |
| *GO:0060041~retina development in camera-type eye* | 8 | 3.61.E-02 | RAB11FIP4, LAMA1, C1QTNF5, JMJD6, CRB2, GRM6, RPE65, MIR96 | 5.34.E-01 |
| *GO:0051246~regulation of protein metabolic process* | 56 | 3.65.E-02 | FGF7, FGF9, HIP1R, DICER1, FGF10, NFKB1, FOXO3, SDC4, PDCD2, MAP3K7, CDC42, CDKN2A, HSF1, RSPO1, FGF2, NOS1AP, ROCK1, PKIG, PRKAB1, ACKR3, SARNP, HNF4A, FGFR1OP, ASH1L, EFNA5, MVK, LRRK1, LCP2, CLN6, ARL6IP1, PARD3, MAPKAPK2, TIMP3, DTNBP1, ITM2C, AHRR, CRB2, MAP3K1, FAM162A, NEDD4L, THPO, DIXDC1, RNF144B, PLA2G10, RHBDF1, NDFIP2, TEAD1, RAF1, CENPE, P2RX7, ATF3, PROM2, AKTIP, NXN, HDAC1, GRB7 | 5.36.E-01 |
| *GO:0033059~cellular pigmentation* | 4 | 3.68.E-02 | ANKRD27, MYO7A, LYST, RAB11A | 5.37.E-01 |
| *GO:0030334~regulation of cell migration* | 21 | 3.70.E-02 | FGF7, PTPN23, LMNA, ADIPOR1, FGF10, SPARC, PTPRU, SDC4, LAMA1, PFN1, FGFR1OP, MAP3K1, SEMA3F, RAB5A, RAB11A, SMURF2, ARAP3, FGF2, GRB7, MYSM1, PITX2 | 5.38.E-01 |
| *GO:0010634~positive regulation of epithelial cell migration* | 6 | 3.71.E-02 | PFN1, FGF7, FGF10, RAB11A, SPARC, FGF2 | 5.37.E-01 |
| *GO:0031399~regulation of protein modification process* | 39 | 3.73.E-02 | PARD3, FGF7, FGF9, DICER1, FGF10, SDC4, DTNBP1, TIMP3, MAP3K7, CDC42, AHRR, CDKN2A, HSF1, RSPO1, MAP3K1, FGF2, THPO, DIXDC1, NOS1AP, ROCK1, PKIG, PRKAB1, NDFIP2, RAF1, TEAD1, CENPE, ACKR3, P2RX7, ATF3, PROM2, AKTIP, HDAC1, NXN, HNF4A, FGFR1OP, ASH1L, EFNA5, LRRK1, LCP2 | 5.37.E-01 |
| *GO:0008654~phospholipid biosynthetic process* | 7 | 3.76.E-02 | FGF7, FABP3, GGPS1, PIP5K1C, MVK, FGF2, ACSL5 | 5.38.E-01 |
| *GO:0061572~actin filament bundle organization* | 7 | 3.76.E-02 | PFN1, CDC42, BAIAP2L2, ROCK1, ITGB5, SDC4, DBN1 | 5.38.E-01 |
| *GO:0042327~positive regulation of phosphorylation* | 25 | 3.85.E-02 | DIXDC1, FGF7, FGF9, TEAD1, FGF10, RAF1, ACKR3, CENPE, SDC4, DTNBP1, MAP3K7, CDC42, P2RX7, PROM2, RSPO1, HSF1, HNF4A, HDAC1, AKTIP, MAP3K1, EFNA5, LRRK1, FGF2, THPO, LCP2 | 5.45.E-01 |
| *GO:0022612~gland morphogenesis* | 7 | 3.87.E-02 | LAMA1, CDC42, FGF7, CDKN2A, NCOA3, FGF10, SCRIB | 5.46.E-01 |
| *GO:0002093~auditory receptor cell morphogenesis* | 3 | 3.87.E-02 | MYO7A, MIR96, SCRIB | 5.44.E-01 |
| *GO:0060113~inner ear receptor cell differentiation* | 5 | 3.89.E-02 | MYO7A, DICER1, MIR96, SDC4, SCRIB | 5.44.E-01 |
| *GO:0060606~tube closure* | 6 | 3.97.E-02 | PFN1, FGF10, GRHL3, SETD2, SDC4, SCRIB | 5.50.E-01 |
| *GO:0006694~steroid biosynthetic process* | 7 | 3.98.E-02 | FDX1, HSD17B1, CYP7A1, NFKB1, LSS, MVK, AKR1D1 | 5.50.E-01 |
| *GO:0006869~lipid transport* | 11 | 4.00.E-02 | SPNS2, P2RX7, STRA6L, PLA2G10, ABCB11, ABCD1, APOC4, FABP3, ATP11A, PLA2G2E, ACSL5 | 5.50.E-01 |
| *GO:0048699~generation of neurons* | 37 | 4.01.E-02 | MFSD8, MYO7A, SYNJ1, DICER1, NCS1, ABI2, PIP5K1C, RPE65, FOXO3, SDC4, DTNBP1, ITM2C, SCRIB, SDC2, CDC42, ASPA, C1QTNF5, SEMA3F, RAB11A, RNF10, NEDD4L, TCF4, PITX3, LSM1, PITX2, DIXDC1, PLA2G10, KIF5A, TBCE, ANKRD27, LAMA1, HDAC1, RAB35, EFNA5, EFNA4, MIR96, DBN1 | 5.49.E-01 |
| *GO:0030182~neuron differentiation* | 34 | 4.07.E-02 | MFSD8, MYO7A, DICER1, NCS1, ABI2, PIP5K1C, RPE65, FOXO3, SDC4, DTNBP1, ITM2C, SCRIB, SDC2, CDC42, C1QTNF5, SEMA3F, RAB11A, NEDD4L, TCF4, PITX3, LSM1, PITX2, DIXDC1, PLA2G10, KIF5A, TBCE, ANKRD27, LAMA1, HDAC1, RAB35, EFNA5, EFNA4, MIR96, DBN1 | 5.53.E-01 |
| *GO:0001667~ameboidal-type cell migration* | 12 | 4.07.E-02 | PFN1, FGF7, SEMA3F, PTPN23, FGF10, ADIPOR1, RAB11A, SMURF2, SPARC, FGF2, RIC8A, PITX2 | 5.51.E-01 |
| *GO:0007431~salivary gland development* | 4 | 4.14.E-02 | LAMA1, CDC42, FGF7, FGF10 | 5.55.E-01 |
| *GO:0048584~positive regulation of response to stimulus* | 43 | 4.16.E-02 | SPIN1, FGF9, HIP1R, FGF10, NFKB1, MAPKAPK2, FOXO3, ITM2C, TIMP3, CANT1, MAP3K7, CDC42, CDKN2A, HSF1, RSPO1, CRB2, AAK1, MAP3K1, SETMAR, FAM162A, FGF2, THPO, FYB, DIXDC1, BRCC3, DNM1L, ASXL1, ADIPOR1, NDFIP2, RAF1, ACKR3, CYBA, P2RX7, CTH, ATF3, HDAC1, HNF4A, NCOA3, MIB2, PARP1, DBN1, LRRK1, LCP2 | 5.56.E-01 |
| *GO:0051270~regulation of cellular component movement* | 23 | 4.19.E-02 | FGF7, PTPN23, LMNA, ADIPOR1, FGF10, SPARC, PTPRU, SDC4, LAMA1, PFN1, FGFR1OP, MAP3K1, SEMA3F, RAB5A, RAB11A, DSC2, SMURF2, ARAP3, DBN1, FGF2, GRB7, MYSM1, PITX2 | 5.57.E-01 |
| *GO:0006909~phagocytosis* | 8 | 4.22.E-02 | CYBA, CDC42, P2RX7, JMJD6, MYO7A, RAB5A, PIP5K1C, ELMO3 | 5.58.E-01 |
| *GO:0006887~exocytosis* | 11 | 4.25.E-02 | PFN1, YWHAZ, P2RX7, SYNJ1, NCS1, RAB5A, PIP5K1C, RAB11A, SDC4, SCRIB, TMEM79 | 5.59.E-01 |
| *GO:1904385~cellular response to angiotensin* | 3 | 4.28.E-02 | CYBA, HSF1, AHCYL1 | 5.60.E-01 |
| *GO:2000193~positive regulation of fatty acid transport* | 3 | 4.28.E-02 | P2RX7, PLA2G10, ACSL5 | 5.60.E-01 |
| *GO:0000075~cell cycle checkpoint* | 8 | 4.32.E-02 | DOT1L, BRCC3, HSF1, SETMAR, CENPE, MAPKAPK2, TRRAP, NSUN2 | 5.62.E-01 |
| *GO:0034660~ncRNA metabolic process* | 14 | 4.34.E-02 | SPIN1, NOL8, DICER1, TRMT10A, NFKB1, INTS10, CDKAL1, BMS1, ELL2, WARS, CDKN2A, TYW1, FTSJ1, NSUN2 | 5.62.E-01 |
| *GO:0010876~lipid localization* | 12 | 4.35.E-02 | SPNS2, P2RX7, STRA6L, PLA2G10, ABCB11, ABCD1, APOC4, FABP3, ATP11A, PLA2G2E, 4932438A13RIK, ACSL5 | 5.61.E-01 |
| *GO:0006732~coenzyme metabolic process* | 11 | 4.47.E-02 | NADK2, MTHFSL, MTHFD2, P2RX7, ALDH1L1, HNF4A, FDX1, MVK, AHCYL1, PDHX, COQ6 | 5.70.E-01 |
| *GO:0032956~regulation of actin cytoskeleton organization* | 11 | 4.47.E-02 | DIXDC1, PFN1, BAIAP2L2, ROCK1, HIP1R, MAP3K1, ABI2, EFNA5, GRHL3, SDC4, DBN1 | 5.70.E-01 |
| *GO:0030030~cell projection organization* | 34 | 4.49.E-02 | BBS5, MYO7A, DICER1, NCS1, PTPN23, ABI2, PIP5K1C, SDC4, ITM2C, DTNBP1, SCRIB, SDC2, CDC42, PFN1, SEMA3F, RAB11A, NEDD4L, DIXDC1, PLA2G10, ROCK1, KIF5A, TBCE, ANKRD27, LAMA1, P2RX7, MYO10, PROM2, HNF4A, RAB35, RAB5A, EFNA5, IFT81, EFNA4, DBN1 | 5.70.E-01 |
| *GO:0048667~cell morphogenesis involved in neuron differentiation* | 16 | 4.58.E-02 | DIXDC1, PLA2G10, KIF5A, MYO7A, TBCE, PIP5K1C, DTNBP1, SCRIB, ANKRD27, LAMA1, SEMA3F, RAB11A, EFNA5, EFNA4, MIR96, DBN1 | 5.76.E-01 |
| *GO:0034394~protein localization to cell surface* | 4 | 4.62.E-02 | FGF7, FGF10, PTPRU, NEDD4L | 5.78.E-01 |
| *GO:0007548~sex differentiation* | 11 | 4.66.E-02 | TCF7, FGF7, HNF4A, NCOA3, FGF9, RNF38, FGF10, CBX2, FOXO3, JMJD1C, PITX2 | 5.79.E-01 |
| *GO:0008203~cholesterol metabolic process* | 6 | 4.68.E-02 | FDX1, CYP7A1, LSS, MVK, AKR1D1, CLN6 | 5.79.E-01 |
| *GO:0016339~calcium-dependent cell-cell adhesion via plasma membrane cell adhesion molecules* | 3 | 4.70.E-02 | ARVCF, PCDH12, PCDH18 | 5.80.E-01 |
| *GO:0034109~homotypic cell-cell adhesion* | 5 | 4.77.E-02 | ADAMTS18, PIP5K1C, DSC2, PTPRU, SLC7A11 | 5.83.E-01 |
| *GO:0019637~organophosphate metabolic process* | 25 | 4.88.E-02 | NADK2, FGF7, DNM1L, CAP2, PLA2G10, FDX1, SYNJ1, PIP5K1C, FBP2, CANT1, CDC42, P2RX7, LYST, ATP5S, FABP3, GGPS1, ADSL, MVK, PLA2G2E, PARP1, FGF2, PDHX, NDUFS1, ACSL5, UQCRB | 5.91.E-01 |
| *GO:0060284~regulation of cell development* | 26 | 4.90.E-02 | SYNJ1, DICER1, NCS1, FOXO3, DTNBP1, ITM2C, SDC2, CDC42, S1PR3, ASPA, CRB2, SEMA3F, RAB11A, RNF10, NEDD4L, TCF4, LSM1, PITX3, FGF2, DIXDC1, ROCK1, ADIPOR1, ANKRD27, HDAC1, EFNA5, DBN1 | 5.90.E-01 |
| *GO:0042490~mechanoreceptor differentiation* | 5 | 4.96.E-02 | MYO7A, DICER1, MIR96, SDC4, SCRIB | 5.93.E-01 |
| *GO:0048608~reproductive structure development* | 15 | 4.96.E-02 | TCF7, FGF7, HSF1, NCOA3, FGF9, ASH1L, NCOA6, DICER1, RNF38, PCDH12, FGF10, FOXO3, JMJD1C, SETD2, PITX2 | 5.92.E-01 |
| *GO:0046907~intracellular transport* | 32 | 4.99.E-02 | ARL6IP1, YWHAZ, PARD3, BBS5, FGF9, MYO7A, PTPN23, PEX3, DTNBP1, CDC42, CDKN2A, TNPO2, ELMOD1, KIF5A, PKIG, LMNA, TOMM40, SARNP, ANKRD27, MYO10, HNF4A, AKTIP, RAB35, LYST, GRTP1, RAB5A, YIPF5, NUTF2, AHCYL1, PARP1, SETD2, DBN1 | 5.92.E-01 |

Supplementary Table 3. A list of GO terms of “GOTERM_BP_FAT” with modified Fisher exact P-values less than 0.05 and their associated novel genes.

| Term | Count | P-Value | Genes | Benjamini |
| --- | --- | --- | --- | --- |
| GO:0055114~oxidation-reduction process | 28 | 5.16E-05 | ALDH1L1, HSD17B1, FDX1, ABCD1, MTHFD2, TYW1, ETFDH, GPX7, AGL, ACSL5, GDI2, NDUFA8, CYP4F14, PCDH12, CYB561, CYB561A3, ALDH16A1, CYP4B1, COQ6, CYBA, NXN, JMJD6, KDM8, ADSL, JMJD1C, AOC1, AKR1D1, UQCRB | 1.41E-01 |
| GO:0097676~histone H3-K36 dimethylation | 3 | 4.76E-04 | ASH1L, SETMAR, SETD2 | 5.04E-01 |
| GO:0016192~vesicle-mediated transport | 28 | 1.83E-03 | YWHAZ, HIP1R, SYNJ1, PTPN23, PIP5K1C, MAPKAPK2, SDC4, SCRIB, PFN1, CHIC2, RSPO1, AAK1, NEDD4L, TMEM79, GDI2, KIF5A, CSNK1G2, ELMO3, ANKRD27, CYBA, PROM2, AKTIP, JMJD6, ARF3, RAB35, GRTP1, RAB5A, YIPF5 | 8.34E-01 |
| GO:0016569~covalent chromatin modification | 16 | 2.47E-03 | SPIN1, ASXL1, CBX2, TRRAP, DOT1L, EPC2, JMJD6, ASH1L, SETMAR, NCOA6, KDM8, SETD6, JMJD1C, SETD2, MYSM1, BAHD1 | 8.39E-01 |
| GO:0015909~long-chain fatty acid transport | 5 | 3.94E-03 | PLA2G10, ABCD1, FABP3, PLA2G2E, ACSL5 | 9.03E-01 |
| GO:0048259~regulation of receptor-mediated endocytosis | 6 | 4.74E-03 | PROM2, RSPO1, HIP1R, AAK1, SYNJ1, NEDD4L | 9.03E-01 |
| GO:0010452~histone H3-K36 methylation | 3 | 5.43E-03 | ASH1L, SETMAR, SETD2 | 8.99E-01 |
| GO:0015849~organic acid transport | 6 | 5.48E-03 | ABCB11, FABP3, SLC38A10, PEX3, SLC7A11, ACSL5 | 8.68E-01 |
| GO:0045022~early endosome to late endosome transport | 4 | 5.76E-03 | ANKRD27, AKTIP, RAB5A, PTPN23 | 8.49E-01 |
| GO:0016570~histone modification | 13 | 6.25E-03 | ASXL1, TRRAP, DOT1L, EPC2, JMJD6, SETMAR, NCOA6, ASH1L, KDM8, SETD6, JMJD1C, SETD2, MYSM1 | 8.43E-01 |
| GO:0034968~histone lysine methylation | 6 | 7.20E-03 | DOT1L, ASH1L, NCOA6, SETMAR, SETD6, SETD2 | 8.56E-01 |
| GO:0098927~vesicle-mediated transport between endosomal compartments | 4 | 7.60E-03 | ANKRD27, AKTIP, RAB5A, PTPN23 | 8.47E-01 |
| GO:0033036~macromolecule localization | 49 | 8.77E-03 | PLEKHM1, PTPN23, PIP5K1C, PEX3, VPS37D, FRMD6, AGAP1, DUOXA2, KIF5A, ABCB11, PKIG, PTPRU, ANKRD27, SARNP, HNF4A, RAB5A, PLA2G2E, DBN1, LCP2, ARL6IP1, YWHAZ, ABCD1, SCRIB, ZDHHC23, PFN1, APOC4, NEDD4L, 4932438A13RIK, TNPO2, ELMOD1, ACSL5, FYB, SPNS2, GDI2, PLA2G10, RHBDF1, NDFIP2, ATP11A, CENPE, STRA6L, AKTIP, RAB35, ARF3, GRTP1, FABP3, AHCYL1, YIPF5, NUTF2, SETD2 | 8.65E-01 |
| GO:0018022~peptidyl-lysine methylation | 6 | 1.00E-02 | DOT1L, ASH1L, NCOA6, SETMAR, SETD6, SETD2 | 8.81E-01 |
| GO:0098742~cell-cell adhesion via plasma-membrane adhesion molecules | 9 | 1.04E-02 | ARVCF, DSG1B, CLSTN1, PTPN23, PCDH12, DSC2, CDH10, RIC8A, PCDH18 | 8.72E-01 |
| GO:0006730~one-carbon metabolic process | 4 | 1.14E-02 | MTHFSL, MTHFD2, ALDH1L1, AHCYL1 | 8.78E-01 |
| GO:1905039~carboxylic acid transmembrane transport | 4 | 1.22E-02 | SLC38A10, PEX3, SLC7A11, ACSL5 | 8.82E-01 |
| GO:1903825~organic acid transmembrane transport | 4 | 1.22E-02 | SLC38A10, PEX3, SLC7A11, ACSL5 | 8.82E-01 |
| GO:0060627~regulation of vesicle-mediated transport | 13 | 1.31E-02 | HIP1R, SYNJ1, PTPN23, SDC4, ANKRD27, CYBA, PROM2, RSPO1, AAK1, GRTP1, RAB5A, YIPF5, NEDD4L | 8.85E-01 |
| GO:0010876~lipid localization | 11 | 1.36E-02 | SPNS2, STRA6L, PLA2G10, ABCB11, ABCD1, APOC4, FABP3, ATP11A, PLA2G2E, 4932438A13RIK, ACSL5 | 8.80E-01 |
| GO:0046942~carboxylic acid transport | 9 | 1.46E-02 | ARL6IP1, PLA2G10, ABCB11, ABCD1, FABP3, SLC38A10, PLA2G2E, SLC7A11, ACSL5 | 8.86E-01 |
| GO:0006869~lipid transport | 10 | 1.51E-02 | SPNS2, STRA6L, PLA2G10, ABCB11, ABCD1, APOC4, FABP3, ATP11A, PLA2G2E, ACSL5 | 8.83E-01 |
| GO:0006898~receptor-mediated endocytosis | 8 | 1.62E-02 | PROM2, RSPO1, HIP1R, AAK1, SYNJ1, RAB5A, NEDD4L, SCRIB | 8.88E-01 |
| GO:0006897~endocytosis | 14 | 1.67E-02 | CSNK1G2, HIP1R, SYNJ1, PIP5K1C, MAPKAPK2, ELMO3, SCRIB, CYBA, PROM2, RSPO1, JMJD6, AAK1, RAB5A, NEDD4L | 8.85E-01 |
| GO:0018027~peptidyl-lysine dimethylation | 3 | 1.71E-02 | ASH1L, SETMAR, SETD2 | 8.79E-01 |
| GO:0015718~monocarboxylic acid transport | 6 | 1.72E-02 | PLA2G10, ABCB11, ABCD1, FABP3, PLA2G2E, ACSL5 | 8.72E-01 |
| GO:0007156~homophilic cell adhesion via plasma membrane adhesion molecules | 7 | 1.82E-02 | DSG1B, CLSTN1, PTPN23, PCDH12, DSC2, CDH10, PCDH18 | 8.76E-01 |
| GO:0018205~peptidyl-lysine modification | 10 | 1.87E-02 | DOT1L, EPC2, CTH, JMJD6, ASH1L, NCOA6, SETMAR, SETD6, TRRAP, SETD2 | 8.73E-01 |
| GO:0015908~fatty acid transport | 5 | 2.12E-02 | PLA2G10, ABCD1, FABP3, PLA2G2E, ACSL5 | 8.95E-01 |
| GO:0030100~regulation of endocytosis | 8 | 2.28E-02 | CYBA, PROM2, RSPO1, HIP1R, AAK1, SYNJ1, RAB5A, NEDD4L | 9.05E-01 |
| GO:0043414~macromolecule methylation | 9 | 2.29E-02 | DOT1L, ASH1L, NCOA6, SETMAR, SETD6, TRMT10A, FTSJ1, SETD2, NSUN2 | 8.98E-01 |
| GO:0016571~histone methylation | 6 | 2.36E-02 | DOT1L, ASH1L, NCOA6, SETMAR, SETD6, SETD2 | 8.97E-01 |
| GO:0016339~calcium-dependent cell-cell adhesion via plasma membrane cell adhesion molecules | 3 | 2.62E-02 | ARVCF, PCDH12, PCDH18 | 9.13E-01 |
| GO:0006325~chromatin organization | 17 | 2.64E-02 | SPIN1, ASXL1, CBX2, TRRAP, PTMA, DOT1L, EPC2, JMJD6, ASH1L, SETMAR, NCOA6, KDM8, SETD6, JMJD1C, SETD2, MYSM1, BAHD1 | 9.09E-01 |
| GO:0046653~tetrahydrofolate metabolic process | 3 | 2.87E-02 | MTHFSL, MTHFD2, ALDH1L1 | 9.20E-01 |
| GO:0030488~tRNA methylation | 3 | 3.13E-02 | TRMT10A, FTSJ1, NSUN2 | 9.31E-01 |
| GO:0051276~chromosome organization | 22 | 3.25E-02 | SPIN1, MAU2, ASXL1, YLPM1, CENPE, CBX2, TRRAP, KNSTRN, PTMA, DOT1L, SLX4, EPC2, JMJD6, ASH1L, SETMAR, NCOA6, SETD6, KDM8, JMJD1C, SETD2, MYSM1, BAHD1 | 9.33E-01 |
| GO:0016578~histone deubiquitination | 3 | 3.40E-02 | ASXL1, TRRAP, MYSM1 | 9.36E-01 |
| GO:0070252~actin-mediated cell contraction | 4 | 3.41E-02 | FRMD6, DSC2, NEDD4L, DBN1 | 9.32E-01 |
| GO:0015711~organic anion transport | 9 | 3.55E-02 | ARL6IP1, PLA2G10, ABCB11, ABCD1, FABP3, SLC38A10, PLA2G2E, SLC7A11, ACSL5 | 9.35E-01 |
| GO:0006400~tRNA modification | 4 | 3.56E-02 | TRMT10A, CDKAL1, FTSJ1, NSUN2 | 9.31E-01 |
| GO:0051188~cofactor biosynthetic process | 6 | 3.75E-02 | NADK2, MTHFSL, MTHFD2, HMBS, PDHX, COQ6 | 9.36E-01 |
| GO:0060348~bone development | 7 | 3.90E-02 | SPNS2, HSD17B1, NAB2, ANKRD11, ASXL1, SLC38A10, LRRK1 | 9.39E-01 |
| GO:0009108~coenzyme biosynthetic process | 5 | 4.21E-02 | NADK2, MTHFSL, MTHFD2, PDHX, COQ6 | 9.48E-01 |
| GO:0006760~folic acid-containing compound metabolic process | 3 | 4.26E-02 | MTHFSL, MTHFD2, ALDH1L1 | 9.46E-01 |
| GO:0006732~coenzyme metabolic process | 9 | 4.30E-02 | NADK2, MTHFSL, MTHFD2, ALDH1L1, HNF4A, FDX1, AHCYL1, PDHX, COQ6 | 9.44E-01 |
| GO:0051186~cofactor metabolic process | 10 | 4.88E-02 | NADK2, MTHFSL, MTHFD2, ALDH1L1, HNF4A, FDX1, HMBS, AHCYL1, PDHX, COQ6 | 9.60E-01 |
| GO:0035522~monoubiquitinated histone H2A deubiquitination | 2 | 4.97E-02 | ASXL1, MYSM1 | 9.59E-01 |
| GO:0035521~monoubiquitinated histone deubiquitination | 2 | 4.97E-02 | ASXL1, MYSM1 | 9.59E-01 |
| GO:0008033~tRNA processing | 5 | 5.00E-02 | TYW1, TRMT10A, CDKAL1, FTSJ1, NSUN2 | 9.57E-01 |
| GO:1901361~organic cyclic compound catabolic process | 9 | 5.09E-02 | ALDH1L1, DCP2, SETMAR, GSPT2, AHCYL1, LSM1, DNASE1L2, AKR1D1, CANT1 | 9.57E-01 |
| GO:0034660~ncRNA metabolic process | 11 | 5.15E-02 | WARS, SPIN1, NOL8, TYW1, TRMT10A, CDKAL1, FTSJ1, INTS10, BMS1, NSUN2, ELL2 | 9.56E-01 |
| GO:0003333~amino acid transmembrane transport | 3 | 5.19E-02 | SLC38A10, PEX3, SLC7A11 | 9.54E-01 |
| GO:0016577~histone demethylation | 3 | 5.19E-02 | JMJD6, KDM8, JMJD1C | 9.54E-01 |
| GO:0008213~protein alkylation | 6 | 5.32E-02 | DOT1L, ASH1L, NCOA6, SETMAR, SETD6, SETD2 | 9.55E-01 |
| GO:0006479~protein methylation | 6 | 5.32E-02 | DOT1L, ASH1L, NCOA6, SETMAR, SETD6, SETD2 | 9.55E-01 |
| GO:0036465~synaptic vesicle recycling | 3 | 5.52E-02 | SYNJ1, RAB5A, SCRIB | 9.57E-01 |
| GO:0006399~tRNA metabolic process | 6 | 5.56E-02 | WARS, TYW1, TRMT10A, CDKAL1, FTSJ1, NSUN2 | 9.56E-01 |
| GO:0045682~regulation of epidermis development | 4 | 5.72E-02 | NCOA3, NAB2, MYSM1, TMEM79 | 9.58E-01 |
| GO:0006482~protein demethylation | 3 | 5.85E-02 | JMJD6, KDM8, JMJD1C | 9.58E-01 |
| GO:0008214~protein dealkylation | 3 | 5.85E-02 | JMJD6, KDM8, JMJD1C | 9.58E-01 |
| GO:0016055~Wnt signaling pathway | 10 | 5.96E-02 | SPIN1, NXN, RSPO1, CSNK1G2, RAB5A, PTPRU, FOXO3, LRRK1, TCF7L1, SCRIB | 9.58E-01 |
| GO:2001286~regulation of caveolin-mediated endocytosis | 2 | 6.18E-02 | PROM2, NEDD4L | 9.61E-01 |
| GO:0198738~cell-cell signaling by wnt | 10 | 6.24E-02 | SPIN1, NXN, RSPO1, CSNK1G2, RAB5A, PTPRU, FOXO3, LRRK1, TCF7L1, SCRIB | 9.60E-01 |
| GO:0042558~pteridine-containing compound metabolic process | 3 | 6.53E-02 | MTHFSL, MTHFD2, ALDH1L1 | 9.64E-01 |
| GO:0007219~Notch signaling pathway | 6 | 6.68E-02 | S1PR3, IL2RA, SORBS2, AAK1, DNER, MIB2 | 9.65E-01 |
| GO:0030307~positive regulation of cell growth | 6 | 6.82E-02 | CYBA, NCOA3, FGFR1OP, NOL8, NEDD4L, DBN1 | 9.65E-01 |
| GO:0032259~methylation | 9 | 7.08E-02 | DOT1L, ASH1L, NCOA6, SETMAR, SETD6, TRMT10A, FTSJ1, SETD2, NSUN2 | 9.68E-01 |
| GO:0046700~heterocycle catabolic process | 8 | 7.24E-02 | ALDH1L1, DCP2, SETMAR, GSPT2, AHCYL1, LSM1, DNASE1L2, CANT1 | 9.69E-01 |
| GO:1905114~cell surface receptor signaling pathway involved in cell-cell signaling | 11 | 7.28E-02 | SPIN1, NXN, RSPO1, CSNK1G2, RAB5A, PTPRU, FOXO3, DBN1, LRRK1, TCF7L1, SCRIB | 9.68E-01 |
| GO:1902035~positive regulation of hematopoietic stem cell proliferation | 2 | 7.37E-02 | PDCD2, THPO | 9.67E-01 |
| GO:0044270~cellular nitrogen compound catabolic process | 8 | 7.39E-02 | ALDH1L1, DCP2, SETMAR, GSPT2, AHCYL1, LSM1, DNASE1L2, CANT1 | 9.66E-01 |
| GO:0034109~homotypic cell-cell adhesion | 4 | 7.66E-02 | PIP5K1C, DSC2, PTPRU, SLC7A11 | 9.68E-01 |
| GO:0001558~regulation of cell growth | 10 | 7.80E-02 | CYBA, CTH, HNF4A, NCOA3, FGFR1OP, NOL8, CLSTN1, NEDD4L, 4932438A13RIK, DBN1 | 9.69E-01 |
| GO:0051235~maintenance of location | 8 | 8.44E-02 | PFN1, CYBA, PLA2G10, APOC4, GPSM2, PIP5K1C, 4932438A13RIK, DBN1 | 9.76E-01 |
| GO:0000075~cell cycle checkpoint | 6 | 8.52E-02 | DOT1L, SETMAR, CENPE, MAPKAPK2, TRRAP, NSUN2 | 9.75E-01 |
| GO:0019439~aromatic compound catabolic process | 8 | 8.57E-02 | ALDH1L1, DCP2, SETMAR, GSPT2, AHCYL1, LSM1, DNASE1L2, CANT1 | 9.75E-01 |
| GO:0045055~regulated exocytosis | 6 | 8.67E-02 | PFN1, YWHAZ, SYNJ1, RAB5A, SCRIB, TMEM79 | 9.74E-01 |
| GO:0007032~endosome organization | 3 | 8.73E-02 | AKTIP, SYNJ1, RAB5A | 9.74E-01 |
| GO:0006575~cellular modified amino acid metabolic process | 5 | 9.10E-02 | MTHFSL, MTHFD2, CTH, ALDH1L1, OPLAH | 9.76E-01 |
| GO:0072583~clathrin-mediated endocytosis | 3 | 9.12E-02 | HIP1R, SYNJ1, SCRIB | 9.76E-01 |
| GO:0006887~exocytosis | 8 | 9.46E-02 | PFN1, YWHAZ, SYNJ1, RAB5A, PIP5K1C, SDC4, SCRIB, TMEM79 | 9.78E-01 |
| GO:0044070~regulation of anion transport | 4 | 9.60E-02 | ARL6IP1, PLA2G10, AHCYL1, ACSL5 | 9.78E-01 |
| GO:0035520~monoubiquitinated protein deubiquitination | 2 | 9.70E-02 | ASXL1, MYSM1 | 9.78E-01 |
| GO:0018065~protein-cofactor linkage | 2 | 9.70E-02 | CTH, HMBS | 9.78E-01 |

Supplementary Table 4. A list of KEGG pathways with modified Fisher exact P-values less than 0.1 and their associated genes.

| Term | Count | P-Value | Genes | Benjamini |
| --- | --- | --- | --- | --- |
| *mmu04015:Rap1 signaling pathway* | 13 | 9.02.E-04 | FYB, PFN1, CDC42, PARD3, FGF7, FGF9, RAF1, FGF10, EFNA5, EFNA4, ARAP3, FGF2, LCP2 | 1.61.E-01 |
| *mmu04144:Endocytosis* | 15 | 9.82.E-04 | PARD3, IL2RA, KIF5A, PIP5K1C, VPS37D, RAB11FIP4, CDC42, ARF3, RAB35, RAB5A, RAB11A, SMURF2, NEDD4L, AGAP1, ARAP3 | 9.13.E-02 |
| *mmu00310:Lysine degradation* | 6 | 3.23.E-03 | DOT1L, ASH1L, SETMAR, ALDH2, SETD2, PHYKPL | 1.90.E-01 |
| *mmu04014:Ras signaling pathway* | 12 | 4.91.E-03 | CDC42, FGF7, PLA2G10, FGF9, RAB5A, RAF1, FGF10, NFKB1, EFNA5, EFNA4, PLA2G2E, FGF2 | 2.13.E-01 |
| *mmu04810:Regulation of actin cytoskeleton* | 11 | 8.69.E-03 | PFN1, CDC42, FGF7, ROCK1, FGF9, ABI2, RAF1, FGF10, PIP5K1C, ITGB5, FGF2 | 2.89.E-01 |
| *mmu05218:Melanoma* | 6 | 1.21.E-02 | FGF7, CDKN2A, FGF9, RAF1, FGF10, FGF2 | 3.26.E-01 |
| *mmu04151:PI3K-Akt signaling pathway* | 14 | 1.88.E-02 | YWHAZ, IL2RA, FGF7, FGF9, ITGB5, FGF10, RAF1, NFKB1, FOXO3, COL4A5, LAMA1, EFNA5, EFNA4, FGF2 | 4.10.E-01 |
| *mmu05200:Pathways in cancer* | 15 | 2.20.E-02 | LAMA1, CDC42, TCF7, FGF7, CDKN2A, HDAC1, ROCK1, FGF9, RAF1, FGF10, NFKB1, FGF2, TCF7L1, COL4A5, ARHGEF11 | 4.18.E-01 |
| *mmu04390:Hippo signaling pathway* | 8 | 2.75.E-02 | TCF7, PARD3, YWHAZ, FRMD6, CRB2, TEAD1, TCF7L1, SCRIB | 4.53.E-01 |
| *mmu04722:Neurotrophin signaling pathway* | 7 | 3.12.E-02 | CDC42, PRDM4, MAP3K1, RAF1, NFKB1, MAPKAPK2, FOXO3 | 4.61.E-01 |
| *mmu05412:Arrhythmogenic right ventricular cardiomyopathy (ARVC)* | 5 | 4.89.E-02 | TCF7, LMNA, ITGB5, DSC2, TCF7L1 | 5.89.E-01 |
| *mmu04520:Adherens junction* | 5 | 5.10.E-02 | MAP3K7, CDC42, TCF7, PARD3, TCF7L1 | 5.73.E-01 |
| *mmu00670:One carbon pool by folate* | 3 | 5.17.E-02 | MTHFSL, MTHFD2, ALDH1L1 | 5.49.E-01 |
| *mmu04010:MAPK signaling pathway* | 10 | 5.77.E-02 | MAP3K7, CDC42, FGF7, FGF9, MAP3K1, RAF1, FGF10, NFKB1, MAPKAPK2, FGF2 | 5.63.E-01 |
| *mmu00340:Histidine metabolism* | 3 | 7.84.E-02 | ASPA, ALDH2, AOC1 | 6.54.E-01 |
| *mmu05213:Endometrial cancer* | 4 | 7.98.E-02 | TCF7, RAF1, FOXO3, TCF7L1 | 6.37.E-01 |
| *mmu04932:Non-alcoholic fatty liver disease (NAFLD)* | 7 | 8.50.E-02 | CDC42, NDUFA8, PRKAB1, ADIPOR1, NFKB1, NDUFS1, UQCRB | 6.39.E-01 |
| *mmu04512:ECM-receptor interaction* | 5 | 9.16.E-02 | LAMA1, GP6, ITGB5, SDC4, COL4A5 | 6.47.E-01 |
| *mmu05221:Acute myeloid leukemia* | 4 | 9.47.E-02 | TCF7, RAF1, NFKB1, TCF7L1 | 6.40.E-01 |
