# Supplementary material for: Identification of genes required for eye development by high-throughput screening of mouse knockouts
Source: Commun Biol. 2018 Dec 21;1:236. doi: 10.1038/s42003-018-0226-0 (PMC6303268; doi:10.1038/s42003-018-0226-0)
Supplement: Supplementary file 2 — Description of Additional Supplementary Files [file 42003_2018_226_MOESM2_ESM.docx]

**Description of Additional Supplementary Files**

**File Name**: Supplementary Data 1

**Description**: Statistics for retinal thinning in Rnf10 knockout mice documented via optical coherence tomography. The 45 Datasheet provided showed statistics that revealed significant retinal thinning by 16 weeks postnatal age when compared to control retinal images. Box plots of male and female Rnf10-/- 46 mice document retinal thinning in comparison to normal age-matched controls, 47 particularly affecting the inner plexiform layer (IPL) and the inner nuclear layer (INL). Error bars within the associated figure (Figure 4 48 g and h) represent the standard deviation (SD) for the knockout measurements. Data points outside of the mean +/- SD range are 49 represented by black circles. The two dotted red lines delimit the reference range, defined as two SD away from the mean for 50 controls, represented by the solid red line.
